# Concurrent Gene Signatures for Han Chinese Breast Cancers

**DOI:** 10.1371/journal.pone.0076421

**Published:** 2013-10-03

**Authors:** Chi-Cheng Huang, Shih-Hsin Tu, Heng-Hui Lien, Jaan-Yeh Jeng, Ching-Shui Huang, Chi-Jung Huang, Liang-Chuan Lai, Eric Y. Chuang

**Affiliations:** 1 Graduate Institute of Biomedical Electronics and Bioinformatics, National Taiwan University, Taipei City, Taiwan; 2 Cathay General Hospital SiJhih, New, Taipei City, Taiwan; 3 School of Medicine, Fu-Jen Catholic University, New Taipei City, Taiwan; 4 School of Medicine, Taipei Medical University, Taipei City, Taiwan; 5 Department of Surgery, Cathay General Hospital, Taipei City, Taiwan; 6 Cathay Medical Research Institute, New Taipei City, Taiwan; 7 Graduate Institute of Physiology, National Taiwan University, Taipei City, Taiwan; University of Georgia, United States of America

## Abstract

The interplay between copy number variation (CNV) and differential gene expression may be able to shed light on molecular process underlying breast cancer and lead to the discovery of cancer-related genes. In the current study, genes concurrently identified in array comparative genomic hybridization (CGH) and gene expression microarrays were used to derive gene signatures for Han Chinese breast cancers.

We performed 23 array CGHs and 81 gene expression microarrays in breast cancer samples from Taiwanese women. Genes with coherent patterns of both CNV and differential gene expression were identified from the 21 samples assayed using both platforms. We used these genes to derive signatures associated with clinical ER and HER2 status and disease-free survival.

Distributions of signature genes were strongly associated with chromosomal location: chromosome 16 for ER and 17 for HER2. A breast cancer risk predictive model was built based on the first supervised principal component from 16 genes (*RCAN3*, *MCOLN2*, *DENND2D*, *RWDD3*, *ZMYM6*, *CAPZA1*, *GPR18*, *WARS2*, *TRIM45*, *SCRN1*, *CSNK1E*, *HBXIP*, *CSDE1*, *MRPL20*, *IKZF1*, and *COL20A1*), and distinct survival patterns were observed between the high- and low-risk groups from the combined dataset of 408 microarrays. The risk score was significantly higher in breast cancer patients with recurrence, metastasis, or mortality than in relapse-free individuals (0.241 versus 0, *P*<0.001). The concurrent gene risk predictive model remained discriminative across distinct clinical ER and HER2 statuses in subgroup analysis. Prognostic comparisons with published gene expression signatures showed a better discerning ability of concurrent genes, many of which were rarely identifiable if expression data were pre-selected by phenotype correlations or variability of individual genes.

We conclude that parallel analysis of CGH and microarray data, in conjunction with known gene expression patterns, can be used to identify biomarkers with prognostic values in breast cancer.

## Introduction

Breast cancer is a heterogeneous disease in terms of molecular taxonomy. Microarray experiments in the past decade have revealed distinct molecular subtypes based on gene expression patterns, most of which are associated with clinical phenotypes or predictions of treatment response or survival [1-10]. In contrast to gene expression signatures, the clinical significance of genomic aberrations in breast tumors, such as amplifications and deletions, remains undefined. It is widely acknowledged that cancer can result from progressive accumulation of genetic aberrations; amplified regions may contain dominant oncogenes whereas deleted regions may contain tumor suppressor genes. The breakpoints of recurrent aberrations may indicate novel targets of potentially therapeutic value [11].

Chromosomal comparative genomic hybridization (CGH) is a technique designed to assess genomic aberrations in tumor and cultured cells; however, the complexity of genomic variations as well as resolution limitations have impeded widespread application of this technique to breast cancer. Only recently has microarray-based CGH (array CGH), with either a BAC clone or oligonucleotide for hybridization, allowed the direct evaluation of the chromosomal instability of solid tumors [12-14].

It has been suggested that whole-genome array CGH can provide insight into the fundamental processes of chromosomal instability leading to breast cancer. For instance, van Beers et al. [15] performed a comprehensive review of breast cancer array CGH studies. They ascertained the reliability and sensitivity of this automated, high-resolution tool and gave examples in gene discovery and class discovery such as *BRCA1* and *BRCA2* breast tumors. For breast cancer cytogenetics with conventional CGH and the correlation with histological features, refer to Reis-Filho et al [16]. They described the strong link between CGH and underlying genetic changes and how it explains, in part, the molecular aspects that contributed to clinical pathological features in breast cancer patients.

Because genomic imbalance can have an impact on gene expression by complex transcriptional regulation, the interplay between copy number variation (CNV) and gene expression might shed light on underlying molecular processes in breast cancer and lead to the discovery of cancer-related genes. Genes displaying coherent patterns at the chromosomal and transcriptional levels are more likely to serve as biomarkers for treatment response and prognosis [17-20]. We hypothesized that breast cancer tumorigenesis could originate from chromosome instability manifesting as CNV, which could persist through mRNA transcription and present as concordant gene expression signatures.

In the current study, we performed genome-wide characterization of Han Chinese breast cancers by integrating 2 microarray technologies: array CGH to detect genomic CNV and gene expression arrays, to elucidate transcriptional alterations in an effort to reveal critical genes involved in breast oncogenesis and identify potential targets with prognostic value. Concurrent gene signatures for important pathological factors, as well as disease-free survival of breast cancer, were constructed, and prognostic comparisons with published biomarkers from gene expression only data were performed.

## Materials and Methods

### Ethics statement

The materials presented in the study have been reviewed and approved by IRB of Cathay General Hospital (approval number: CT-100035). Written informed consent was obtained from all the participants after well explanation by one of the five investigators (CCH, SHT, HHL, JYJ and CSH).

### Study design

**Figure 1:**
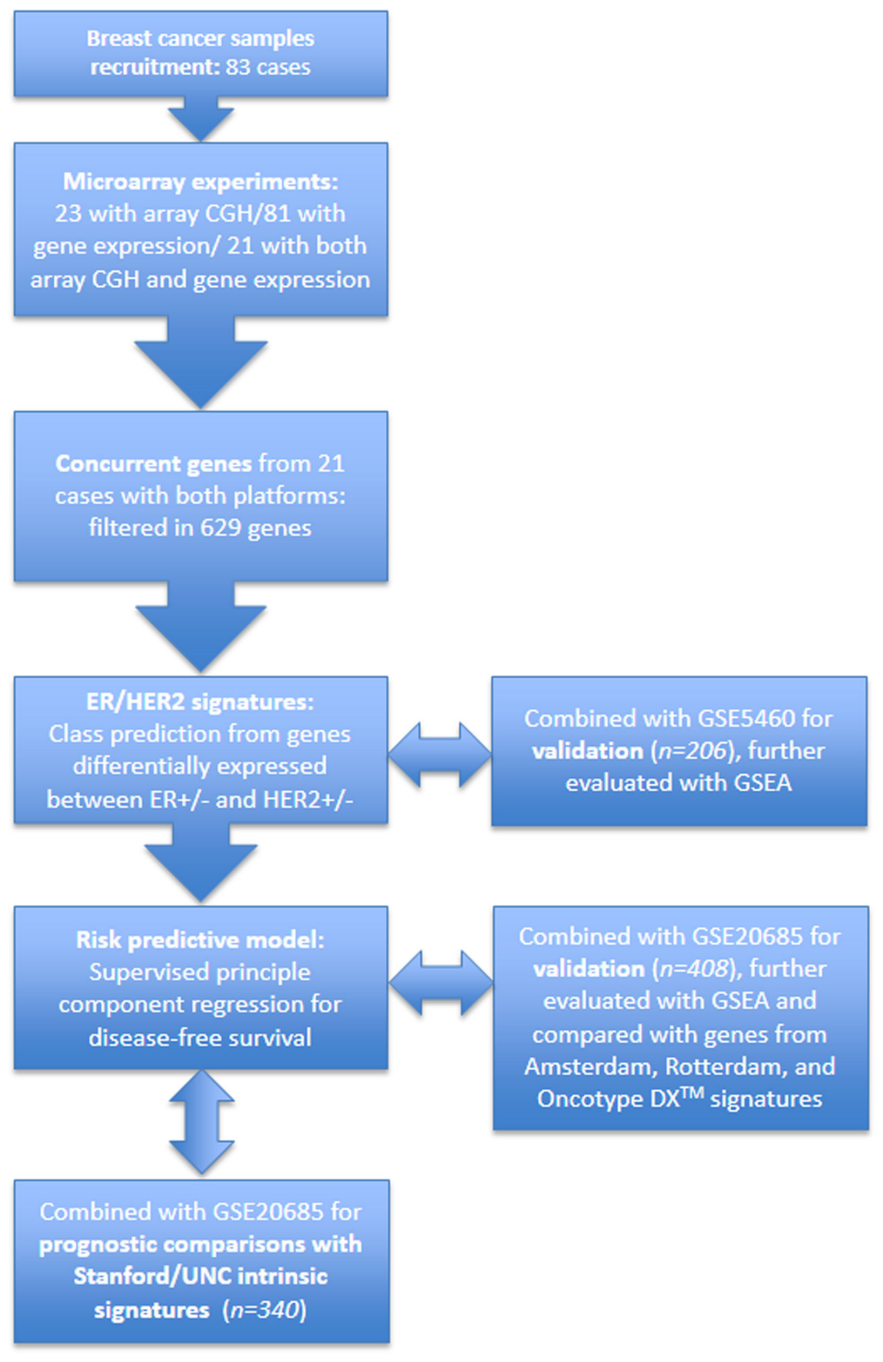
Flow chart of study design (GSEA: Gene set enrichment analysis).

A flow chart of the study design is shown in Figure 1.

### Breast cancer samples

We collected breast cancer samples prospectively during surgery. The cancerous and matched normal breast tissues were snap frozen and stored in liquid nitrogen below -80 °C, with RNAlater reagent (Qiagen, Germantown, MD) to stabilize RNA in the tissue. Matched samples were obtained consecutively from 83 breast cancer patients from January 2007 to December 2010. The frozen samples were dissected into slices of 1-2 mm thickness, and more than 90% cancerous content was a prerequisite for microarray experiments. The enrollment criteria included patients <70 years old with invasive breast cancers who had not received neo-adjuvant therapy, were in clinical stages I to III (i.e., no systemic spread, and had no concurrent secondary malignancy). Enrolled patients were managed according to standard guidelines with regular follow-up.

For relevant pathological features, estrogen receptor (ER) positivity was defined as the presence of at least 10% of nuclei with positive results by immunohistochemical (IHC) analysis, and breast samples displaying low ER positivity (<10% of nuclei with positive stains) were not assayed in the current study. For human epidermal growth factor receptor 2 (HER2) status, the ASCO and CAP guidelines [21] were followed: IHC 3+ and IHC 2+ with fluorescence in situ (FISH) hybridization amplification were considered to indicate HER2 overexpression. The modified Bloom-Richardson (Nottingham) system was used for grading breast cancers [22].

### Comparative genomic hybridization

DNA was extracted from cancerous and matching normal breast tissues from the same subject (n=21) by using a QIAamp DNA mini kit (Qiagen, Valencia, CA). A minimum of 4 µg total DNA was required for verifying the purity and concentration of genomic DNA, which was done using a Bioanalyzer 2100® (Agilent, Santa Clara, CA). DNA quality control was indicated by OD_260/280_ > 1.8, according to the manufacturer’s instructions. The Agilent Human Genome 105A® microarray was adopted for array CGH experiments; it includes 99,000 probes that span the human genome with an average spatial resolution of approximate 15 kb.

Genomic DNA from cancers and matching normal controls was labeled and hybridized to microarray slides for each study subject using the dye-swap technique. After hybridization, the slides were scanned with a GenePix® Scanner 4000B (Molecular Devices, Sunnyvale, CA), and the fluorescent dye ratios, which represented DNA copy number ratios, were obtained for data analysis. Gene information and genomic locations were based on Human Genome build 18 for Agilent CGH arrays.

### Copy number variation detection

The analysis of CGH began with segmentation of normalized data, followed by identification of common (recurrent) gains and losses across multiple array CGH experiments. The Circular Binary Segmentation (CBS) implemented in CGHTools of BRB ArrayTools was used to identify regions in each chromosome such that the copy numbers in each region were equal [23,24]. CBS can be considered as finding change points in a sequence of random numbers. For each chromosome, the data were recursively split until no further change points could be found. Determining whether a particular point corresponded to a change point was tested through permutations. A hybrid approach (Faster CBS) with the mergeLevels algorithm was used to speed up the process, and further details can be obtained from official CGHTools manuals [25,26]. Based on the segmented log ratios, the copy number at a particular genomic location was determined using the median absolute deviation (MAD) of the log ratios of each array. High level CNV (amplification and homozygous deletion) was declared in regions with segmentation mean log ratios >5 times the MAD and <0.2 times the MAD of the corresponding array. The multiplicative factor (threshold) for low level CNV (both gain and loss) was set to 1.1.

Regions with frequent CNV in a group of samples were identified using the Genomic Identification of Significant Targets in Cancer (GISTIC) tool in BRB-CGHTools [27]. The null distribution of the G scores was generated based on a 10,000 times re-sampling, and GISTIC identified frequent and significant CNV regions in all 23 array CGH samples. The significance of CNV at a particular genomic location was determined based on a test statistic obtained from the segmentation log ratios of all samples.

### Expression arrays

Total RNA was extracted from frozen specimens using TRIzol® reagent (Invitrogen, Carlsbad, CA). Purification of RNA was performed using RNeasy® mini kits (Qiagen, Valencia, CA) according to the manufacturer’s instructions. RNA integration was determined by performing gel electrophoresis; 2 bands of 18S and 28S represented satisfactory RNA quality. Affymetrix GeneChip® Human Genome U133 plus 2.0 (Affymetrix, Santa Clara, CA) was used for the microarray experiment. Hybridization and scanning was performed according to the standard Affymetrix protocol. In brief, there were more than 54,000 probe sets, 47,400 transcripts and approximately 38,500 genes on this single color oligonucleotide array. Image scanning was performed using a GeneChip® Scanner 3000 (Affymetrix, Santa Clara, CA), and scanned images were processed using GeneChip® Operating Software and Affymetrix’s Microarray Suite software to generate detection *P* values. The Robust Multichip Average (RMA) algorithm was applied for perfect match probe signals within the study [28]. For multiple probe sets corresponding to the same gene, probe sets were reduced to one per gene symbol by using the most variable probe set measured by inter-quadrant range across all the assayed arrays. Deposition of microarray data at the NCBI Gene Expression Omnibus had made both array CGH and gene expression experiments publicly available with the accession number GSE48391.

### Concurrent gains and losses

Concurrent gains and losses were detected from common probes across array CGH and gene expression experiments. We integrated gene expression and array CGH data to identify genes whose transcriptional levels were affected by CNV. Concurrent gains and losses were declared if and only if significant changes in the same direction were observed for both gene expression and array CGH platforms (assessed by Spearman correlation coefficients with cut-off *P*-value <0.05). Specifically, a gene-centric table was created to deduce a value corresponding to each gene for each array in the array-covered genomic regions. This value was assigned based on the segmentation mean log ratios, and was used to calculate the correlation between copy number and gene expression during integrated analysis.

### Combined dataset

Two publicly available breast cancer microarray depositories, one from Lu et al. and another from Kao et al., were merged with our microarrays to form the combined dataset [29,30]. Both datasets used the same Affymetrix U133 plus 2.0 microarrays as used in our experiments, and all assayed subjects were Han Chinese ethnically. RMA was used for normalization within each dataset [28]. Details of microarray experiments and the demography of the study populations have been described elsewhere and only a brief summary is given here. The Lu et al. dataset comprised 125 Chinese breast cancers, and original Affymetrix *CEL* files were downloaded from NCBI Gene Expression Omnibus with the accession number GSE5460; clinical ER and HER2 status was provided, while IHC 2+ was considered HER2 negative, and low ER positivity (1-9% of nuclei with positive stains) was regarded as ER positive in their series. For the Kao et al. dataset, 327 Taiwanese breast cancers were assayed, and corresponding disease-free survival and overall survival data were available (GSE 20685). To make microarray experiments from different studies comparable, we first normalized (centered) each array using the median array within each group (i.e., the median array in each group was used as the reference) before further analysis was performed. When the combined dataset was constructed, the processed expression profiles of breast cancers from our series and the independent studies were pooled together, and quantile normalization was performed to remove the batch effect.

### Concurrent signatures for ER and HER2

Concurrent genes were identified as previously described, and those that were differentially expressed in conjunction with distinct ER and HER2 status were identified using univariate two-sample t-tests at a 0.001 significance level. A global multivariate permutation test with a stringent α level of 10^-3^ was performed to control for false positives. Differentially expressed concurrent genes were further used to predict clinical phenotype by multiple methods including compound covariate predictor, diagonal linear discriminative analysis, 3 nearest neighbors, nearest centroid, and support vector machine (with default penalty of LIBSVM, see references 24,31). Clinical phenotype was treated as the gold standard when predictive accuracy was evaluated through “leave one out” cross-validation. When class prediction across microarray studies was performed in the combined dataset, one additional random effect was added to account for the bias introduced by each additional source of experiments. More details about classification algorithms were detailed in Methods S1. Concurrent signatures for ER and HER2 were developed independently in both our microarrays and those from Lu et al., each time with one dataset for training and the other for independent validation. Finally signatures composed of consensus genes (the intersection of signature genes across studies) were tested for predictive accuracy in the combined dataset.

### Breast cancer risk (survival) predictive model

Supervised principal component regression was used to build a breast cancer risk predictive model [32]. Initially, genes with predictive value were identified by a univariate Cox proportional hazards model, and significant genes within a stringent α level of 0.001 were further used to synthesize the first principal component. This first principal component (supergenes) was used in the predictive model construction. In our study, disease-free survival (with tumor recurrence or metastasis as the first failure event) was measured, and only the first principal component was adopted for ease of interpretation. A (continuous) prognostic index score was calculated for each subject according to regression coefficients for the first principal component, intercept, and (any) covariate from the Cox model. The high- and low-risk groups were defined by the 50th percentile prognostic index. We also compared the prognostic value of concurrent genes to that of intrinsic gene signatures provided by the Stanford/UNC team. Molecular subtyping of the combined dataset and subgroup analysis stratified by clinical ER and HER2 status was performed [33]. Besides, we also constructed risk predictive models based on reported genes from published breast cancer gene expression signatures, including Amsterdam, Rotterdam, and Oncotype DX^TM^ signatures, using the same supervised principle component approach; more details are available in the Methods S1 [8,9,34,35]. The impact of high-/low-risk threshold definition was evaluated through sensitivity analysis.

### Gene set enrichment analysis for concurrent genes

Gene set enrichment analysis (GSEA, reference [36]) is a functional analysis of microarray data at the level of gene sets. GSEA tested whether an a priori defined set of genes showed statistically significant and concordant difference between two biological states (in current study, the dichotomous status of clinical ER, HER2, and cancer relapse). We performed GSEA and concurrent gene signatures were regarded as gene sets for two purposes. First, if a concurrent gene signature was significantly enriched between the two corresponding phenotypes, the discerning ability of this signature would be evidenced (indicating by a normalized enrichment score with a significant deviation from zero under a controlled false discovery rate) and GSEA provided an additional validation for this signature. Second, all genes on the microarray were sorted by some clinical feature (ER, HER2, and relapsing status) and a ranked list was created under GSEA, which allowed the precise localization of each signature gene along the full length of this ranked list. In many circumstances, genes were selected/filtered by their phenotype correlations; if a signature gene was not found at the top (up regulated) or bottom (down regulated) of the ranked list, the possibility would be high that such a candidate gene could not be easily identified by relevant filtrating/selection criteria. Some gene expression studies used the variability of each array element for gene filtering; a GSEA pre-ranked list weighted by coefficient of variance could determine whether a candidate gene was located at the beginning (high variability) end of the list and was readily enrolled for down-stream analysis. More details about compositions of concurrent gene sets were in Methods S1.

## Results

### Analysis of array CGH

A total of 83 incidental breast cancer samples were recruited in a consecutive manner between January 2007 and December 2010. Of these, 23 underwent array CGH, 81 underwent expression microarrays, and 21 were assayed with both platforms. Relevant clinical features are listed in Table 1.

**Table 1:** Clinical features of 81 Taiwanese breast cancers.

		Array CGH only	Both array CGH and gene expression	Gene expression only
Number of cases		2	21	60
ER	Positive	1	10	43
	Negative	1	11	17
HER2	Overexpression	0	10	24
	Normal	2	11	36
Nuclear grade	I	0	1	4
	II	1	8	32
	III	1	12	24
Lympho-vascular invasion	Positive	0	13	39
	Negative	2	8	21
Nodal status	Positive	0	13	35
	Negative	2	8	25

**Figure 2:**
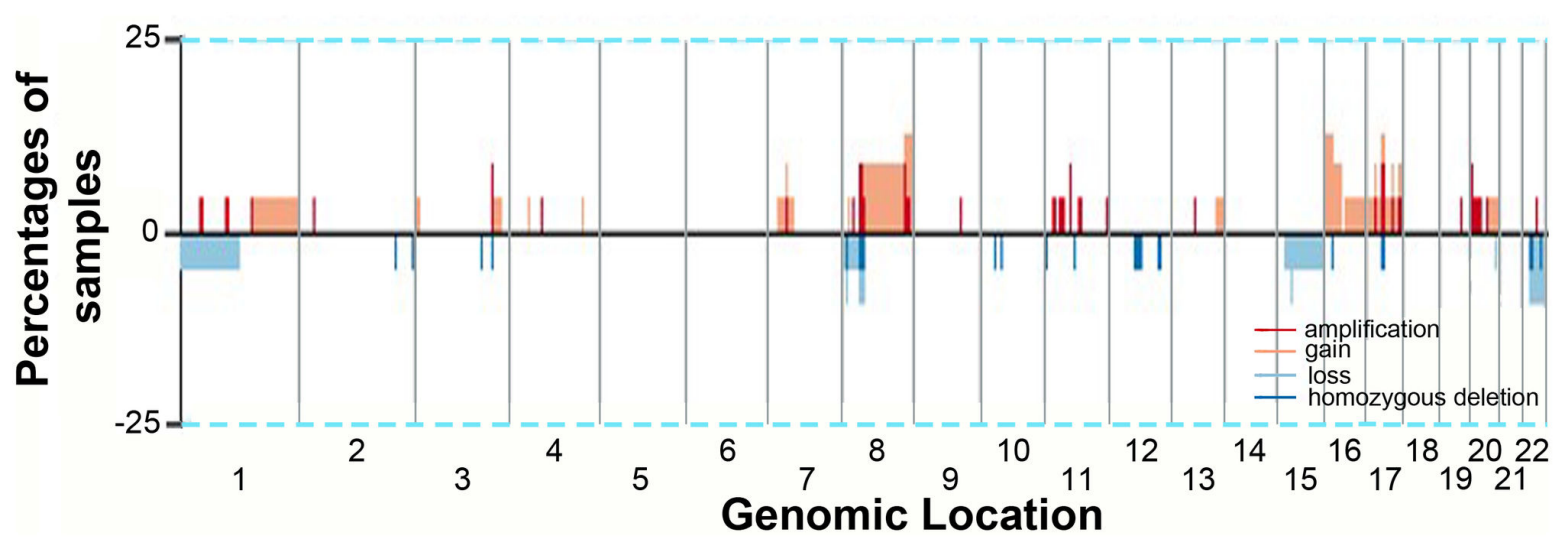
Frequency plot of copy number variation in a sample of 23 Taiwanese breast cancers.

The CBS algorithm changed normalized array CGH data into discrete segments of equal chromosomal copy number [23-26]. The number of unique markers was 98,755 with a significance level for the test to accept change points set to 0.01 with 1,000 permutations. One hundred and three CNVs were claimed including 12 amplifications, 51 gains, 32 losses and 8 homozygous deletions (Table 2). Figure 2 shows the frequency plot of CNV among 23 samples stratified by genomic location; the most common CNV was gains/amplifications of chromosome 8, followed by gains in chromosome 16, 17, and 20. Recurrent amplifications were 3q26.1 (n=3), and 17q11-q21 (n=2) while recurrent gains included 8p11 (n=5), 8p12 (n=5), 8q11-q24 (n=4), 8q24 (n=5), 17q25 (n=3), 20p12 (n=3), and 20q13 (n=3). Less frequent gains (n=2) were 1p21.2, 3q26.1, 8p23.3, 11p12, 11q11, 16p12-p13, and 20q11-q13. Complex recurrent deletions were reported for various lengths around 8p11-p23 (n=9) as well as 22q11-q13 (n=5); less common deletions (n=2) were 15q13 and 20q13. There was no recurrent homozygous deletion.

**Table 2:** Details of CNVs (copy number variations) among 23 Taiwanese breast cancers.

	**Amplification**	**Gain**			**Loss**		**Homozygous deletion**
Number of CNVs	12	51			32		8
Number of involved subjects	8	13			8		5
Number of involved genes	95	2221			2863		76
Cytogenetic locations*	1q21.2	**1p21.2**	8p23.2	17q23	1p11.1-p36.33	12q23-q24.11	2q37.3
	**3q26.1**	**1p21.2-p22**	**8p23.3**	**17q24-q25**	2q33	**15q11-q26.3**	3q26.1
	**3q26.1**	1p32-p35	**8p23.3**	**17q25.1**	2q37.3	**15q13.3**	8p11.1-p12
	**3q26.1**	2p22.3	**8q11-q24**	**17q25.3**	3q22-q24	16p12.3	8p23.1
	4q13	3p24-p26	**8q11-q24**	**20p11-p12**	5q35.3	17q11-q22	10q26.3
	8q24	**3q26-q27**	**8q11-q24**	**20p11-p13**	**8p11.1-p21**	**20q13**	11p11-p12.3
	9q22.3	**3q26.1**	**8q11-q24**	**20p12-p13**	**8p11.21-p12**	**20q13.31**	22q11.23
	11q24-q25	4q13.2	**8q24**	**20q11-q13**	**8p11.22-p11.23**	**22q11.1**	22q13.31
	**17q11-q21**	4p14	**11p12**	**20q11-q13**	**8p11-p12**	**22q11-q13.31**	**17q11-q21**
	**17q11-q21**	4q31.3	**11p12-p13**	**20q13**	**8p11-p23**	**22q11.1-13.33**	**17q11-q21**
	19q13.2	7p11-p21	11p15.1	22q12.1-q12.3	**8p11-p23.3**	**22q11-q13.33**	19q13.2
	20q11.21-q11.23	7p13	**11q11**		**8p12**	**22q13.1**	20q11.21-q11.23
		**8p11-p12**	**11q11**		**8p21.3-p22**		
		**8p11.2**	11q13-q14		**8p22-p23.3**		
		**8p11.21**	13q14.2-q14.3		10q11.21		
		**8p11.2-p12**	13q31.2-q34		10p12		
		**8p11.2-p12**	**16p11-p13**		11p15		
		**8p12**	**16p12-p13**		**12q13-q14.1**		
		**8p12**	17p11.2		**12q13-q14.1**		
		8p22	17q21.31		**12q13-q21**		

(* cytogenetic locations in boldface: segments with recurrent CNVs)

We used the GISTIC method to identify frequent CNV among 23 patients with array CGH [24,27]. The analysis tool identified frequent and significant CNV regions with a 0.05 false discover rate. The most frequent gain region with significant G-scores was at 8q24.21 to 8q24.23 with 34 genes, followed by 17q12 to 17q21.1 with 24 genes. For a complete list of genes with significant GISTIC scores, please refer to Table S1.

### Correlations between CNV and gene expression profiles

Both array CGH and gene expression data were available for 21 subjects, and these paired samples formed the basis of the integrated analysis of CNV and gene expression. Spearman correlation coefficients were calculated for 4,081 genes with CNV, and 629 of them were found to also have differential gene expression on the microarray. These genes formed the starting material for downstream analysis (Table S2 and Figure S1).

### ER signature

Gene expression signatures for the ER were identified independently from our 81 Taiwanese breast cancers and the 125 Chinese breast cancers from the Lu et al. dataset (GSE5460), with clinical IHC results used as the gold standard. Genes differentially expressed between ER-positive and -negative tumors were selected by two-sample t-tests with random variance (α-level: 0.001), and the derived ER classifiers were tested with multiple methods (Table S4A; more details in Methods S1).

The consensus of ER signatures from the 206 combined breast cancer samples resulted in a list of 36 genes, including *NME3*, *ADCY9*, *WDR90*, *IKBKB*, *SRP14*, *WWP1*, *GPR160*, *ERI2*, *CDIPT*, *TCEA3*, *FLJ10661*, *S100PBP*, *GLIS2*, *FLYWCH2*, *METRN*, *TRNAU1AP*, *RSC1A1*, *TRIM45*, *HAGH*, *FDXR*, *C16orf52*, *ZNF720*, *STK40*, *SLCO4A1*, *ELOVL1*, *ADRM1*, *PDZK1IP1*, *CHCHD10*, *SMCR7L*, *WDR77*, *RTN4R*, *THOC5*, *HPDL*, *HENMT1*, *UQCRH*, and *MRPL37*. The predictive accuracy was 89-93% across the various validation methods (Table 3). The most frequent genomic locations of ER signature genes were chromosome 16 (*NME3*, *ADCY9*, *WDR90*, *ERI2*, *METRN*, *GLIS2*, *FLYWCH2*, *HAGH*, and *C16orf52*) and chromosome 8 (*IKBKB* and *WWP1*). The use of the consensus ER signature genes resulted in a classifier with parsimony and generalizability for that fewer genes were enrolled, model over-fitting was improved, as well as the reduced predictive discrepancy among multiple classification methods.

**Table 3:** Predictive accuracy of the ER and HER2 signature in 206 Han Chinese breast cancers.

Classification algorithm	ER+/ER-	HER2+/HER2-
	127/79	64/142
Compound covariate predictor	93%*	85%*
Diagonal linear discriminative analysis	92%	85%*
3-nearest neighbors	91%	82%
Nearest centroid	92%	84%
Supportive vector machines	89%	84%

(* best predictive results)

### HER2 signature

Genes differentially expressed between HER2-overexpressing tumors and those with normal HER2 status were identified from two Han Chinese breast cancer data sets using two-sample t-tests and with α-level of 10^-3^. The consensus gene set making up the HER2 signature was *C17orf37*, *STARD3*, *ERBB2*, *PSMD3*, *PGAP3*, *GRB7*, *MED24*, *ORMDL3*, and *CDK12*. The predictive accuracy of the consensus HER2 signature was 82-85% during cross-validation (Table 3 and Table S4B). All 9 genes of the HER2 signature reside on chromosome 17. As observed for ER signature, using HER2 consensus genes improved overall predictive accuracy; the performances across multiple methods were enhanced with fewer genes adopted for the classifier.

### Survival prediction model

The median follow-up time of the 81 breast cancer patients with expression data was 3.7 years (range: 0.1 to 5.8 years) with 13 events of recurrence, metastasis, or breast cancer-specific mortality (16%) and 11 deaths (all-cause mortality). For 327 breast cancers from Kao et al. (GSE20685), the median follow-up was 7.7 years with 94 events of recurrence, metastasis, or breast cancer-specific mortality (29%), and 83 deaths (all-cause mortality).

**Figure 3:**
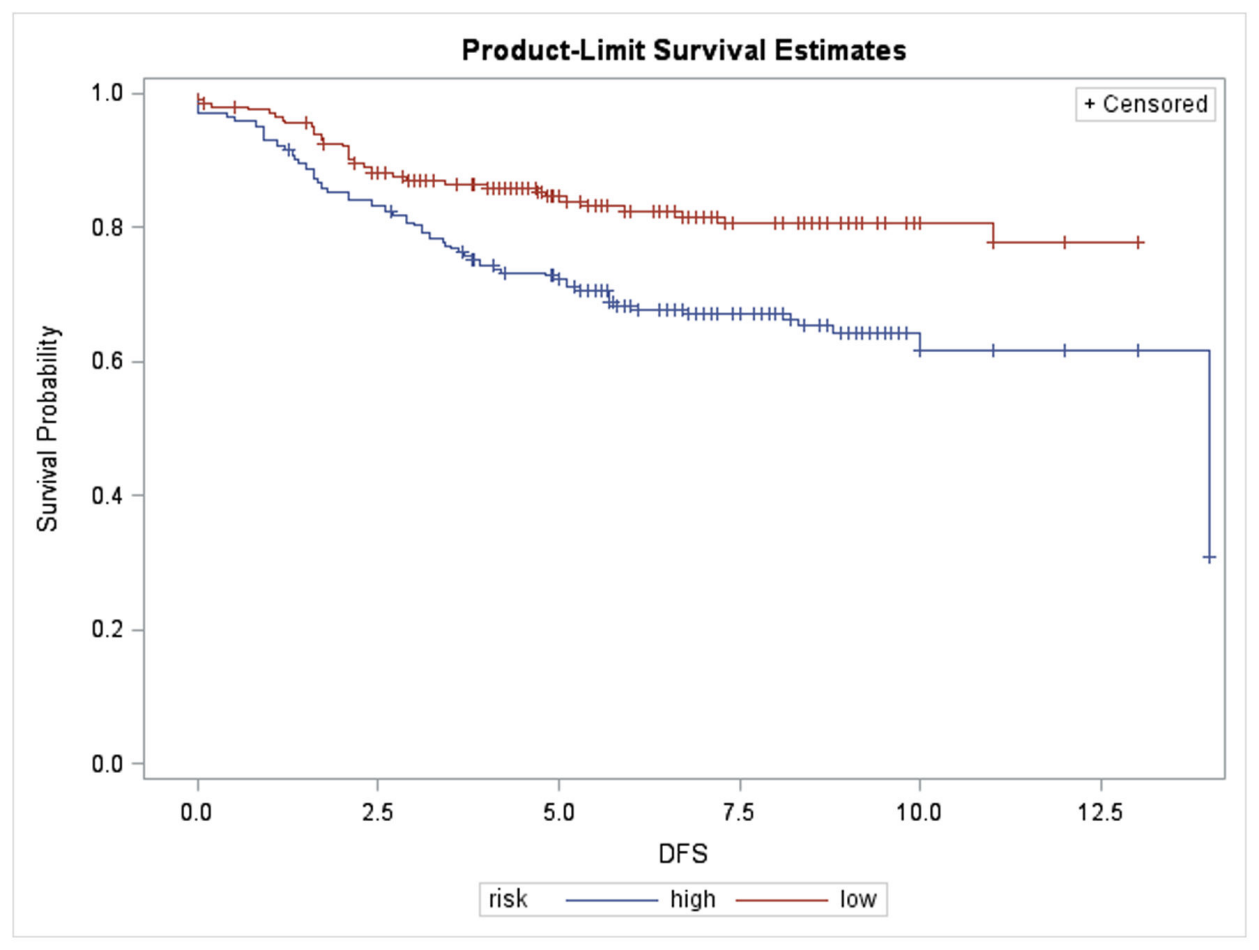
Disease-free survival in 408 Han Chinese breast cancer patients stratified by the 16-concurrent gene signature (Log-rank test: *P* <0.001). X-axis: survival time in years.

The combined dataset of 408 microarrays of Han Chinese breast cancers was constructed by merging our experiments with arrays from Kao et al. The median follow-up time was 6.5 years with 107 events of recurrence/metastasis/breast cancer-specific mortality (26%). One random effect was added to account for the batch effect and sample size discrepancy from two patients’ sources. A 16 gene signature, including *RCAN3*, *MCOLN2*, *DENND2D*, *RWDD3*, *ZMYM6*, *CAPZA1*, *GPR18*, *WARS2*, *TRIM45*, *SCRN1*, *CSNK1E*, *HBXIP*, *CSDE1*, *MRPL20*, *IKZF1*, and *COL20A1*, was selected by the univariate Cox proportional hazards model and was used to form the first supervised principal component for risk group prediction (Figure 3, log-rank test: *P* = 0.001 stratified by high- and low-risk group defined by the 50^th^ percentile). For the 301 disease-free patients, the mean prognostic index score was 0.000 and 133 cases (44%) were predicted to be high-risk. For the 107 patients with recurrence, metastasis or mortality, the mean prognostic index score was 0.241 and 71 patients (66%) were categorized as high-risk (*P* = 0.001, χ^2^-test). The difference in risk score between subjects with and without recurrence/metastasis/mortality in their follow-up was significant (*P* <0.001, two-sample t-test with unequal variance). The formula for the prognostic index score calculation is:

Prognostic index score=

RCAN3*(-0.027) +MCOLN2*(-0.13) +DENND2D*(-0.047) +
RWDD3*(-0.019) +ZMYM6*(-0.022) +CAPZA1*(-0.029) +TRIM45*(0.049) +
GPR18*(-0.156) +WARS2*(0.011) +SCRN1*(0.013) +CSNK1E*(0.011) +
HBXIP*(-0.007) +MRPL20*(-0.004) +CSDE1*(-0.017) +COL20A1*(0.001) +
IKZF1*(-0.179) +batch*(0.079) +3.9

For samples from Kao et al., a coefficient of 0.079 was added to control for the batch effect. The threshold for classification as high- or low-risk was 0.122 (determined from the 50th percentile of all the 408 arrays). Figures S2-S4 show the clustering results of signature genes for ER, HER2, and the risk predictive model, respectively.

### Prognostic comparisons between concurrent genes and Stanford/UNC intrinsic genes

The prognostic value of the breast cancer risk predictive model based on concurrent genes was compared with the intrinsic taxonomy proposed by the Stanford/UNC group [2-4]. The combined data for 408 Han Chinese breast cancers were categorized into one of the five molecular subtypes based on 306 intrinsic genes proposed by Hu et al [5]. The method of molecular subtyping has been described previously [33], and the distributions of intrinsic subtypes and corresponding clinical phenotypes are shown in detail in Table S3A-S3C. It should be noted that IHC results of 327 breast cancers from Kao et al. were not available, and we modified the method from Karn et al. [37] and fitted two finite mixture models to derive clinical ER and HER2 status directly from the corresponding probesets; more details are given in the Methods S1.

**Figure 4:**
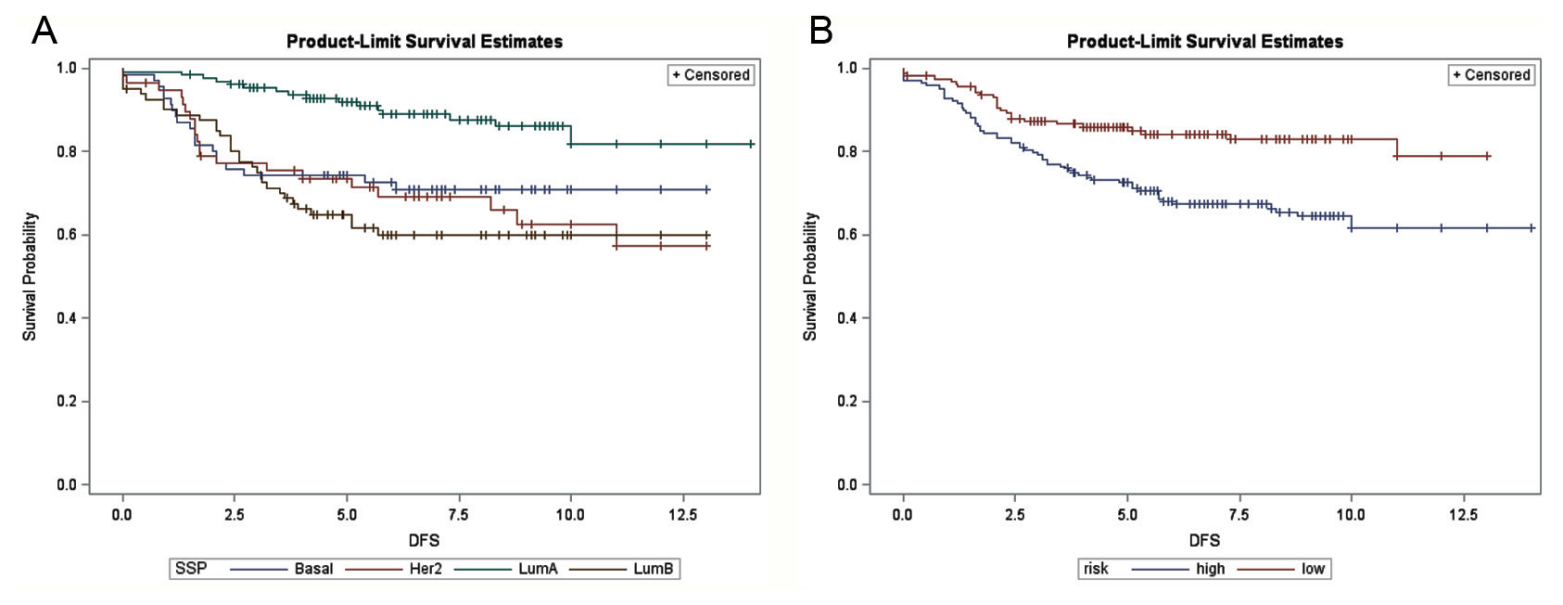
Disease-free survival in 340 Han Chinese breast cancer patients. (A) Stratified by intrinsic subtypes (proportional hazards assumption violated) and (B) stratified by the 16-concurrent gene signature (Log-rank test: *P* <0.001). SSP: single sample prediction, Basal: basal-like, Her2: HER2-enriched, LumA: luminal A, LumB: luminal B subtype breast cancer. X-axis: survival time in years.

Figure 4A shows the disease-free survival associated with the 340 Han Chinese breast cancers, stratified by 4 molecular subtypes after discarding 68 normal breast-like or unclassified cases from the combined dataset of 408 microarrays. The luminal A subtype had the highest probability of disease-free survival, while the prognoses of luminal B, basal-like, and the HER2-enriched subtype were hard to separate. Figure 4B shows the same plot stratified by concurrent genes identified in our study. The prognostic value of the 16 concurrent genes was evident herein.

**Figure 5:**
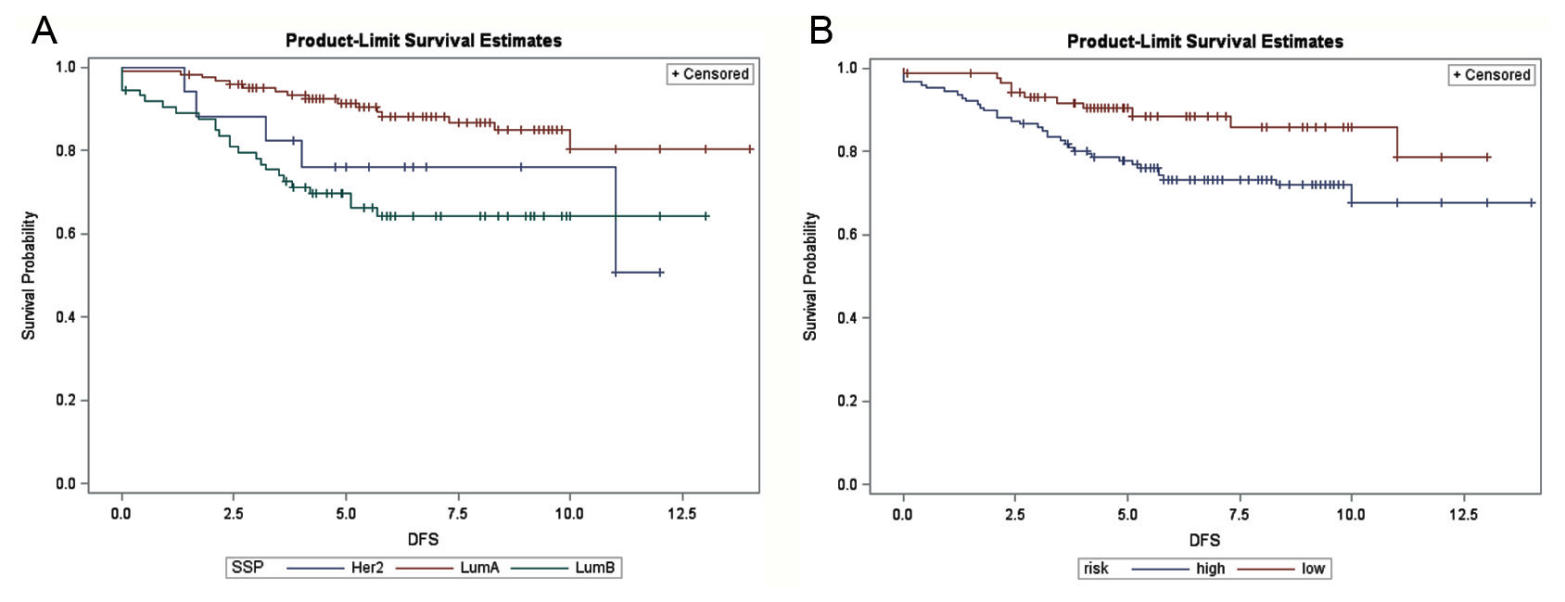
Disease-free survival in 214 ER-positive Han Chinese breast cancer patients. (A) Stratified by intrinsic subtypes (proportional hazards assumption violated) and (B) stratified by the 16-concurrent gene signature (Log-rank test: *P* = 0.023). SSP: single sample prediction, Her2: HER2-enriched, LumA: luminal A, LumB: luminal B subtype breast cancer. X-axis: survival time in years.

**Figure 6:**
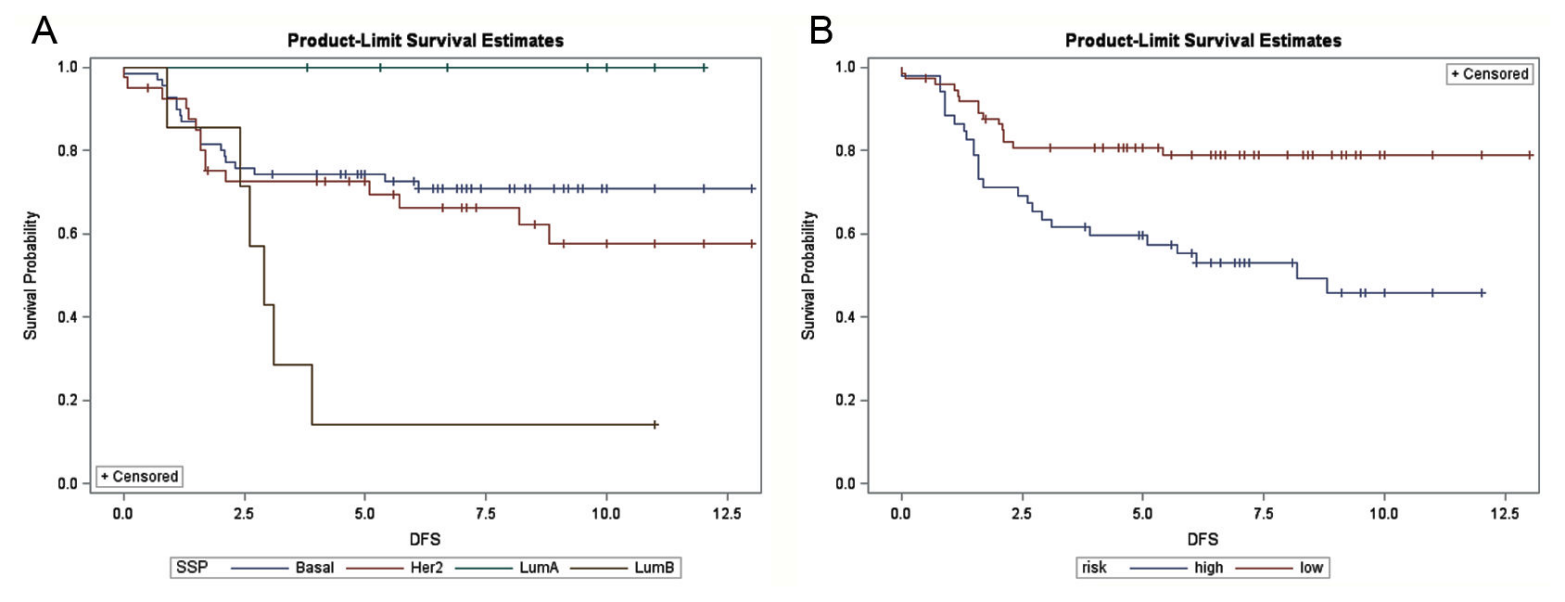
Disease-free survival in 126 ER-negative Han Chinese breast cancer patients. (A) Stratified by intrinsic subtypes (proportional hazards assumption violated) and (B) stratified by the 16-concurrent gene signature (Log-rank test: *P* <0.001). SSP: single sample prediction, Her2: HER2-enriched, LumA: luminal A, LumB: luminal B subtype breast cancer. X-axis: survival time in years.

Subgroup analyses were further performed for ER-positive (n = 214) and ER-negative breast cancers (n = 126) separately. The luminal A outperformed the luminal B subtype in ER-positive breast cancers, while for ER-negative patients, the prognostic discrepancy between HER2-enriched and basal-like breast cancers were indistinguishable (Figures 5A and 6A). In both cases, the probability of survival predicted by the concurrent genes was clearly distinguishable for the high- and low-risk groups in both ER-positive and -negative tumors (Figures 5B and 6B). Similarly, the better prognosis of the low-risk group as defined by concurrent genes was ascertained regardless of clinical HER2 status, which was not the case for intrinsic genes in subgroup analyses of HER2 overexpressing breast cancers (n = 95) and breast cancers with normal HER2 status (n = 245) (Figure S5A-S5B and S6A-S6B, respectively).

### Prognostic comparisons between concurrent genes and genes reported in Amsterdam/Rotterdam/Oncotype DX^TM^ signatures

To further validate the prognostic values of concurrent genes, predictive models composed of genes identified by other gene expression signatures, including Amsterdam, Rotterdam, and Oncotype DX^TM^, were compared with concurrent genes’ model on the same cohort of 408 Taiwanese breast cancers. Details of retrieving signature genes can be found in Methods S1.

These signature genes served as candidate variables and were further selected by the univariate Cox proportional hazards model and selected genes were used to synthesize the first supervised principle component for breast cancer risk prediction. However, the significance level of the univariate Cox regression was relaxed to 0.01, as opposed to the more stringent 0.001α level for the concurrent genes, in an effort to provide more predictive variables for supervised principle component model. Table 4 showed that for the Amsterdam signature genes, the low-risk group reported even more cancer relapses than the high-risk group (χ^2^-test: *P*=0.009), which was contradictory and was further evidenced by the troublesome area under the curve (AUC) value of 0.492 during the receiver operating characteristics (ROC) analysis (Figure S7A-S7D). The relapsing rate between the high- and low-risk groups was not significant for both the Rotterdam and Oncotype DX^TM^ signature genes, while the risk prediction by concurrent genes provided the best discriminative power (relapsing rate: 34.8 versus 17.7%, χ^2^-test: *P* < 0.001) and the highest AUC value (0628).

**Table 4:** Prognostic comparisons between concurrent genes and other signature genes using supervised principle component risk predictive model with high-/low-risk threshold set to the 50^th^ percentile of prognostic index score.

Signature	Risk prediction	Relapsing rate		*p*-value(χ^2^-test)	AUC*
Amsterdam	High-risk	20.67%	(43/208)	0.009	0.492
	Low-risk	32%	(64/200)		
Rotterdam	High-risk	27.83%	(59/212)	0.4434	0.543
	Low-risk	24.49%	(48/196)		
Oncotype DX^TM^	High-risk	27%	(54/200)	0.7273	0.556
	Low-risk	25.48%	(53/208)		
Concurrent genes	High-risk	34.80%	(71/204)	<0.0001	0.628
	Low-risk	17.65%	(36/204)		

(* AUC: area under the curve)

To further guard comparative results and evaluate the impact of the high-/low-risk threshold on predictive accuracy, we re-run each survival predictive models with the threshold set to the 30^th^ percentile of prognostic index score, approximating the true relapsing rate of 26% of the 408 Taiwanese breast cancer cohort. Table 5 showed the results of this sensitivity analysis; risk predictive model of purposed concurrent genes delivered the best discriminative power while borderline significance was observed for the model driven by the Oncotype DX^TM^ signature genes.

**Table 5:** Prognostic comparisons between concurrent genes and other signature genes using supervised principle component risk predictive model with high-/low-risk threshold set to the 30^th^ percentile of prognostic index score.

Signature	Risk prediction	Relapsing rate		*p*-value(χ^2^-test)	AUC*
Amsterdam	High-risk	26.82%	(81/302)	0.6443	0.492
	Low-risk	24.53%	(26/106)		
Rotterdam	High-risk	25.71%	(72/280)	0.7284	0.543
	Low-risk	27.34%	(35/128)		
Oncotype DX^TM^	High-risk	28.62%	(83/290)	0.0847	0.556
	Low-risk	20.34%	(24/118)		
Concurrent genes	High-risk	31.23%	(89/285)	0.0005	0.628
	Low-risk	14.63%	(18/123)		

(* AUC: area under the curve)

### GSEA for concurrent gene signatures

Signatures of ER, HER2 and breast cancer survival prediction model were further evaluated by GSEA with these concurrent gene sets. Each signature was split into two parts, which contained either the up regulated or down regulated signature genes (except for HER2 signature, for which only up regulated part was available; more details in Methods S1). The ER and HER2 signatures were tested in our 81 Taiwanese breast cancers and 125 Chinese breast cancers from Lu et al. data set (GSE5460) with the clinical ER and HER2 status used as phenotype labels. The gene set for breast cancer survival predictive signature was tested in our cohort and 327 Taiwanese breast cancers from Kao et al (GSE20685). Table 6 showed that three-fourths of both the ER and survival predictive gene sets were significantly enriched across microarray studies, indicating an alternative validation of purposed signatures in addition to the antecedent leave-one-out cross validation. However, HER2 signature gene set was compromised with high false discovery rates, which might be attributed to relatively fewer genes within this gene set.

**Table 6:** Gene set enrichment analysis (GSEA) with concurrent gene sets of ER, HER2, and survival predictive model.

Phenotype	Dataset	Enriched gene set^&^	NES^*^	Nom *p*-value^*^	FDR *q*-value^*^
ER positive	Current study	**Up-regulated in ER+**	2.03	0	0
	GSE5460	**Up-regulated in ER+**	1.23	0	0
ER negative	Current study	Down-regulated in ER+	-1.84	0	1
	GSE5460	**Down-regulated in ER+**	-1.41	0	0.159
HER2 overexpression	Current study	**Up-regulated in HER2+**	1.17	0	0.216
	GSE5460	Up-regulated in HER2+	1.2	0	0.486
HER2 normal	Current study	N/A		N/A	N/A
	GSE5460	N/A		N/A	N/A
Relapsing patients	Current study	Up-regulated in survival predictive (relapsing status)	0.62	1	1
	GSE20685	**Up-regulated in survival predictive (relapsing status)**	1.72	0	0
Disease-free patients	Current study	**Down-regulated in survival predictive (relapsing status)**	-1.55	0	0.038
	GSE20685	**Down-regulated in survival predictive (relapsing status)**	-1.39	0	0.098

(& Gene sets in boldface: enriched gene sets with significant nominal p-values and FDR q-value < 0.25. For details of each get set, refer to Methods S1. *NES: normalized enrichment score, Nom p-value: nominal p-value, FDR q-value: false discovery rate q-value, and N/A: not applicable)

Figures S8A **-**S8J showed GSEA plots of three signature gene sets (split into up and down regulated genes for each signature, except the HER2 signature for which all genes were up regulated) across microarray studies, including enrichment scores, gene tags (locations where members of the pre-defined gene set were found), and the correlation coefficient with the phenotype, along the ranked list by phenotype correlations.

Intuitively, most gene tags would be expected at the extremities of both the positive correlation (coefficient of 1) and negative correlation (coefficient of -1) end of the ranked list metric, depending on whether the up or down regulated part of the signature genes were used. This was the case for ER and HER2 signatures but not for survival predictive (relapsing status) signature, for which some gene tags had cross the zero point and resided in the region with the opposite correlation sign. These genes, including *COL20A1*, *SCRN1*, and *MRPL20*, represented a subset of concurrent genes which were difficult to detect under conventional supervised, or phenotype correlation-based, gene selection/filtration criteria.

We went a step further to evaluate the distributions of concurrent signature genes when expression data were sorted by their variability instead of the phenotype correlation. A pre-ranked gene list containing the coefficient of variance (CV) of all array elements was used for GSEA (in pre-rank mode). Since gene variability was continuous rather than discrete, there was no need to separate each gene set into the up/down regulated parts. Figure S9A-S9F showed the positions of gene set members on the rank ordered list by CV: the distributions of concurrent signature genes were not necessarily skewed toward the beginning of the ranked list metric (with higher CV). A gene selection/filtration strategy by variability of genes might lose the chance to identify these clinical relevant concurrent genes.

## Discussion

Presently, adjuvant therapy for breast cancer after surgery is based on some well-established prognostic factors, such as IHC results for ER and HER2. These parameters, however, are not sufficiently predictive of all individuals. In order to confront the heterogeneity not accounted for by conventional clinical and pathological factors, screening for potential biomarkers is one of the most urgent tasks of molecular biology and genomic medicine. In the current study we found that breast cancer was heterogeneous in both gene expression and CNV. Breast cancer CNV was detected by array CGH and potential targets of cancer therapy were identified by GISTIC. Furthermore, genes with coherent CNV and differential gene expression were revealed, and we used these concurrent genes as filtering criteria to derive gene signatures associated with clinical ER and HER2 phenotype. Distributions of these ER- and HER2- associated genes showed strong chromosome dependency, indicating the necessity of identifying concurrent genes. Finally, a breast cancer risk prediction model was built based on a 16-gene signature, and distinct survival patterns were observed for the high- and low-risk groups. The prognostic value of proposed concurrent genes was conserved across distinct clinical ER and HER2 statuses, which were ascertained in subgroup analyses.

Chromosomal aberrations are prognostic themselves and may cause alternations in gene expression. For instance, Zudaire et al. used metaphase chromosome CGH to evaluate genomic aberrations associated with breast cancer and found that 16q loss was associated with a better prognosis while 1q, 11q, 17q, and 20q gains were associated with poorer prognoses [38]. Nessling et al. used array CGH from 31 breast cancers with lymph node metastasis and identified 37 gains and 13 losses from 112 candidate genes [39]. Previous studies dealing directly with the correlation between CNV and gene expression in breast cancer include Bergamaschi et al., in which array CGH results were analyzed for 89 locally advanced breast cancers and correlated with gene expression profiles used for molecular subtyping as defined by Stanford/UNC intrinsic signatures [4,20]. The main drawback of the aforementioned studies was that gene expression data were not from the same subjects assayed for CNV [18,40,41].

The merit of the current study is that both array CGH and gene expression were performed on the same breast cancer patients, and hence concurrent gains and losses could be identified in an unbiased manner. It should also be noted that for dual-color array CGH, we used matched cancerous and normal breast tissue for hybridization to eliminate inter-individual variability [42]. Our use of matched normal breast tissue, rather than pooled genomic DNA, as the common reference greatly enhanced the reliability of CNVs detected at the expense of false negativity, i.e. loss of information. As “physiological” or non-diseased CNV may account for up to 12% of human genomic DNA, our matching strategy resulted in lower frequency of CNV detected than other studies [43]. This was evident from the much lower and flattened peaks shown in Figure 2.

With the advent of high-throughput microarray technology, more than hundreds of thousands of genes can be measured in a single hybridization, and gene selection/filtration is a must; the most sophisticated bioinformatics tools have been applied to cope with the numerous complicated and correlated gene expression data [44]. There is no gold standard regarding the process of gene selection or filtration; nevertheless, there is no doubt that a priori selection will cause bias in the discovery of biomarkers. One of the strengths of our study design is the circumvention of traditional a priori gene selection through the identification of concurrent genes, thus reducing bias and increasing accuracy. The gene-focused content of the array CGH facilitated the comparison of CGH and gene expression data so that we could correlate genomic CNV with gene expression alterations (with the aid of the CGH gene-centric table). Through the parallel utilization with array CGH and gene expression analysis, these technologies provide a platform for facilitating our understanding of genetic complexity inherited in endemic breast cancer and enhancing the search for novel biomarkers.

The rationale and significance of using concurrent genes as the pre-selection criteria was justified for the following reasons. The identification of concurrent genes provided an alternative approach for gene discovery. In contrast to more commonly used gene selection/filtration criteria, we could find several biomarkers which were not readily identified from gene expression only data, and their prognostic values were validated in the combined dataset with independent samples. Traditionally genes were selected/filtered based on phenotype correlation, or their variability. In current study the purposed signatures (gene sets) were significantly enriched when microarray data were sorted by the corresponding phenotype, providing additional validations of the concurrent genes. Besides, the concurrent signature genes were uncovered from the ranked list metric, and if some of them were not at the top/bottom of the rank list (sorted by phenotype correlation) or at the high variability end (pre-ranked analysis, weighted by coefficient of variance), these candidate genes might be neglected easily when only gene expression data was available without a knowledge of the interplay between genomic and gene expression profiles.

Indeed, not all biomarkers will show concurrent hierarchy between genomic and transcriptional aberrations. This may result from non-linear transcription such as alternative splicing, post-transcriptional modifications, or just the time lag between successive steps of the central dogma. However, the merit of current study was to identify those concurrent markers not recognizable by conventional gene expression only approach. When comparing genes from published breast cancer signatures with the concurrent genes, 3 (*AURKA*, *ERBB2*, and *GRB7*) from the Oncotype DX^TM^ signature, 5 from the Rotterdam signature (*ACACB*, *ACOT11*, *CD44*, *TNFSF13*, and *UCKL1*) and 20 (*AK2*, *AURKA*, *BAG4*, *CSE1L*, *ERBB2*, *ETFA*, *FUBP1*, *GALE*, *GATM*, *GPSM2*, *GRB7*, *IDH2*, *ISG15*, *LRP8*, *NDUFB5*, *POLB*, *PTPRF*, *RCAN3*, *SLC9A3R* , and *STK24*) from the Hu306 intrinsic signature were among the 629 concurrent genes in current study, highlighting the necessity of concurrent gene filtration in biomarker discovery and prognostic prediction.

Initially, we used a univariate test and 10^-3^ significance level to identify genes differentially expressed between distinct clinical phenotypes. For the ER signature, the most differentially expressed gene, *ADCY9*, was reported in the calcium and gonadotropin releasing hormone signaling pathways. Genes reported in Perou’s breast cancer intrinsic genes list, including *WWP1*, *TECA3*, and *ADRM1*, were also differentially expressed between ER-positive and -negative breast cancers [2-4]. The second most differentially expressed gene, *NME3*, is associated with purine and pyrimidine metabolism as well as tumor metastasis [45]. *HAGH* relates to pyruvate metabolism and immunology. *IKBKB* is involved in NF-κB, TNFR2, MAPK, and insulin signaling pathways, as well as in apoptosis [46,47]. For the HER2 signature, five genes including *STARD3*, *ERBB2*, *GRB7*, and *MED24* were reported in Perou’s intrinsic genes [2-4]. *STARD3* and *GRB7* were also recognized as differentially expressed genes in the ER-positive and -negative breast cancers in our 81 samples, indicating some interactions between the ER and HER2 pathways [48]. The benefit of using of consensus genes for ER and HER2 signatures was supported from Table S4A and S4B; each classification method performed quite well in original training samples, but compromised results were observed from independent validation data. Model over-fitting was speculated and we adopted the consensus genes to synthesize the final signatures with parsimony and generalizability with fewer genes and higher overall predictive accuracy. Discrepancy among multiple classification methods was reduced as well.

Our study identified subsets of the concurrent genes associated with breast cancer recurrence, metastases, or mortality in survival analyses. A 16-gene signature (*RCAN3*, *MCOLN2*, *DENND2D*, *RWDD3*, *ZMYM6*, *CAPZA1*, *GPR18*, *WARS2*, *TRIM45*, *SCRN1*, *CSNK1E*, *HBXIP*, *CSDE1*, *MRPL20*, *IKZF1*, and *COL20A1*) was established for disease-free survival. Insight into the roles of these genes in breast cancer can be gleaned from their known roles in other cancers and in normal cellular physiology. *DENND2D* was shown to be down-regulated in non-small cell lung cancer and might act as a tumor-suppressor gene [49], and suppressed expression was observed for patients with relapses in our study. *TRIM45* suppresses cell proliferation as a repressor of the NF-κB signal pathway [50], and down-regulation was also observed in the relapsing Han Chinese patients in this study. *SCRN1* was reported in Perou’s breast cancer intrinsic genes list and was speculated to be a prognostic marker in colorectal cancer [2-4,51]. *CSNK1E* encodes a beta-catenin destruction complex and is involved in aberrations of the Wnt/beta-catenin signaling pathway, which lead to beta-catenin oncoprotein accumulation in the nucleus [52,53]; elevated expression in breast cancer patients with relapses was observed in the current study (Figure S4). *HBXIP* is known to promote the migration and proliferation of breast cancer cells [54,55].

The combined 408 Han Chinese breast cancers were also subtyped by intrinsic genes developed by the Stanford/UNC group for prognostic comparisons. By definition, intrinsic genes are those that show the highest variation across different subjects and show the least variation within each individual (i.e. pre-/post-chemotherapy changes). Our previous work has ascertained the clinical applicability of intrinsic taxonomy for breast cancers in patients of Han Chinese origin, and we used the 306 intrinsic genes suggested by Hu et al. and mean-centering of genes to account for systematic bias across microarray studies, as well as centroid-based single sample prediction for molecular subtyping [5,33]. After removing cases designated as normal breast-like and unclassified categories, 340 samples were suitable for comparison. As shown in Figures 4B, 5B, and 6B as well as in Figures S5B and S6B, our risk prediction model based on 16 concurrent genes was capable of separating the high- and low-risk groups according to actual disease-free survival for up to 12 years, whether the whole study cohort or subgroups of distinct clinical ER and HER2 status were analyzed. In contrast, the prognostic value of intrinsic subtypes was less clear for ER-negative tumors due to the poor separation of basal-like and HER2-enriched subtypes (Figure 6A). For HER2 overexpressing breast cancers, the basal-like subtype was associated with the worst outcome, whereas the prognoses of HER2-enriched subtype and luminal B subtype were intertwined, and molecular profiling provided little prognostic value (Figure S5A).

We also compared the prognostic values of concurrent genes with genes reported from the Amsterdam, Rotterdam, and Oncotype DX^TM^ signatures, all were well-known signatures and were validated intensively in the past decade. However, molecular subtyping by these signatures was far beyond the scope of current study, and as an alternative, we constructed supervised principle component regression from these signature genes, as we did for concurrent genes, to have a comparable prognostic benchmarking. Risk prediction by concurrent genes provided the best discriminative power with the highest AUC value, and remained valid through sensitivity analysis. It should be noticed that MammaPrint® (Agendia, Irvine, CA), the commercial version of the Amsterdam 70-gene signature, was indicated for early stage breast cancers (stage I and II), less than 61 years old, and without regional lymph node metastasis. The Rotterdam 76-gene signature, not yet commercially available, was intended for lymph node negative breast cancers. The risk assessment by Oncotype DX^TM^ (Genomic Health, Redwood City, CA) might benefit stage I/II, ER positive/nodal negative breast cancers planned for adjuvant hormone/chemo-therapy. All three tests had their intended breast cancer subpopulations, which in part explained their compromised performance in current study. Other biases arose from predictions by supervised principle component rather than original published algorithms, as well as ethnic discrepancy in study populations.

Our study had several limitations. First, the definition of ER positivity and HER2 overexpression was somewhat different between our series and the 125 Chinese breast cancers (GSE5460, Lu et al. dataset), which hampered the comparability between these two independent studies. Second, the follow-up time was much shorter for our 81 breast cancer patients than those of Kao et al., and more than four-fifths of our patients were still disease-free, while nearly 30% of their patients had metastasis or mortality. This discrepancy could also result from demography, cancer characteristics, or treatment divergence, but unequal follow-up time inevitably compromised the comparability between these two cohorts. Third, clinical ER and HER2 status was not available for the 327 breast cancers from Kao et al., and their ER and HER2 phenotypes could only be deduced from the corresponding probe sets.

Breast cancer phenotypes may correlate with gene expression, protein levels, or non-coding transcription. In this study, the focus was on copy number changes that resulted in changes in gene expression, and several novel biomarkers were discovered. Other mechanisms of transcriptional regulation were not addressed. For instance, *ERBB2* was among the 629 concurrent genes, indicating a dose-effect on the HER2 signaling pathway through transcription, whereas *ESR1* did not display such coherent regularity, and a more complex modulation of post-transcriptional modifications might be postulated for the ER pathway.

## Conclusion

In the current study, we developed an analytical approach to search for genes with concurrent patterns between gene expression and CNV, and signatures for ER, HER2, and disease-free survival were constructed using these concurrent genes. Using genomic as well as transcriptional data from parallel analyses of array CGH and gene expression microarray from the same individual, we increase the confidence level of our gene identification by reducing false discoveries in finding breast cancer biomarkers. Chromosomal aberrations appeared to play a major role in regulating transcription. We anticipate that the results of this study will facilitate the development of screening methods for breast cancer biomarker discovery as more prospective samples become available.

## Supporting Information

Figure S1 **Frequencies of copy number variation and positively correlated genes across the genome.**
(TIF)

Figure S2 **Clustering of the 36 ER signature genes with average-linkage and 1-correlation metric.** X-axis: yellow indicating ER-positive and blue indicating ER-negative samples. Y-axis: signature genes. Heat map color scale: orange indicating up-regulated and blue indicating down-regulated genes.
(TIF)

Figure S3 **Clustering of the 9 HER2 signature genes with average-linkage and 1-correlation metric.** X-axis: yellow indicating HER2 overexpressing and blue indicating normal HER2 samples. Y-axis: signature genes. Heat map color scale: orange indicating up-regulated and blue indicating down-regulated genes.
(TIF)

Figure S4 **Clustering of the 16 risk prediction model genes with average-linkage and 1-correlation metric.** X-axis: yellow indicating cases with relapses and blue indicating cases remaining diseases-free. Y-axis: signature genes. Heat map color scale: orange indicating up-regulated and blue indicating down-regulated genes.
(TIF)

Figure S5 **Disease-free survival in 95 HER2 overexpressing Han Chinese breast cancers: (A) stratified by intrinsic subtypes (Log-rank test: *P* = 0.08), and (B) stratified by the 16-concurrent gene signature (Log-rank test: *P* < 0.001).** SSP: single sample prediction, Her2: HER2-enriched, LumA: luminal A, LumB: luminal B subtype breast cancer. X-axis: survival time in years.
(TIF)

Figure S6 **Disease-free survival in 245 Han Chinese breast cancers with normal HER2 status: (A) stratified by intrinsic subtypes (proportional hazards assumption violated), and (B) stratified by the 16-concurrent gene signature (Log-rank test: *P* = 0.02).** SSP: single sample prediction, Her2: HER2-enriched, LumA: luminal A, LumB: luminal B subtype breast cancer. X-axis: survival time in years.
(TIF)

Figure S7 **Receiver operating characteristics (ROC) analysis for supervised principle component risk predictive models.** Predictive models were based of (A) Amsterdam signature genes, (B) Rotterdam signature genes, (C) Oncotype DX^TM^ signature genes, and (D) concurrent genes (AUC: area under the curve).
(TIF)

Figure S8 **Plots of gene set enrichment analysis (GSEA).** Enrichment in ER positive phenotype by the gene set of up-regulated in ER signature genes in 81 Taiwanese breast cancers (A) and Lu et al. data (B). Enrichment in ER negative phenotype by gene set of down-regulated in ER signature genes in 81 Taiwanese breast cancers (C) and Lu et al. data (D). Enrichment in HER2 overexpressing phenotype by the gene set of up-regulated in HER2 signature genes in 81 Taiwanese breast cancers (E) and Lu et al. data (F). Enrichment in relapsing cancers by the gene set of up-regulated in survival predictive (relapsing status) signature genes in 81 Taiwanese breast cancers (G) and Kao et al. data (H). Enrichment in disease-free cancers by the gene set of down-regulated in survival predictive (relapsing status) signature genes in 81 Taiwanese breast cancers (I) and Kao et al. data (J).
(TIF)

Figure S9 **Plots of pre-ranked analysis weighted by individual genes’ coefficient of variance.** Enrichment by the ER signature gene set in 81 Taiwanese breast cancers (A) and Lu et al. data (B). Enrichment by the HER2 signature gene set in 81 Taiwanese breast cancers (C) and Lu et al. data (D). Enrichment by the survival predictive (relapsing status) signature in 81 Taiwanese breast cancers (E) and Kao et al. data (F).
(TIF)

Methods S1 A. Molecular subtyping by intrinsic genes.
B. Determining clinical ER and HER2 status from gene expression data.
C. Inference of concurrent signature genes across microarray studies.
D. Algorithms in microarray class prediction.
E. Breast cancer risk predictive model based on genes from Amsterdam, Rotterdam, and Oncotype DX^TM^ signatures.
F. Concurrent gene sets for GSEA.
(DOCX)

Table S1 **GISTIC analysis in a sample of 23 Taiwanese breast cancers: (A) gain, and (B) loss regions.**
(DOCX)

Table S2 **Complete list of the 629 concurrent genes.**
(DOCX)

Table S3 **Molecular subtyping of the 408 Taiwanese breast cancers with the Stanford/UNC intrinsic genes: (A) molecular subtyping of the 81 Taiwanese breast cancers from the current study, (B) molecular subtyping of the 327 Taiwanese breast cancers from Kao et al., and (C) molecular subtyping of the combined dataset of 408 Taiwanese breast cancers.**
(DOCX)

Table S4 **Predictive of ER and HER2 signatures in training and independent data: (A) predictive accuracy of ER signature, and (B) predictive accuracy of HER2 signature.**
(DOCX)
