# Supplementary figures and images for: Concurrent Gene Signatures for Han Chinese Breast Cancers
Source: PLoS One. 2013 Oct 3;8(10):e76421. doi: 10.1371/journal.pone.0076421 (PMC3789693; doi:10.1371/journal.pone.0076421)

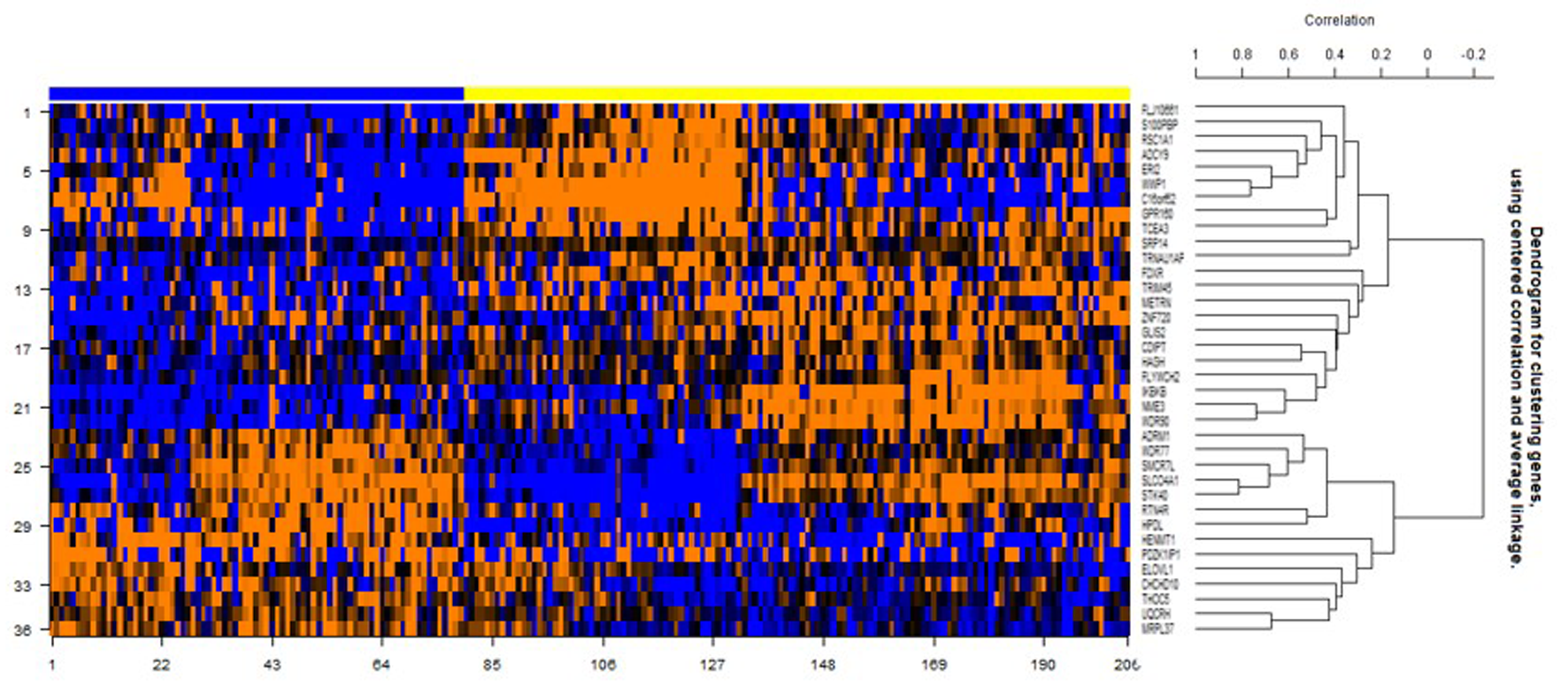

Supplement: Figure S2 — Clustering of the 36 ER signature genes with average-linkage and 1-correlation metric. X-axis: yellow indicating ER-positive and blue indicating ER-negative samples. Y-axis: signature genes. Heat map color scale: orange indicating up-regulated and blue indicating down-regulated genes. (TIF) [file pone.0076421.s002.tif]

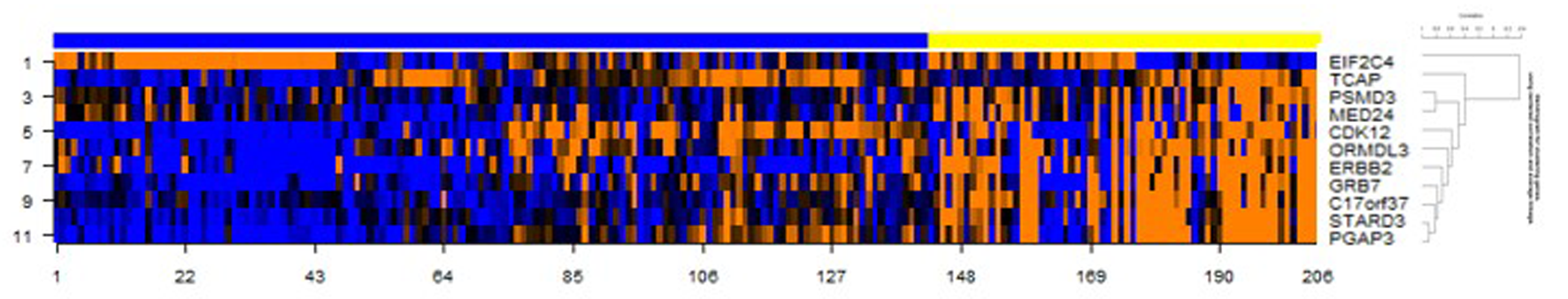

Supplement: Figure S3 — Clustering of the 9 HER2 signature genes with average-linkage and 1-correlation metric. X-axis: yellow indicating HER2 overexpressing and blue indicating normal HER2 samples. Y-axis: signature genes. Heat map color scale: orange indicating up-regulated and blue indicating down-regulated genes. (TIF) [file pone.0076421.s003.tif]

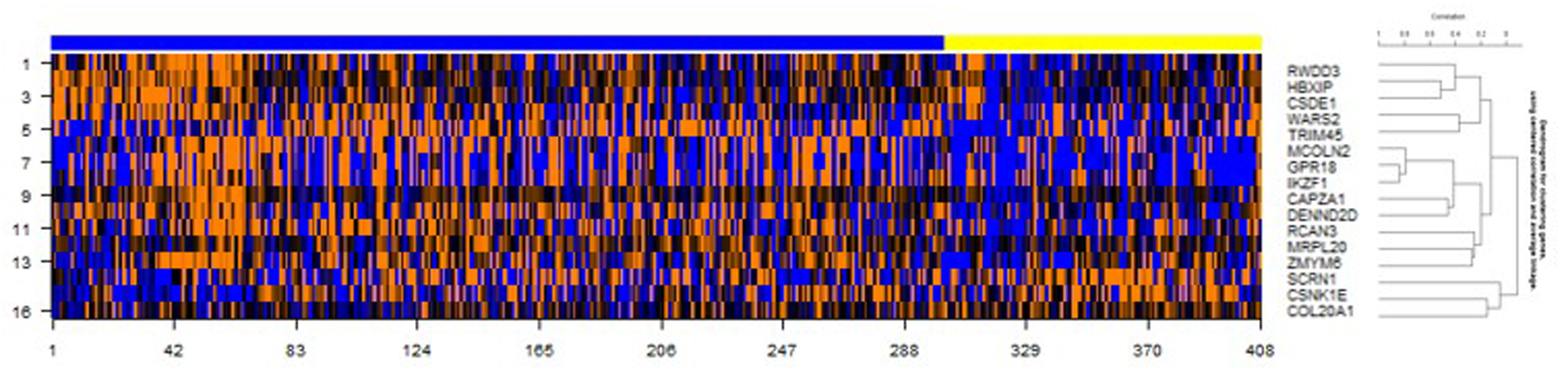

Supplement: Figure S4 — Clustering of the 16 risk prediction model genes with average-linkage and 1-correlation metric. X-axis: yellow indicating cases with relapses and blue indicating cases remaining diseases-free. Y-axis: signature genes. Heat map color scale: orange indicating up-regulated and blue indicating down-regulated genes. (TIF) [file pone.0076421.s004.tif]

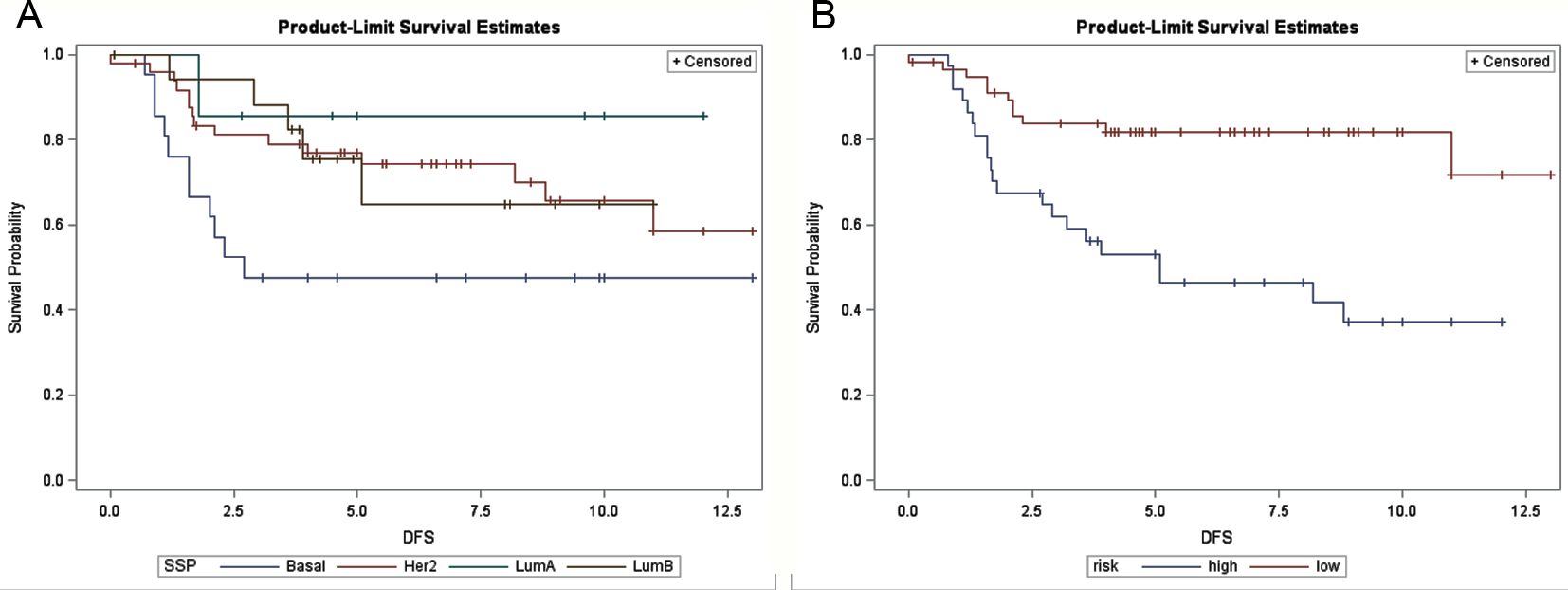

Supplement: Figure S5 — Disease-free survival in 95 HER2 overexpressing Han Chinese breast cancers: (A) stratified by intrinsic subtypes (Log-rank test: P = 0.08), and (B) stratified by the 16-concurrent gene signature (Log-rank test: P < 0.001). SSP: single sample prediction, Her2: HER2-enriched, LumA: luminal A, LumB: luminal B subtype breast cancer. X-axis: survival time in years. (TIF) [file pone.0076421.s005.tif]

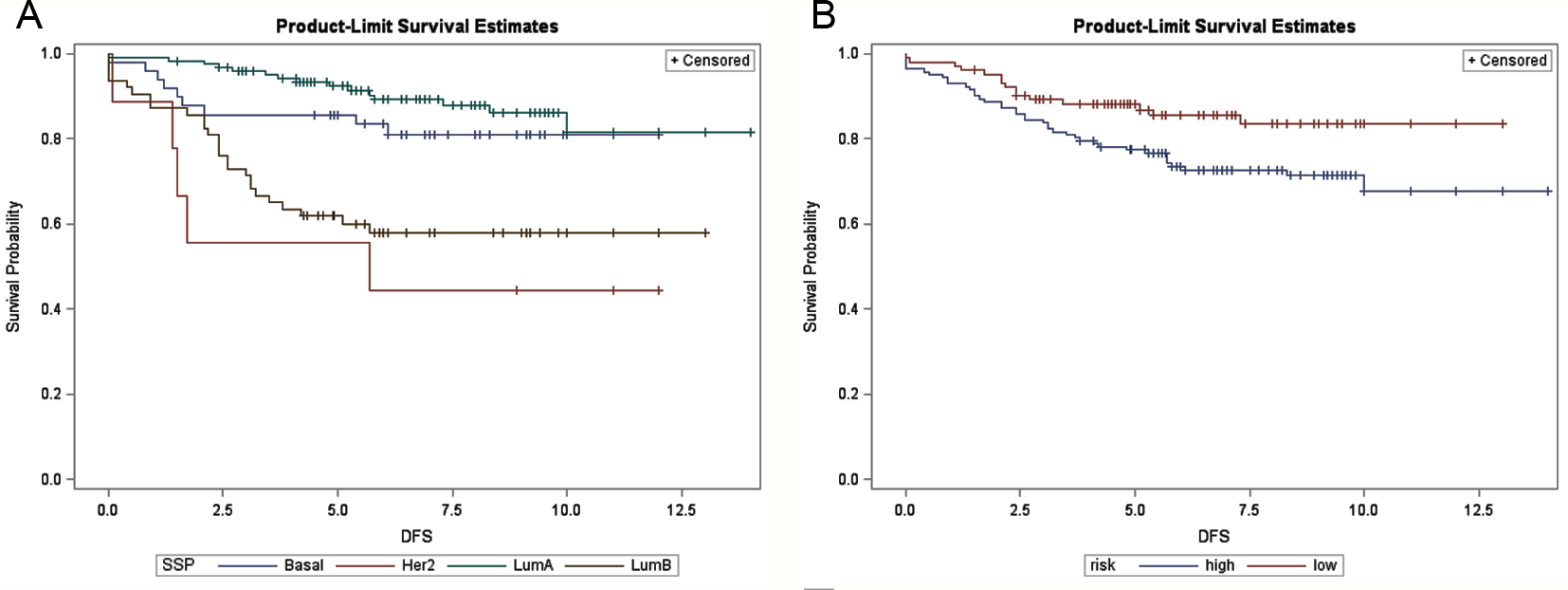

Supplement: Figure S6 — Disease-free survival in 245 Han Chinese breast cancers with normal HER2 status: (A) stratified by intrinsic subtypes (proportional hazards assumption violated), and (B) stratified by the 16-concurrent gene signature (Log-rank test: P = 0.02). SSP: single sample prediction, Her2: HER2-enriched, LumA: luminal A, LumB: luminal B subtype breast cancer. X-axis: survival time in years. (TIF) [file pone.0076421.s006.tif]

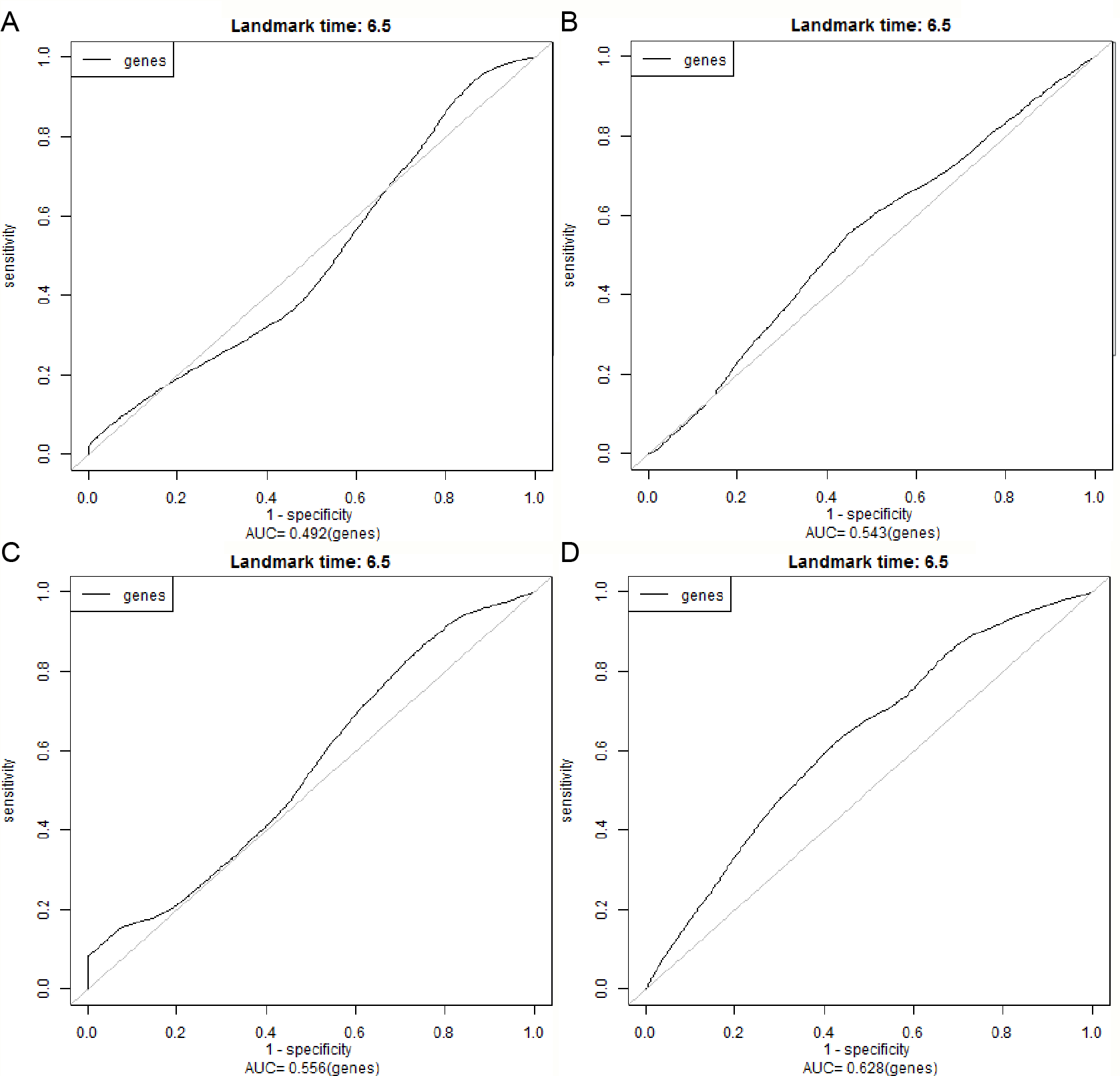

Supplement: Figure S7 — Receiver operating characteristics (ROC) analysis for supervised principle component risk predictive models. Predictive models were based of (A) Amsterdam signature genes, (B) Rotterdam signature genes, (C) Oncotype DXTM signature genes, and (D) concurrent genes (AUC: area under the curve). (TIF) [file pone.0076421.s007.tif]

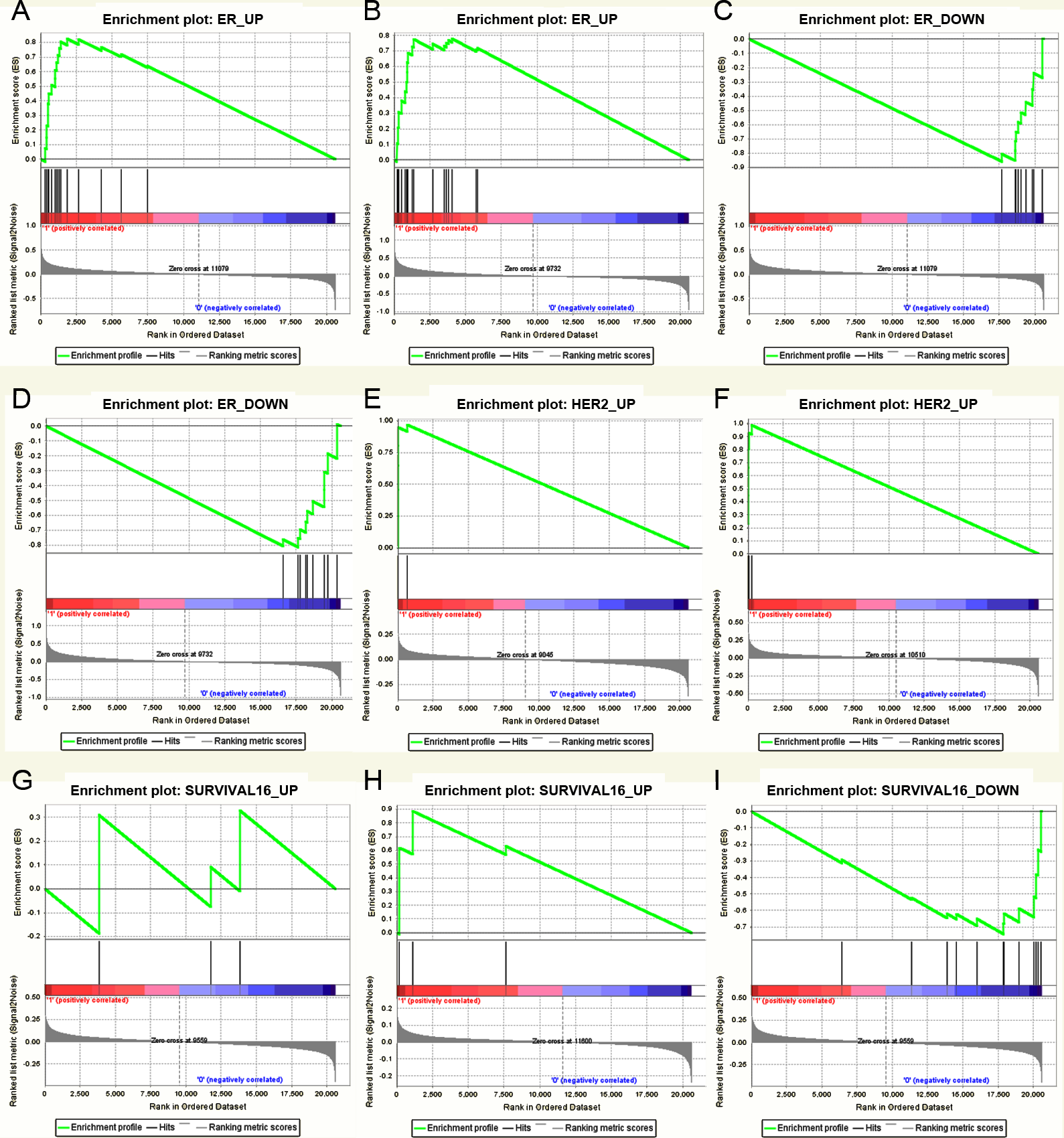

Supplement: Figure S8 — Plots of gene set enrichment analysis (GSEA). Enrichment in ER positive phenotype by the gene set of up-regulated in ER signature genes in 81 Taiwanese breast cancers (A) and Lu et al. data (B). Enrichment in ER negative phenotype by gene set of down-regulated in ER signature genes in 81 Taiwanese breast cancers (C) and Lu et al. data (D). Enrichment in HER2 overexpressing phenotype by the gene set of up-regulated in HER2 signature genes in 81 Taiwanese breast cancers (E) and Lu et al. data (F). Enrichment in relapsing cancers by the gene set of up-regulated in survival predictive (relapsing status) signature genes in 81 Taiwanese breast cancers (G) and Kao et al. data (H). Enrichment in disease-free cancers by the gene set of down-regulated in survival predictive (relapsing status) signature genes in 81 Taiwanese breast cancers (I) and Kao et al. data (J). (TIF) [file pone.0076421.s008.tif]

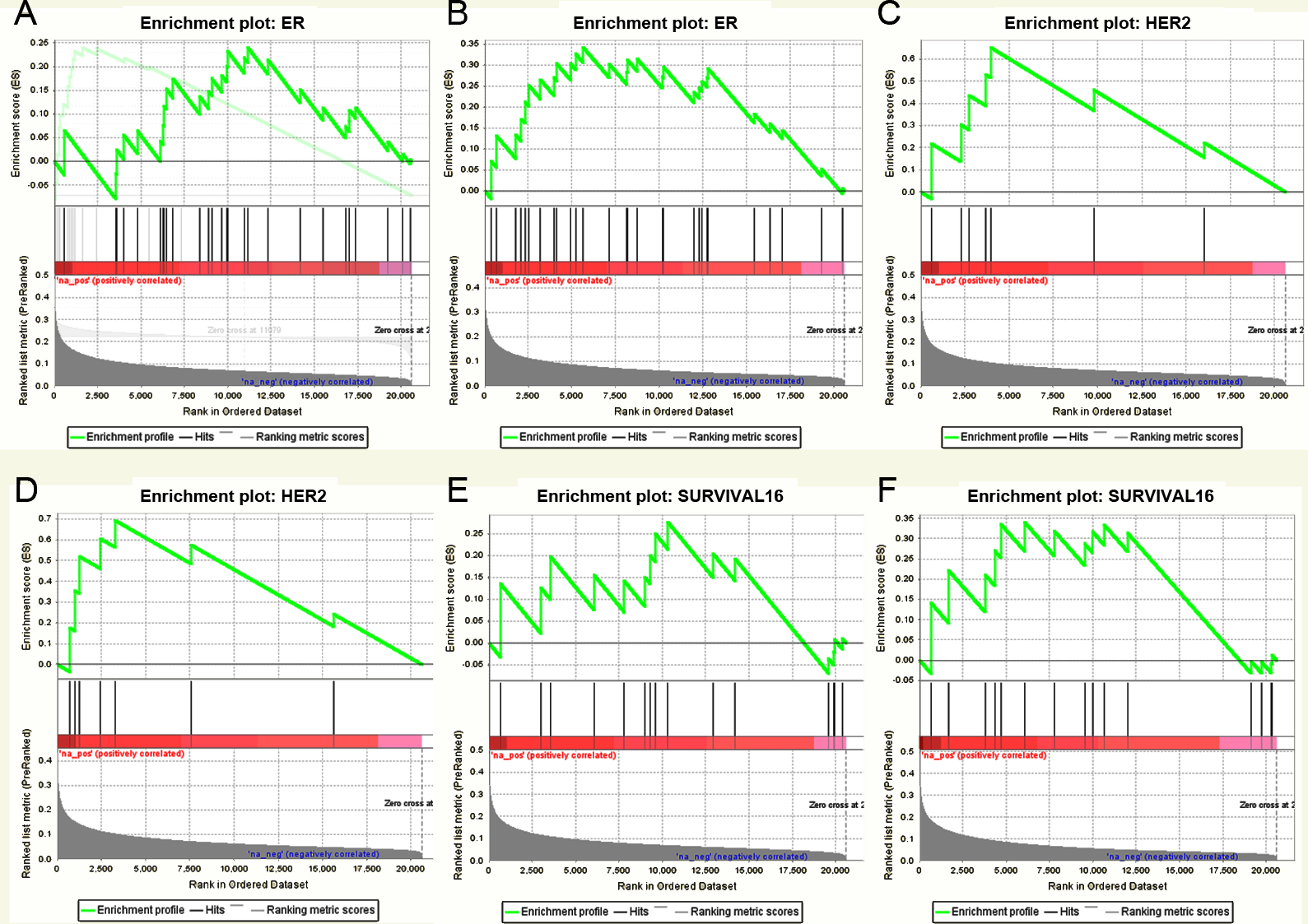

Supplement: Figure S9 — Plots of pre-ranked analysis weighted by individual genes’ coefficient of variance. Enrichment by the ER signature gene set in 81 Taiwanese breast cancers (A) and Lu et al. data (B). Enrichment by the HER2 signature gene set in 81 Taiwanese breast cancers (C) and Lu et al. data (D). Enrichment by the survival predictive (relapsing status) signature in 81 Taiwanese breast cancers (E) and Kao et al. data (F). (TIF) [file pone.0076421.s009.tif]
