# Supplementary material for: Concurrent Gene Signatures for Han Chinese Breast Cancers
Source: PLoS One. 2013 Oct 3;8(10):e76421. doi: 10.1371/journal.pone.0076421 (PMC3789693; doi:10.1371/journal.pone.0076421)
Supplement: Methods S1 — A. Molecular subtyping by intrinsic genes. B. Determining clinical ER and HER2 status from gene expression data. C. Inference of concurrent signature genes across microarray studies. D. Algorithms in microarray class prediction. E. Breast cancer risk predictive model based on genes from Amsterdam, Rotterdam, and Oncotype DXTM signatures. F. Concurrent gene sets for GSEA. (DOCX) [file pone.0076421.s010.docx]

**Supplemental Methods**

**A. Molecular subtyping by intrinsic genes**

We used the 306 intrinsic genes proposed by Hu et al. [5] to define the 5 molecular subtypes (luminal A, luminal B, normal breast-like, HER2-enriched, and basal-like). Centroids were the mean expression values of intrinsic genes corresponding to each molecular subtype. The Hu 306 intrinsic gene lists and prototypical arrays were obtained from UNC Microarray Database. 306 Agilent probeset identifiers were mapped to the latest HUGO gene symbols, then to the Affymetrix gene annotation file. This resulted in 783 Affymetrix probesets representing 300 genes. The prototypical arrays included 46 luminal B, 136 luminal A, 19 HER2-enriched, 65 basal-like and 29 normal breast-like tumors.

The 408 Han Chinese breast cancers were assigned to 1 of the 5 molecular subtypes with the nearest centroid method (single sample prediction). Spearman correlation coefficients were used, and samples were designated as unclassified if all the correlation coefficients to the 5 centroids were less than 0.1.

To enhance the comparability between the original studies deriving intrinsic genes and independent samples in the current study, mean-centering of genes was applied to the expression data of Han Chinese breast cancers, as suggested by the investigators of the Stanford group.

**B. Determining clinical ER and HER2 status from gene expression data**

We modified the method of Karn et al. [37] and fitted two components (homogeneous) finite mixture model for the speculations of clinical ER and HER2 status. ER was represented by Affymetrix probe set 205225_at (ESR1) and HER2 by 216836_s_at (ERBB2). The location and scale parameters of the two Gaussian distributions were estimated as well as the mixing probability of the two components using the maximum likelihood algorithm. The mixing probability was 0.4038 for ER and 0.7672 for HER2, indicating that approximately 40% and 77% of the 327 breast cancers from Kao et al. were ER-negative and were with normal HER2 expression. The intersection of the two Gaussian distributions represented the threshold determining clinical ER and HER2 status for each sample. The threshold was 10.56 for ER and 12.02 for HER2 and was depicted as the density plot below.

1. Density plot for ESR1 (205225_at)


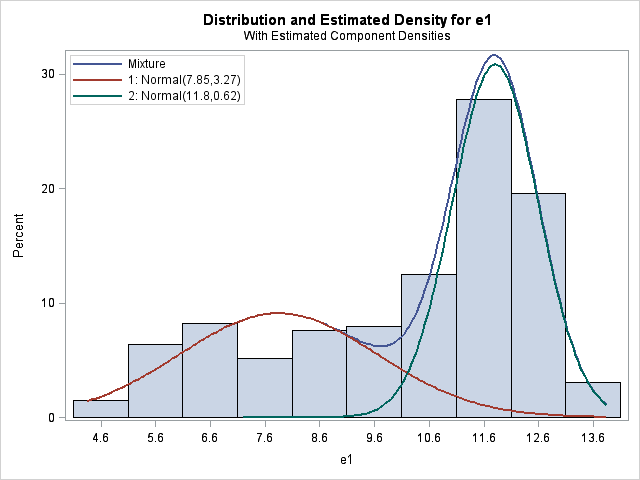


1. Density plot for ERBB2 (216836_s_at)


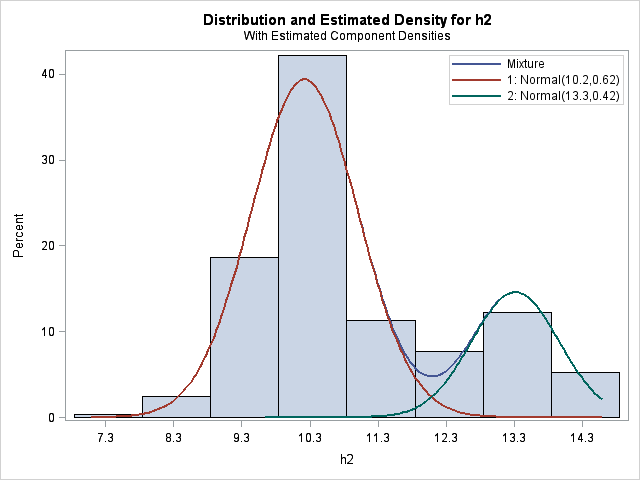


**C. Inference of concurrent signature genes across microarray studies**

1. **ER-signature**

Signatures for ER were identified independently from our 81 Taiwanese breast cancers and 125 Chinese breast cancers (Lu et al. dataset, GSE5460) with clinical IHC results as gold standard. Genes differentially expressed between ER positive and negative tumors were selected by two-sample t-test with random variance (α level: 0.001), and the derived ER classifiers with multiple methods were tested on independent samples not used for training as external validation.

Forty-nine genes were selected from 81 Taiwanese breast cancers, and a relative higher proportion of ER-signature genes from chromosome 16 was observed. These 49 ER-signature genes were used to construct classifiers with multiple methods. Predictive accuracy between 80%~84% during leave-one-out cross-validation was observed (Supplemental Table 4A). The predictive accuracy dropped to 67%~83% when trained classifiers were tested in independent microarray experiments of 125 Chinese breast cancers (Lu et al. dataset, GSE5460). The best predictive method was 3-nearest neighbors for both training and independent test dataset.

ER signature was identified independently from 125 Chinese breast samples (Lu et al. dataset, GSE5460) with the same significance level of 0.001 and two-sample t-test, and 252 genes were filtered. Accuracy during leave-one-out cross-validation was between 90~95%, and predictive performance was compromised when our 81 breast cancers were used as external validation (accuracy: 62~81%, Supplemental Table 4A). Again the best predictive method was 3-nearest neighbors for both training and independent test dataset.

1. **HER2-signature**

Using the same univariate two-sample t-test with the significance level of 10^-3^, genes differentially expressed between HER2 overexpressing tumors and those with normal HER2 status were identified from our series and 125 Chinese breast cancers (Lu et al. dataset, GSE5460). For 81 Taiwanese breast cancers, there were 16 genes identified, and many of which were located in chromosome 17. The predictive accuracy of HER2-signature genes with multiple methods was between 68% and 78% during leave-one-out cross-validation. When tested in independent dataset of 125 Chinese breast cancers (Lu et al. dataset, GSE5460), the predictive accuracy was only 34~66% (Supplemental Table 4B).

HER2-signature was derived independently from 125 Chinese breast samples (Lu et al. dataset, GSE5460) with the same selection criteria, and 43 genes were identified. Accuracy during leave-one-out cross-validation was between 90~94%, and predictive performance was somewhat compromised when our 81 breast cancers were used as external validation (accuracy: 63~74%, Supplemental Table 4B). For HER2 signature, 3-nearest neighbors and the nearest centroid classifiers reported the most optimistic predictions; the discrepancy among multiple methods remained high though.

D**. Algorithms in microarray class prediction**

Multiple methods were used when class prediction was performed for gene expression data in current study. These methods, including compound covariate predictor, diagonal linear discrimination, 3-nearest neighbor, nearest centroid, and support vector machine, were supervised in nature since clinical ER and HER2 status was considered as gold standard. Genes were median-centered first to avoid the bias introduced by those with extremely high overall intensities.

The compound covariate predictor used a weighted linear combination of pre-selected genes, which were filtered by a specified univariate t-test. This univariate t-statistic was used as the weighting parameter for the multi-variate predictor, with opposite sign for each class label. The diagonal liner discrimination was a version of linear discriminative function that correlations among genes were omitted to avoid model over-fitting. The 3-nearest neighbor used the voting result of the 3 most similar training samples to predict the class of sample in question. When comparing multi-dimensional gene expression profiles, Euclidean distance was used as distance metric. The nearest centroid algorithm calculated the distance between the test sample and the centroid of corresponding class; the sample was predicted to belong to the class with the shortest distance. The centroid was the mean expression value of all training samples with identical class. The support vector machine found the best hyperplanes to separate data points of distinct class in high-dimensional space with misclassifications penalized. All classifications were performed with class prediction functions of the BRB ArrayTools [24].

**E. Breast cancer risk predictive model based on genes from Amsterdam, Rotterdam, and Oncotype DX^TM^ signatures**

Prognostic comparisons of concurrent genes with signature genes reported for the Amsterdam, Rotterdam, and Oncotype DX^TM^ were performed. Supervised principle component regression from these signature genes was constructed, as we did for concurrent genes, in an effort to have a comparable prognostic benchmarking. Genes reported in each signature were retrieved; for the 70 cDNA clones of the Amsterdam signature, 57 genes were unambiguously mapped to our microarray platform. The 76 Affymetrix U133A probesets reported by the Rotterdam signature, all of which were measured by the U133 plus 2.0 array of the current study, were collapsed to 65 unique genes. The 21 RT-PCR products of the Oncotype DX^TM^ signature, after eliminating five reference genes, were also mapped to the corresponding Affymetrix probesets. The identifications of 70 genes of the Amsterdam signature were determined from Figure 2 of ref. 8. The 76 probesets of the Rotterdam signature were determined from Table 3 of ref. 35. All 16 RT-PCR products of the Oncotype DX^TM^ were identifiable from microarrays of current study. The final contents of each signature after conversion were listed below:

| Amsterdam | Rotterdam | Oncotype DX^TM^ |
| --- | --- | --- |
| AA555029_RC | ABLIM1 | AURKA |
| ALDH4A1 | ACACB | BAG1 |
| AP2B1 | ACOT11 | BCL2 |
| AYTL2 | AP2A2 | BIRC5 |
| BBC3 | ARHGDIB | CCNB1 |
| C16orf61 | ATAD2 | CD68 |
| C20orf46 | BCL2L14 | CTSL2 |
| C9orf30 | BICD1 | ERBB2 |
| CCNE2 | C11orf51 | ESR1 |
| CDC42BPA | C11orf9 | GRB7 |
| CDCA7 | C3 | GSTM1 |
| CENPA | CAPN2 | MKI67 |
| COL4A2 | CBX3 | MMP11 |
| DCK | CCNE2 | MYBL2 |
| DIAPH3 | CD44 | PGR |
| DTL | CEP57 | SCUBE2 |
| EBF4 | CLN8 |  |
| ECT2 | CNKSR1 |  |
| EGLN1 | COL2A1 |  |
| ESM1 | DUSP4 |  |
| EXT1 | EEF1A2 |  |
| FGF18 | ETV2 |  |
| FLT1 | FEN1 |  |
| GMPS | FKBP2 |  |
| GNAZ | FUT3 |  |
| GPR126 | GABRQ |  |
| GPR180 | GAS2 |  |
| GSTM3 | GFOD2 |  |
| HRASLS | GOLM1 |  |
| IGFBP5 | GTSE1 |  |
| JHDM1D | HDGFRP3 |  |
| KNTC2 | HIST1H4H |  |
| LGP2 | IL18 |  |
| LIN9 | KIAA0748 |  |
| LOC100131053 | KPNA2 |  |
| LOC100288906 | LST1 |  |
| LOC730018 | MAP4 |  |
| MCM6 | MLF1IP |  |
| MELK | MYH2 |  |
| MMP9 | NCAPG2 |  |
| MS4A7 | NEFL |  |
| MTDH | NEURL |  |
| NMU | OR12D2 |  |
| NUSAP1 | ORC3 |  |
| ORC6L | PARP4 |  |
| OXCT1 | PHF11 |  |
| PALM2 | PLK1 |  |
| PECI | POLQ |  |
| PITRM1 | PPP1CC |  |
| PRC1 | PSMC2 |  |
| QSCN6L1 | RFX7 |  |
| RAB6B | RPL23AP7 |  |
| RASSF7 | RRNAD1 |  |
| RECQL5 | SLC35A1 |  |
| RFC4 | SMC4 |  |
| RTN4RL1 | SUPT16H |  |
| RUNDC1 | TACC2 |  |
| SCUBE2 | TMEM8A |  |
| SERF1A | TNFSF10 |  |
| SLC2A3 | TNFSF13 |  |
| STK32B | UCKL1 |  |
| TGFB3 | YIF1A |  |
| TSPYL5 | ZCCHC8 |  |
| UCHL5 | ZFP36L2 |  |
| WISP1 | ZNF362 |  |
| ZNF533 |  |  |

**F. Concurrent gene sets for GSEA**

For GSEA parameters, the 54,675 features on the Affymetrix microarray were collapsed to 20606 genes (“Collapse dataset to gene symbols” was true). Gene set size filter was set between 1 and 500 and permutation times was 1,000. The metric for ranking genes for categorical phenotype was the default “Signal2noise”, i.e. signal to noise level.

It was quite often that the pre-selection of genes from all array elements was based on the phenotype correlation, or the variability of each gene. The concurrent signature genes were uncovered from the ranked list metric, and if some of them were not at the top/bottom of the rank list (sorted by phenotype correlation) or at the high variability end (pre-ranked analysis, weighted by coefficient of variance), these candidate genes might be easily ignored when gene expression data was analyzed with a pre-selection of gene step. Depending on GSEA or pre-ranked analysis, concurrent gene sets used were detailed in the following:

| **Gene sets for GSEA** |  |  |  |  |  |  |  |  |  |  |
| --- | --- | --- | --- | --- | --- | --- | --- | --- | --- | --- |
| Up-regulated in ER signature | *WWP1* | *GPR160* | *ADCY9* | *IKBKB* | *WDR90* | *NME3* | *TCEA3* | *METRN* | *ERI2* | TRIM45 |
|  | *GLIS2* | *C16orf52* | *FLJ10661* | *FDXR* | *S100PBP* | *SRP14* | *RSC1A1* | *CDIPT* | *FLYWCH2* | HAGH |
|  | *ZNF720* | *TRNAU1AP* |  |  |  |  |  |  |  |  |
| Down-regulated in ER signature | *STK40* | *SMCR7L* | *ELOVL1* | *THOC5* | *ADRM1* | *WDR77* | *RTN4R* | *SLCO4A1* | *UQCRH* | CHCHD10 |
|  | *MRPL37* | *HPDL* | *HENMT1* | *PDZK1IP1* |  |  |  |  |  |  |
| Up-regulated in HER2 signature | *ERBB2* | *GRB7* | *C17orf37* | *STARD3* | *PGAP3* | *PSMD3* | *ORMDL3* | *MED24* | *CDK12* |  |
| Down-regulated in HER2 signature |  |  |  |  |  |  |  |  |  |  |
| Up-regulated in survival predictive (relapsing status) signature | SCRN1 | CSNK1E | COL20A1 |  |  |  |  |  |  |  |
| Down-regulated in survival predictive (relapsing status) signature | MRPL2 | CAPZA1 | HBXIP | ZMYM6 | CSDE1 | RWDD3 | WARS2 | DENND2D | TRIM45 | RCAN3 |
|  | IKZF1 | MCOLN2 | GPR18 |  |  |  |  |  |  |  |

| **Gene sets for pre-ranked analysis** |  |  |  |  |  |  |  |  |  |  |
| --- | --- | --- | --- | --- | --- | --- | --- | --- | --- | --- |
| ER signature | *WWP1* | *GPR160* | *ADCY9* | *IKBKB* | *WDR90* | *NME3* | *TCEA3* | *METRN* | *ERI2* | *TRIM45* |
|  | *GLIS2* | *C16orf52* | *FLJ10661* | *FDXR* | *S100PBP* | *SRP14* | *RSC1A1* | *CDIPT* | *FLYWCH2* | *HAGH* |
|  | *ZNF720* | *TRNAU1AP* | *STK40* | *SMCR7L* | *ELOVL1* | *THOC5* | *ADRM1* | *WDR77* | *RTN4R* | *SLCO4A1* |
|  | *UQCRH* | *CHCHD10* | *MRPL37* | *HPDL* | *HENMT1* | *PDZK1IP1* |  |  |  |  |
| HER2 signature | *ERBB2* | *GRB7* | *C17orf37* | *STARD3* | *PGAP3* | *PSMD3* | *ORMDL3* | *MED24* | *CDK12* |  |
| Survival predictive (relapsing status) signature | *SCRN1* | *CSNK1E* | *COL20A1* | *MRPL20* | *CAPZA1* | *HBXIP* | *ZMYM6* | *CSDE1* | *RWDD3* | *WARS2* |
|  | *DENND2D* | *TRIM45* | *RCAN3* | *IKZF1* | *MCOLN2* | *GPR18* |  |  |  |  |
