# Supplementary material for: Concurrent Gene Signatures for Han Chinese Breast Cancers
Source: PLoS One. 2013 Oct 3;8(10):e76421. doi: 10.1371/journal.pone.0076421 (PMC3789693; doi:10.1371/journal.pone.0076421)
Supplement: Table S1 — GISTIC analysis in a sample of 23 Taiwanese breast cancers: (A) gain, and (B) loss regions. (DOCX) [file pone.0076421.s011.docx]

**Supplemental Table S1. GISTIC analysis in a sample of 23 Taiwanese breast cancers.**

(A) Gain regions.

|  | **Chromosome** | **Start** | **End** | **Cytoband** | **Number of genes** | **Gene symbol** |
| --- | --- | --- | --- | --- | --- | --- |
| 1 | 1 | 145823199 | 145845247 | 1q21.1 | 1 | GJA8 |
| 2 | 3 | 163997228 | 164038917 | 3q26.1 | 0 |  |
| 3 | 3 | 164893335 | 165015361 | 3q26.1 | 0 |  |
| 4 | 4 | 69085386 | 69114068 | 4q13.2 | 1 | UGT2B17 |
| 5 | 8 | 128302043 | 138943974 | 8q24.21-q24.23 | 34 | POU5F1B, LOC727677, MYC, PVT1, MIR1204, MIR1205, MIR1206, MIR1207, MIR1208, LOC728724, GSDMC, FAM49B, ASAP1, ASAP1-IT, ADCY8, EFR3A, OC90, HHLA1, KCNQ3, HPYR1, LRRC6, TMEM71, PHF20L1, TG, SLA, WISP1, NDRG1, ST3GAL1, ZFAT, ZFAT-AS1, MIR30B, MIR30D, LOC286094, KHDRBS3 |
| 6 | 11 | 129572255 | 129603117 | 11q24.3 | 2 | ST14, ZBTB44 |
| 7 | 17 | 34989300 | 35598980 | 17q12-q21.1 | 24 | NEUROD2, PPP1R1B, STARD3, TCAP, PNMT, PGAP3, ERBB2, C17orf37, GRB7, IKZF3, ZPBP2, GSDMB, ORMDL3, LOC728129, GSDMA, PSMD3, CSF3, MED24, SNORD124, THRA, NR1D1, MSL1, CASC3, RAPGEFL1 |
| 8 | 19 | 46866110 | 46881544 | 19q13.2 | 1 | CEACAM7 |
| 9 | 20 | 2530733 | 2545362 | 20p13 | 1 | TMC2 |
| 10 | 20 | 34783548 | 34817552 | 20q11.23 | 2 | NDRG3, DSN1 |

(B) Loss regions.

|  | **Chromosome** | **Start** | **End** | **Cytoband** | **Number of genes** | **Gene symbol** |
| --- | --- | --- | --- | --- | --- | --- |
| 1 | 2 | 239819855 | 239854639 | 2q37.3 | 1 | HDAC4 |
