# Supplementary material for: Concurrent Gene Signatures for Han Chinese Breast Cancers
Source: PLoS One. 2013 Oct 3;8(10):e76421. doi: 10.1371/journal.pone.0076421 (PMC3789693; doi:10.1371/journal.pone.0076421)
Supplement: Table S2 — Complete list of the 629 concurrent genes. (DOCX) [file pone.0076421.s012.docx]

**Supplemental Table S2. Complete list of the 629 concurrent genes.**

| **Name** | **Accession** | **UG Cluster** | **Symbol** | **Entrez ID** | **Chromosome** | **Cytoband** |
| --- | --- | --- | --- | --- | --- | --- |
| lactamase, beta | NM_171846 | Hs.410388 | LACTB | 114294 | 15 | 15q22.1 |
| rhomboid, veinlet-like 2 (Drosophila) | NM_017821 | Hs.524626 | RHBDL2 | 54933 | 1 | 1p34.3 |
| t-SNARE domain containing 1 | NM_145003 | Hs.370931 | TSNARE1 | 203062 | 8 | 8q24.3 |
| transmembrane channel-like 2 | NM_080751 | Hs.352626 | TMC2 | 117532 | 20 | 20p13 |
| zinc finger protein 572 | NM_152412 | Hs.175350 | ZNF572 | 137209 | 8 | 8q24.13 |
| protein kinase-like protein SgK196 | NM_032237 | Hs.491646 | SGK196 | 84197 | 8 | 8p11.21 |
| microtubule-actin crosslinking factor 1 | NM_033044 | Hs.472475 | MACF1 | 23499 | 1 | 1p32-p31 |
| WD repeat domain 65 | NM_152498 | Hs.527565 | WDR65 | 149465 | 1 | 1p34.2 |
| ATP/GTP binding protein-like 1 | NM_152336 | Hs.679833 | AGBL1 | 123624 | 15 | 15q25.3 |
| PRP38 pre-mRNA processing factor 38 (yeast) domain containing A | NM_032284 | Hs.5301 | PRPF38A | 84950 | 1 | 1p32.3 |
| zinc finger protein 644 | NM_016620 | Hs.173001 | ZNF644 | 84146 | 1 | 1p22.2 |
| leucine rich repeat containing 28 | NM_144598 | Hs.578684 | LRRC28 | 123355 | 15 | 15q26.3 |
| ganglioside induced differentiation associated protein 2 | BC013132 | Hs.310809 | GDAP2 | 54834 | 1 | 1p12 |
| golgin A7 | BC012032 | Hs.654773 | GOLGA7 | 51125 | 8 | 8p11.21 |
| cystatin 9 (testatin) | AF494536 | Hs.698020 | CST9 | 128822 | 20 | 20p11.21 |
| chloride channel Kb | BC020873 | Hs.352243 | CLCNKB | 1188 | 1 | 1p36 |
| zinc finger and SCAN domain containing 20 | BC011404 | Hs.442705 | ZSCAN20 | 7579 | 1 | 1p34.3 |
| N-acetylgalactosaminidase, alpha- | M29276 | Hs.75372 | NAGA | 4668 | 22 | 22q11 |
| ADAM metallopeptidase domain 9 | AF495383 | Hs.591852 | ADAM9 | 8754 | 8 | 8p11.22 |
| leucine rich repeat containing 41 | BC004948 | Hs.144941 | LRRC41 | 10489 | 1 | 1p34.1 |
| hypothetical LOC100130776 | BQ184856 | Hs.656080 | LOC100130776 | 100130776 | 12 | 12q14.1 |
| myelin transcription factor 1 | BC018917 | Hs.279562 | MYT1 | 4661 | 20 | 20q13.33 |
| deoxyribonuclease I | AW468509 | Hs.629638 | DNASE1 | 1773 | 16 | 16p13.3 |
| hypothetical LOC728192 | BC009533 | Hs.559194 | LOC728192 | 728192 | 13 | 13q33.3 |
| nucleolar complex associated 2 homolog (S. cerevisiae) | BC009786 | Hs.405987 | NOC2L | 26155 | 1 | 1p36.33 |
| CD276 molecule | AK074849 | Hs.77873 | CD276 | 80381 | 15 | 15q23-q24 |
| NOP2/Sun domain family, member 4 | BC016907 | Hs.163424 | NSUN4 | 387338 | 1 | 1p34 |
| chibby homolog 1 (Drosophila) | BC008839 | Hs.334911 | CBY1 | 25776 | 22 | 22q12 |
| choline/ethanolamine phosphotransferase 1 | AL833102 | Hs.363572 | CEPT1 | 10390 | 1 | 1p13.3 |
| Rho guanine nucleotide exchange factor (GEF) 7 | AL831814 | Hs.508738 | ARHGEF7 | 8874 | 13 | 13q34 |
| EF-hand calcium binding domain 6 | AK058069 | Hs.658996 | EFCAB6 | 64800 | 22 | 22q13.2 |
| family with sequence similarity 86, member A pseudogene | BC035792 | Hs.656318 | FLJ10661 | 286042 | 8 | 8p23.1 |
| zinc finger and BTB domain containing 8B | AL442095 | Hs.647658 | ZBTB8B | 728116 | 1 | 1p35.1 |
| hypothetical LOC727924 | AK058056 | Hs.525666 | LOC727924 | 727924 | 15 | 15q11.2 |
| HEAT repeat containing 7A | BG028047 | Hs.443139 | HEATR7A | 727957 | 8 | 8q24.3 |
| CD44 molecule (Indian blood group) | W96225 | Hs.502328 | CD44 | 960 | 11 | 11p13 |
| solute carrier family 35, member E4 | BC040191 | Hs.725375 | SLC35E4 | 339665 | 22 | 22q12.2 |
| chromosome 16 open reading frame 72 | BG403486 | Hs.221497 | C16orf72 | 29035 | 16 | 16p13.2 |
| adaptor-related protein complex 3, mu 2 subunit | BG110196 | Hs.654529 | AP3M2 | 10947 | 8 | 8p11.2 |
| hypothetical LOC731779 | BC029463 | Hs.586414 | LOC731779 | 731779 | 8 | 8q24.3 |
| GC-rich promoter binding protein 1-like 1 | CA391618 | Hs.238432 | GPBP1L1 | 60313 | 1 | 1p34.1 |
| Myb-like, SWIRM and MPN domains 1 | BC017579 | Hs.477495 | MYSM1 | 114803 | 1 | 1p32.1 |
| septin 7 pseudogene 2 | BC015774 | Hs.520804 | SEPT7P2 | 641977 | 7 | 7p12.3 |
| TBC1 domain family, member 10A | BC007908 | Hs.655273 | TBC1D10A | 83874 | 22 | 22q12.2 |
| signal recognition particle 14kDa (homologous Alu RNA binding protein) | NM_003134 | Hs.533732 | SRP14 | 6727 | 15 | 15q22 |
| ribosomal protein L19 | NM_000981 | Hs.381061 | RPL19 | 6143 | 17 | 17q11.2-q12 |
| chromosome 22 open reading frame 28 | NM_014306 | Hs.474643 | C22orf28 | 51493 | 22 | 22q12 |
| ATPase, H+ transporting, lysosomal 21kDa, V0 subunit b | BC005876 | Hs.596514 | ATP6V0B | 533 | 1 | 1p32.3 |
| LIM and SH3 protein 1 | NM_006148 | Hs.728766 | LASP1 | 3927 | 17 | 17q11-q21.3 |
| MARCKS-like 1 | NM_023009 | Hs.707012 | MARCKSL1 | 65108 | 1 | 1p35.1 |
| cathepsin A | NM_000308 | Hs.609336 | CTSA | 5476 | 20 | 20q13.1 |
| serine/arginine-rich splicing factor 11 | AU146237 | Hs.479693 | SRSF11 | 9295 | 1 | 1p31 |
| lipopolysaccharide-induced TNF factor | AB034747 | Hs.459940 | LITAF | 9516 | 16 | 16p13.13 |
| osteosarcoma amplified 9, endoplasmic reticulum lectin | NM_006812 | Hs.527861 | OS9 | 10956 | 12 | 12q13 |
| stathmin 1 | NM_005563 | Hs.209983 | STMN1 | 3925 | 1 | 1p36.11 |
| aconitase 2, mitochondrial | NM_001098 | Hs.643610 | ACO2 | 50 | 22 | 22q11.2-q13.31 |
| acidic (leucine-rich) nuclear phosphoprotein 32 family, member A | NM_006305 | Hs.458747 | ANP32A | 8125 | 15 | 15q23 |
| transmembrane 9 superfamily member 2 | NM_004800 | Hs.654824 | TM9SF2 | 9375 | 13 | 13q32.3 |
| lectin, galactoside-binding, soluble, 1 | NM_002305 | Hs.445351 | LGALS1 | 3956 | 22 | 22q13.1 |
| CSE1 chromosome segregation 1-like (yeast) | AF053641 | Hs.90073 | CSE1L | 1434 | 20 | 20q13 |
| polymerase (DNA directed), delta 2, regulatory subunit 50kDa | NM_006230 | Hs.306791 | POLD2 | 5425 | 7 | 7p13 |
| phosphogluconate dehydrogenase | NM_002631 | Hs.464071 | PGD | 5226 | 1 | 1p36.22 |
| histone deacetylase 1 | NM_004964 | Hs.88556 | HDAC1 | 3065 | 1 | 1p34 |
| sterol regulatory element binding transcription factor 2 | NM_004599 | Hs.443258 | SREBF2 | 6721 | 22 | 22q13 |
| solute carrier family 2 (facilitated glucose transporter), member 1 | NM_006516 | Hs.473721 | SLC2A1 | 6513 | 1 | 1p34.2 |
| CDP-diacylglycerol--inositol 3-phosphatidyltransferase | NM_006319 | Hs.121549 | CDIPT | 10423 | 16 | 16p11.2 |
| adhesion regulating molecule 1 | NM_007002 | Hs.90107 | ADRM1 | 11047 | 20 | 20q13.33 |
| EBNA1 binding protein 2 | NM_006824 | Hs.346868 | EBNA1BP2 | 10969 | 1 | 1p35-p33 |
| solute carrier family 9 (sodium/hydrogen exchanger), member 3 regulator 1 | NM_004252 | Hs.724482 | SLC9A3R1 | 9368 | 17 | 17q25.1 |
| ornithine decarboxylase antizyme 2 | AF242521 | Hs.74563 | OAZ2 | 4947 | 15 | 15q22.31 |
| proteasome (prosome, macropain) 26S subunit, non-ATPase, 3 | NM_002809 | Hs.12970 | PSMD3 | 5709 | 17 | 17q21.1 |
| translocation associated membrane protein 1 | NM_014294 | Hs.491988 | TRAM1 | 23471 | 8 | 8q13.3 |
| proteasome (prosome, macropain) subunit, beta type, 3 | NM_002795 | Hs.82793 | PSMB3 | 5691 | 17 | 17q12 |
| WD repeat domain 77 | BF975273 | Hs.204773 | WDR77 | 79084 | 1 | 1p13.2 |
| secernin 1 | NM_014766 | Hs.520740 | SCRN1 | 9805 | 7 | 7p14.3-p14.1 |
| RAE1 RNA export 1 homolog (S. pombe) | NM_003610 | Hs.371698 | RAE1 | 8480 | 20 | 20q13.31 |
| sorting and assembly machinery component 50 homolog (S. cerevisiae) | NM_015380 | Hs.505824 | SAMM50 | 25813 | 22 | 22q13.31 |
| PWP1 homolog (S. cerevisiae) | AI694451 | Hs.506652 | PWP1 | 11137 | 12 | 12q23.3 |
| protein kinase C and casein kinase substrate in neurons 2 | NM_007229 | Hs.162877 | PACSIN2 | 11252 | 22 | 22q13.2-q13.33 |
| COP9 constitutive photomorphogenic homolog subunit 5 (Arabidopsis) | NM_006837 | Hs.491912 | COPS5 | 10987 | 8 | 8q13.1 |
| tumor protein D52 | NM_005079 | Hs.368433 | TPD52 | 7163 | 8 | 8q21 |
| antizyme inhibitor 1 | NM_015878 | Hs.459106 | AZIN1 | 51582 | 8 | 8q22.3 |
| ariadne homolog, ubiquitin-conjugating enzyme E2 binding protein, 1 (Drosophila) | AI694332 | Hs.268787 | ARIH1 | 25820 | 15 | 15q24 |
| proline/serine-rich coiled-coil 1 | BC001425 | Hs.405925 | PSRC1 | 84722 | 1 | 1p13.3 |
| electron-transfer-flavoprotein, alpha polypeptide | NM_000126 | Hs.39925 | ETFA | 2108 | 15 | 15q23-q25 |
| guanine nucleotide binding protein-like 2 (nucleolar) | NM_013285 | Hs.75528 | GNL2 | 29889 | 1 | 1p34.3 |
| NADH dehydrogenase (ubiquinone) 1 alpha subcomplex, 6, 14kDa | NM_002490 | Hs.274416 | NDUFA6 | 4700 | 22 | 22q13.2 |
| transmembrane protein 184B | NM_012264 | Hs.728816 | TMEM184B | 25829 | 22 | 22q12 |
| branched chain ketoacid dehydrogenase kinase | NM_005881 | Hs.513520 | BCKDK | 10295 | 16 | 16p11.2 |
| zinc finger, MYM-type 4 | AA521508 | Hs.269211 | ZMYM4 | 9202 | 1 | 1p32-p34 |
| isocitrate dehydrogenase 3 (NAD+) alpha | AI826060 | Hs.591110 | IDH3A | 3419 | 15 | 15q25.1-q25.2 |
| syndecan 4 | NM_002999 | Hs.632267 | SDC4 | 6385 | 20 | 20q12 |
| growth arrest-specific 6 | NM_000820 | Hs.646346 | GAS6 | 2621 | 13 | 13q34 |
| major vault protein | NM_017458 | Hs.632177 | MVP | 9961 | 16 | 16p11.2 |
| ubiquinol-cytochrome c reductase hinge protein | NM_006004 | Hs.481571 | UQCRH | 7388 | 1 | 1p34.1 |
| CD2 (cytoplasmic tail) binding protein 2 | NM_006110 | Hs.202677 | CD2BP2 | 10421 | 16 | 16p11.2 |
| hepatitis B virus x interacting protein | NM_006402 | Hs.439815 | HBXIP | 10542 | 1 | 1p13.3 |
| phosphatidylserine decarboxylase | NM_014338 | Hs.420559 | PISD | 23761 | 22 | 22q12.2 |
| excision repair cross-complementing rodent repair deficiency, complementation group 5 | NM_000123 | Hs.258429 | ERCC5 | 2073 | 13 | 13q33 |
| v-myc myelocytomatosis viral oncogene homolog (avian) | NM_002467 | Hs.202453 | MYC | 4609 | 8 | 8q24.21 |
| UDP-galactose-4-epimerase | NM_000403 | Hs.632380 | GALE | 2582 | 1 | 1p36-p35 |
| CTP synthase | NM_001905 | Hs.473087 | CTPS | 1503 | 1 | 1p34.1 |
| neuroblastoma RAS viral (v-ras) oncogene homolog | NM_002524 | Hs.486502 | NRAS | 4893 | 1 | 1p13.2 |
| succinate dehydrogenase complex, subunit B, iron sulfur (Ip) | NM_003000 | Hs.465924 | SDHB | 6390 | 1 | 1p36.1-p35 |
| cell division cycle 16 homolog (S. cerevisiae) | NM_003903 | Hs.374127 | CDC16 | 8881 | 13 | 13q34 |
| phosphoinositide-3-kinase, regulatory subunit 3 (gamma) | BE622627 | Hs.655387 | PIK3R3 | 8503 | 1 | 1p34.1 |
| solute carrier family 20 (phosphate transporter), member 2 | NM_006749 | Hs.653173 | SLC20A2 | 6575 | 8 | 8p12-p11 |
| deoxynucleotidyltransferase, terminal, interacting protein 2 | NM_014597 | Hs.601998 | DNTTIP2 | 30836 | 1 | 1p22.1 |
| ATP-binding cassette, sub-family C (CFTR/MRP), member 1 | NM_004996 | Hs.391464 | ABCC1 | 4363 | 16 | 16p13.1 |
| target of myb1 (chicken) | NM_005488 | Hs.474705 | TOM1 | 10043 | 22 | 22q13.1 |
| developmentally regulated GTP binding protein 1 | NM_004147 | Hs.115242 | DRG1 | 4733 | 22 | 22q12.2 |
| transcription elongation factor B (SIII), polypeptide 1 (15kDa, elongin C) | N89607 | Hs.533437 | TCEB1 | 6921 | 8 | 8q21.11 |
| cell division cycle 20 homolog (S. cerevisiae) | NM_001255 | Hs.524947 | CDC20 | 991 | 1 | 1p34.1 |
| D-dopachrome tautomerase | NM_001355 | Hs.656723 | DDT | 1652 | 22 | 22q11.23 |
| zinc metallopeptidase (STE24 homolog, S. cerevisiae) | NM_005857 | Hs.132642 | ZMPSTE24 | 10269 | 1 | 1p34 |
| StAR-related lipid transfer (START) domain containing 3 | NM_006804 | Hs.728838 | STARD3 | 10948 | 17 | 17q11-q12 |
| lysophospholipase I | AF077198 | Hs.435850 | LYPLA1 | 10434 | 8 | 8q11.23 |
| inositol(myo)-1(or 4)-monophosphatase 1 | NM_005536 | Hs.656694 | IMPA1 | 3612 | 8 | 8q21.13-q21.3 |
| synovial sarcoma, X breakpoint 2 interacting protein | R52678 | Hs.22587 | SSX2IP | 117178 | 1 | 1p22.3 |
| breast carcinoma amplified sequence 2 | NM_005872 | Hs.22960 | BCAS2 | 10286 | 1 | 1p13.2 |
| pyruvate dehydrogenase complex, component X | NM_003477 | Hs.502315 | PDHX | 8050 | 11 | 11p13 |
| mitochondrial ribosomal protein L40 | NM_003776 | Hs.431307 | MRPL40 | 64976 | 22 | 22q11.21 |
| glycine amidinotransferase (L-arginine:glycine amidinotransferase) | NM_001482 | Hs.75335 | GATM | 2628 | 15 | 15q21.1 |
| lysine (K)-specific demethylase 4A | BC002558 | Hs.155983 | KDM4A | 9682 | 1 | 1p34.1 |
| uridine phosphorylase 1 | NM_003364 | Hs.488240 | UPP1 | 7378 | 7 | 7p12.3 |
| c-myc binding protein | D50692 | Hs.591506 | MYCBP | 26292 | 1 | 1p33-p32.2 |
| leucine-zipper-like transcription regulator 1 | NM_006767 | Hs.78788 | LZTR1 | 8216 | 22 | 22q11.1-q11.2 |
| leucine rich repeat containing 14 | NM_014665 | Hs.459391 | LRRC14 | 9684 | 8 | 8q24.3 |
| trafficking protein particle complex 3 | AF041432 | Hs.523131 | TRAPPC3 | 27095 | 1 | 1p34.3 |
| importin 13 | NM_014652 | Hs.158497 | IPO13 | 9670 | 1 | 1p34.1 |
| gamma-glutamyl hydrolase (conjugase, folylpolygammaglutamyl hydrolase) | NM_003878 | Hs.78619 | GGH | 8836 | 8 | 8q12.3 |
| polymerase (DNA directed), beta | NM_002690 | Hs.654484 | POLB | 5423 | 8 | 8p11.2 |
| NADH dehydrogenase (ubiquinone) 1 beta subcomplex, 5, 16kDa | NM_002492 | Hs.518424 | NDUFB5 | 4711 | 3 | 3q26.33 |
| tubulin, gamma complex associated protein 3 | NM_006322 | Hs.224152 | TUBGCP3 | 10426 | 13 | 13q34 |
| zinc finger protein 263 | NM_005741 | Hs.611475 | ZNF263 | 10127 | 16 | 16p13.3 |
| lethal giant larvae homolog 2 (Drosophila) | NM_004524 | Hs.514477 | LLGL2 | 3993 | 17 | 17q25.1 |
| zinc finger protein 217 | NM_006526 | Hs.155040 | ZNF217 | 7764 | 20 | 20q13.2 |
| polycomb group ring finger 2 | BC004858 | Hs.371617 | PCGF2 | 7703 | 17 | 17q12 |
| GRB2-associated binding protein 2 | NM_012296 | Hs.429434 | GAB2 | 9846 | 11 | 11q14.1 |
| LYR motif containing 1 | BE963444 | Hs.729017 | LYRM1 | 57149 | 16 | 16p11.2 |
| nucleotide binding protein 1 (MinD homolog, E. coli) | NM_002484 | Hs.81469 | NUBP1 | 4682 | 16 | 16p13.13 |
| frizzled homolog 6 (Drosophila) | NM_003506 | Hs.591863 | FZD6 | 8323 | 8 | 8q22.3-q23.1 |
| nucleoporin like 2 | NM_007342 | Hs.408241 | NUPL2 | 11097 | 7 | 7p15 |
| dynein, axonemal, light chain 4 | NM_005740 | Hs.632766 | DNAL4 | 10126 | 22 | 22q13.1 |
| zinc finger protein 593 | NM_015871 | Hs.477273 | ZNF593 | 51042 | 1 | 1p36.11 |
| choline kinase beta | NM_005198 | Hs.654827 | CHKB | 1120 | 22 | 22q13.33 |
| peptidylprolyl isomerase H (cyclophilin H) | NM_006347 | Hs.256639 | PPIH | 10465 | 1 | 1p34.1 |
| carnitine palmitoyltransferase 2 | NM_000098 | Hs.713535 | CPT2 | 1376 | 1 | 1p32 |
| estrogen receptor binding site associated, antigen, 9 | NM_004215 | Hs.409368 | EBAG9 | 9166 | 8 | 8q23 |
| serine/arginine-rich splicing factor 10 | NM_021993 | Hs.3530 | SRSF10 | 10772 | 1 | 1p36.11 |
| N-acetyltransferase 9 (GCN5-related, putative) | NM_015654 | Hs.144058 | NAT9 | 26151 | 17 | 17q25.1 |
| transcription termination factor, RNA polymerase II | AF080255 | Hs.486818 | TTF2 | 8458 | 1 | 1p22 |
| adenylate cyclase 9 | AB011092 | Hs.391860 | ADCY9 | 115 | 16 | 16p13.3 |
| HIRA interacting protein 3 | NM_003609 | Hs.592046 | HIRIP3 | 8479 | 16 | 16p11.2 |
| cell division cycle 7 homolog (S. cerevisiae) | NM_003503 | Hs.533573 | CDC7 | 8317 | 1 | 1p22 |
| DPH2 homolog (S. cerevisiae) | NM_001384 | Hs.632398 | DPH2 | 1802 | 1 | 1p34 |
| steroidogenic acute regulatory protein | NM_000349 | Hs.521535 | STAR | 6770 | 8 | 8p11.2 |
| RAD54-like (S. cerevisiae) | NM_003579 | Hs.642042 | RAD54L | 8438 | 1 | 1p32 |
| transmembrane protein 186 | NM_015421 | Hs.513330 | TMEM186 | 25880 | 16 | 16p13.2 |
| protein interacting with PRKCA 1 | NM_012407 | Hs.180871 | PICK1 | 9463 | 22 | 22q13.1 |
| regulator of chromosome condensation (RCC1) and BTB (POZ) domain containing protein 2 | NM_001268 | Hs.652712 | RCBTB2 | 1102 | 13 | 13q14.3 |
| non-metastatic cells 3, protein expressed in | NM_002513 | Hs.514065 | NME3 | 4832 | 16 | 16q13 |
| GTP cyclohydrolase I feedback regulator | NM_005258 | Hs.631717 | GCHFR | 2644 | 15 | 15q15 |
| hydroxyacylglutathione hydrolase | NM_005326 | Hs.157394 | HAGH | 3029 | 16 | 16p13.3 |
| origin recognition complex, subunit 1 | NM_004153 | Hs.17908 | ORC1 | 4998 | 1 | 1p32 |
| RWD domain containing 3 | NM_015485 | Hs.709591 | RWDD3 | 25950 | 1 | 1p21.3 |
| zinc finger protein 7 | NM_003416 | Hs.493218 | ZNF7 | 7553 | 8 | 8q24 |
| N-acetylglucosamine-1-phosphodiester alpha-N-acetylglucosaminidase | NM_016256 | Hs.21334 | NAGPA | 51172 | 16 | 16p13.3 |
| glycine C-acetyltransferase | NM_014291 | Hs.54609 | GCAT | 23464 | 22 | 22q13.1 |
| G-protein signaling modulator 2 | NM_013296 | Hs.584901 | GPSM2 | 29899 | 1 | 1p13.3 |
| SCL/TAL1 interrupting locus | NM_003035 | Hs.525198 | STIL | 6491 | 1 | 1p32 |
| adaptor-related protein complex 1, beta 1 subunit | NM_001127 | Hs.368794 | AP1B1 | 162 | 22 | 22q12 |
| ISG15 ubiquitin-like modifier | NM_005101 | Hs.458485 | ISG15 | 9636 | 1 | 1p36.33 |
| tubulin tyrosine ligase-like family, member 1 | NM_012263 | Hs.660298 | TTLL1 | 25809 | 22 | 22q13.1 |
| tryptase alpha/beta 1 | NM_003294 | Hs.405479 | TPSAB1 | 7177 | 16 | 16p13.3 |
| ubiquitin family domain containing 1 | NM_019116 | Hs.3459 | UBFD1 | 56061 | 16 | 16p12 |
| titin-cap (telethonin) | NM_003673 | Hs.77628 | TCAP | 8557 | 17 | 17q12 |
| BCL2-interacting killer (apoptosis-inducing) | NM_001197 | Hs.475055 | BIK | 638 | 22 | 22q13.31 |
| integrin, alpha M (complement component 3 receptor 3 subunit) | NM_000632 | Hs.172631 | ITGAM | 3684 | 16 | 16p11.2 |
| forkhead box J3 | NM_014947 | Hs.26023 | FOXJ3 | 22887 | 1 | 1p34.2 |
| regulating synaptic membrane exocytosis 2 | NM_014677 | Hs.655271 | RIMS2 | 9699 | 8 | 8q22.3 |
| gap junction protein, beta 5, 31.1kDa | NM_005268 | Hs.198249 | GJB5 | 2709 | 1 | 1p35.1 |
| GA binding protein transcription factor, beta subunit 1 | NM_002041 | Hs.654350 | GABPB1 | 2553 | 15 | 15q21.2 |
| immunoglobulin superfamily, member 6 | NM_005849 | Hs.530902 | IGSF6 | 10261 | 16 | 16p12.2 |
| regulator of chromosome condensation 1 | NM_001269 | Hs.469723 | RCC1 | 1104 | 1 | 1p36.1 |
| IQ motif containing C | NM_018134 | Hs.274356 | IQCC | 55721 | 1 | 1p36.11-p34.2 |
| endothelin 2 | NM_001956 | Hs.1407 | EDN2 | 1907 | 1 | 1p34 |
| solute carrier family 16, member 7 (monocarboxylic acid transporter 2) | NM_004731 | Hs.439643 | SLC16A7 | 9194 | 12 | 12q13 |
| sulfotransferase family, cytosolic, 1A, phenol-preferring, member 2 | NM_001054 | Hs.546304 | SULT1A2 | 6799 | 16 | 16p12.1 |
| tryptase beta 2 (gene/pseudogene) | NM_024164 | Hs.405479 | TPSB2 | 64499 | 16 | 16p13.3 |
| glomulin, FKBP associated protein | NM_007070 | Hs.49105 | GLMN | 11146 | 1 | 1p22.1 |
| zinc finger protein 643 | NM_023070 | Hs.133034 | ZNF643 | 65243 | 1 | 1p34.2 |
| solute carrier family 15 (oligopeptide transporter), member 1 | NM_005073 | Hs.436893 | SLC15A1 | 6564 | 13 | 13q33-q34 |
| KDEL (Lys-Asp-Glu-Leu) endoplasmic reticulum protein retention receptor 3 | NM_016657 | Hs.528305 | KDELR3 | 11015 | 22 | 22q13.1 |
| coagulation factor VII (serum prothrombin conversion accelerator) | NM_000131 | Hs.36989 | F7 | 2155 | 13 | 13q34 |
| transcription factor 15 (basic helix-loop-helix) | NM_004609 | Hs.437 | TCF15 | 6939 | 20 | 20p13 |
| zinc finger protein 200 | NM_003454 | Hs.632222 | ZNF200 | 7752 | 16 | 16p13.3 |
| cholinergic receptor, nicotinic, beta 4 | NM_000750 | Hs.624178 | CHRNB4 | 1143 | 15 | 15q24 |
| opioid receptor, kappa 1 | NM_000912 | Hs.106795 | OPRK1 | 4986 | 8 | 8q11.2 |
| mutY homolog (E. coli) | NM_012222 | Hs.271353 | MUTYH | 4595 | 1 | 1p34.1 |
| transition protein 2 (during histone to protamine replacement) | NM_005425 | Hs.513349 | TNP2 | 7142 | 16 | 16p13.13 |
| ferredoxin reductase | NM_004110 | Hs.69745 | FDXR | 2232 | 17 | 17q24-q25 |
| RNA binding protein with multiple splicing | NM_006867 | Hs.334587 | RBPMS | 11030 | 8 | 8p12 |
| cancer susceptibility candidate 3 | NM_007359 | Hs.592129 | CASC3 | 22794 | 17 | 17q11-q21.3 |
| Rho guanine nucleotide exchange factor (GEF) 16 | NM_014448 | Hs.87435 | ARHGEF16 | 27237 | 1 | 1p36.3 |
| protein Z, vitamin K-dependent plasma glycoprotein | NM_003891 | Hs.1011 | PROZ | 8858 | 13 | 13q34 |
| aurora kinase A | NM_003158 | Hs.250822 | AURKA | 6790 | 20 | 20q13 |
| hypothetical LOC81854 | NM_030970 | Hs.669490 | MGC3771 | 81854 | 16 | 16p13.3 |
| capping protein (actin filament) muscle Z-line, alpha 1 | NM_006135 | Hs.727604 | CAPZA1 | 829 | 1 | 1p13.2 |
| low density lipoprotein receptor-related protein 8, apolipoprotein e receptor | NM_017522 | Hs.726256 | LRP8 | 7804 | 1 | 1p34 |
| Y box binding protein 1 | BE966374 | Hs.473583 | YBX1 | 4904 | 1 | 1p34 |
| peroxiredoxin 1 | L19184 | Hs.180909 | PRDX1 | 5052 | 1 | 1p34.1 |
| voltage-dependent anion channel 3 | BC002456 | Hs.655340 | VDAC3 | 7419 | 8 | 8p11.2 |
| serine/threonine kinase 24 | AA586774 | Hs.508514 | STK24 | 8428 | 13 | 13q31.2-q32.3 |
| plexin B2 | BC004542 | Hs.3989 | PLXNB2 | 23654 | 22 | 22q13.33 |
| ERI1 exoribonuclease family member 3 | BC001072 | Hs.132497 | ERI3 | 79033 | 1 | 1p32 |
| chromosome 1 open reading frame 63 | AF247168 | Hs.259412 | C1orf63 | 57035 | 1 | 1p36.13-p35.1 |
| ubiquitin-conjugating enzyme E2 variant 2 | U62136 | Hs.491695 | UBE2V2 | 7336 | 8 | 8q11.21 |
| insulin receptor substrate 2 | BF700086 | Hs.442344 | IRS2 | 8660 | 13 | 13q34 |
| oxysterol binding protein-like 2 | AI753638 | Hs.473254 | OSBPL2 | 9885 | 20 | 20q13.3 |
| tumor suppressor candidate 3 | AU158251 | Hs.591845 | TUSC3 | 7991 | 8 | 8p22 |
| nuclear protein, transcriptional regulator, 1 | AF135266 | Hs.513463 | NUPR1 | 26471 | 16 | 16p11.2 |
| inhibitor of kappa light polypeptide gene enhancer in B-cells, kinase beta | AU153366 | Hs.597664 | IKBKB | 3551 | 8 | 8p11.2 |
| kinesin family member 2C | U63743 | Hs.720061 | KIF2C | 11004 | 1 | 1p34.1 |
| UDP-Gal:betaGlcNAc beta 1,4- galactosyltransferase, polypeptide 2 | BC002431 | Hs.632403 | B4GALT2 | 8704 | 1 | 1p34-p33 |
| THO complex 5 | BC003615 | Hs.75361 | THOC5 | 8563 | 22 | 22q12.2 |
| POZ (BTB) and AT hook containing zinc finger 1 | AI807017 | Hs.728132 | PATZ1 | 23598 | 22 | 22q12.2 |
| cerebellar degeneration-related protein 2, 62kDa | AL582414 | Hs.513430 | CDR2 | 1039 | 16 | 16p12.3 |
| ash2 (absent, small, or homeotic)-like (Drosophila) | AB020982 | Hs.521530 | ASH2L | 9070 | 8 | 8p11.2 |
| paraneoplastic antigen MA2 | AB020690 | Hs.591838 | PNMA2 | 10687 | 8 | 8p21.2 |
| oxysterol binding protein-like 3 | AI202969 | Hs.520259 | OSBPL3 | 26031 | 7 | 7p15 |
| solute carrier family 35 (UDP-glucuronic acid/UDP-N-acetylgalactosamine dual transporter), member D1 | AI769637 | Hs.213642 | SLC35D1 | 23169 | 1 | 1p32-p31 |
| gamma-glutamyltransferase 1 | L20490 | Hs.595809 | GGT1 | 2678 | 22 | 22q11.23 |
| solute carrier family 25 (mitochondrial carrier; citrate transporter), member 1 | U25147 | Hs.725671 | SLC25A1 | 6576 | 22 | 22q11.21 |
| isocitrate dehydrogenase 2 (NADP+), mitochondrial | U52144 | Hs.596461 | IDH2 | 3418 | 15 | 15q26.1 |
| ATP-binding cassette, sub-family A (ABC1), member 4 | U88667 | Hs.416707 | ABCA4 | 24 | 1 | 1p22 |
| mago-nashi homolog, proliferation-associated (Drosophila) | AF067173 | Hs.421576 | MAGOH | 4116 | 1 | 1p32.3 |
| tubulin tyrosine ligase-like family, member 3 | AF078842 | Hs.323342 | TTLL3 | 26140 | 3 | 3p25.3 |
| laminin, alpha 5 | BC003355 | Hs.473256 | LAMA5 | 3911 | 20 | 20q13.2-q13.3 |
| G protein-coupled receptor 18 | AF261135 | Hs.631765 | GPR18 | 2841 | 13 | 13q32 |
| peptidylprolyl isomerase E (cyclophilin E) | AF042386 | Hs.524690 | PPIE | 10450 | 1 | 1p32 |
| tocopherol (alpha) transfer protein | U21938 | Hs.69049 | TTPA | 7274 | 8 | 8q12.3 |
| growth factor receptor-bound protein 7 | AB008790 | Hs.86859 | GRB7 | 2886 | 17 | 17q12 |
| v-erb-b2 erythroblastic leukemia viral oncogene homolog 2, neuro/glioblastoma derived oncogene homolog (avian) | AF177761 | Hs.446352 | ERBB2 | 2064 | 17 | 17q11.2-q12 |
| MAK16 homolog (S. cerevisiae) | AF251062 | Hs.583805 | MAK16 | 84549 | 8 | 8p12 |
| methylmalonic aciduria (cobalamin deficiency) cblC type, with homocystinuria | BC006122 | Hs.13024 | MMACHC | 25974 | 1 | 1p34.1 |
| myosin, heavy chain 9, non-muscle | AI827941 | Hs.474751 | MYH9 | 4627 | 22 | 22q13.1 |
| importin 5 | AF339834 | Hs.712598 | IPO5 | 3843 | 13 | 13q32.2 |
| karyopherin alpha 6 (importin alpha 7) | AK002111 | Hs.470588 | KPNA6 | 23633 | 1 | 1p35.1 |
| adenylate kinase 2 | AW277253 | Hs.470907 | AK2 | 204 | 1 | 1p34 |
| mahogunin, ring finger 1 | AB011116 | Hs.526494 | MGRN1 | 23295 | 16 | 16p13.3 |
| HMG box domain containing 4 | AJ010070 | Hs.588815 | HMGXB4 | 10042 | 22 | 22q13.1 |
| WW domain containing E3 ubiquitin protein ligase 1 | AU155187 | Hs.655189 | WWP1 | 11059 | 8 | 8q21 |
| DDHD domain containing 2 | AB018268 | Hs.434966 | DDHD2 | 23259 | 8 | 8p11.23 |
| male-specific lethal 1 homolog (Drosophila) | AV721987 | Hs.532786 | MSL1 | 339287 | 17 | 17q21.1 |
| ankyrin repeat domain 46 | U79297 | Hs.530199 | ANKRD46 | 157567 | 8 | 8q22.2 |
| chromosome 1 open reading frame 216 | AL042729 | Hs.112023 | C1orf216 | 127703 | 1 | 1p34.3 |
| CCAAT/enhancer binding protein (C/EBP), delta | BF061054 | Hs.440829 | CEBPD | 1052 | 8 | 8p11.2-p11.1 |
| neural precursor cell expressed, developmentally down-regulated 4 | D42055 | Hs.1565 | NEDD4 | 4734 | 15 | 15q |
| mediator complex subunit 24 | AI023317 | Hs.462983 | MED24 | 9862 | 17 | 17q21.1 |
| RER1 retention in endoplasmic reticulum 1 homolog (S. cerevisiae) | AI818736 | Hs.591454 | RER1 | 11079 | 1 | 1p36 |
| malonyl CoA:ACP acyltransferase (mitochondrial) | AL022237 | Hs.349111 | MCAT | 27349 | 22 | 22q13.31 |
| synovial sarcoma translocation gene on chromosome 18-like 1 | AB014593 | Hs.154429 | SS18L1 | 26039 | 20 | 20q13.3 |
| component of oligomeric golgi complex 7 | R61519 | Hs.185807 | COG7 | 91949 | 16 | 16p12.2 |
| death inducer-obliterator 1 | AL035669 | Hs.517172 | DIDO1 | 11083 | 20 | 20q13.33 |
| cerebellar degeneration-related protein 2-like | AI422335 | Hs.78358 | CDR2L | 30850 | 17 | 17q25.1 |
| ERI1 exoribonuclease family member 2 | N64622 | Hs.248437 | ERI2 | 112479 | 16 | 16p12.3 |
| nephronophthisis 4 | AB014573 | Hs.462348 | NPHP4 | 261734 | 1 | 1p36.22 |
| cyclin-dependent kinase 12 | AW305119 | Hs.416108 | CDK12 | 51755 | 17 | 17q12 |
| transformer 2 alpha homolog (Drosophila) | AW978896 | Hs.445652 | TRA2A | 29896 | 7 | 7p15.3 |
| zinc finger protein 500 | AB011129 | Hs.513316 | ZNF500 | 26048 | 16 | 16p13.3 |
| mediator complex subunit 8 | AA421957 | Hs.301756 | MED8 | 112950 | 1 | 1p34.2 |
| Rab geranylgeranyltransferase, beta subunit | AA129753 | Hs.78948 | RABGGTB | 5876 | 1 | 1p31 |
| xylosyltransferase I | AI693140 | Hs.22907 | XYLT1 | 64131 | 16 | 16p12.3 |
| intraflagellar transport 27 homolog (Chlamydomonas) | AL037167 | Hs.415172 | IFT27 | 11020 | 22 | 22q13.1 |
| CAP, adenylate cyclase-associated protein 1 (yeast) | AA806142 | Hs.370581 | CAP1 | 10487 | 1 | 1p34.2 |
| TM2 domain containing 1 | AA012917 | Hs.656790 | TM2D1 | 83941 | 1 | 1p31.3 |
| far upstream element (FUSE) binding protein 1 | AA156865 | Hs.567380 | FUBP1 | 8880 | 1 | 1p31.1 |
| heat shock protein family B (small), member 11 | AV700696 | Hs.525462 | HSPB11 | 51668 | 1 | 1p32 |
| tight junction protein 1 (zona occludens 1) | AA813018 | Hs.510833 | TJP1 | 7082 | 15 | 15q13 |
| proline dehydrogenase (oxidase) 1 | AA074145 | Hs.517352 | PRODH | 5625 | 22 | 22q11.21 |
| solute carrier family 25 (mitochondrial carrier; peroxisomal membrane protein, 34kDa), member 17 | AL049764 | Hs.474938 | SLC25A17 | 10478 | 22 | 22q13.2 |
| outer dense fiber of sperm tails 1 | NM_024410 | Hs.159274 | ODF1 | 4956 | 8 | 8q22.3 |
| proline synthetase co-transcribed homolog (bacterial) | NM_007198 | Hs.304792 | PROSC | 11212 | 8 | 8p11.2 |
| InaD-like (Drosophila) | AJ001306 | Hs.478125 | INADL | 10207 | 1 | 1p31.3 |
| ribosomal protein L23 | AK021960 | Hs.406300 | RPL23 | 9349 | 17 | 17q |
| acyl-CoA thioesterase 11 | AK023937 | Hs.729424 | ACOT11 | 26027 | 1 | 1p32.3 |
| tripartite motif containing 33 | AU136587 | Hs.26837 | TRIM33 | 51592 | 1 | 1p13.1 |
| small nucleolar RNA host gene 3 (non-protein coding) | AJ006835 | Hs.469723 | SNHG3 | 8420 | 1 | 1p36.1 |
| formin binding protein 1-like | AW270932 | Hs.726017 | FNBP1L | 54874 | 1 | 1p22.1 |
| protein tyrosine phosphatase, receptor type, F | AU158443 | Hs.272062 | PTPRF | 5792 | 1 | 1p34 |
| growth factor receptor-bound protein 2 | L29511 | Hs.444356 | GRB2 | 2885 | 17 | 17q24-q25 |
| leucine rich repeat containing 42 | AL031427 | Hs.40094 | LRRC42 | 115353 | 1 | 1p33-p32.1 |
| transmembrane protein 111 | AU147317 | Hs.475392 | TMEM111 | 55831 | 3 | 3p25.3 |
| sulfotransferase family, cytosolic, 1A, phenol-preferring, member 1 | U37025 | Hs.567342 | SULT1A1 | 6817 | 16 | 16p12.1 |
| TCR gamma alternate reading frame protein | AA310709 | Hs.534032 | TARP | 445347 | 7 | 7p15-p14 |
| gasdermin B | AK025174 | Hs.306777 | GSDMB | 55876 | 17 | 17q12 |
| microtubule associated serine/threonine kinase 2 | AK025352 | Hs.319481 | MAST2 | 23139 | 1 | 1p34.1 |
| activating signal cointegrator 1 complex subunit 2 | AL096741 | Hs.517438 | ASCC2 | 84164 | 22 | 22q12.1 |
| Ras protein-specific guanine nucleotide-releasing factor 1 | AL359931 | Hs.459035 | RASGRF1 | 5923 | 15 | 15q24.2 |
| DEAD (Asp-Glu-Ala-Asp) box polypeptide 27 | AL512707 | Hs.65234 | DDX27 | 55661 | 20 | 20q13.13 |
| acyl-CoA thioesterase 7 | AL031848 | Hs.126137 | ACOT7 | 11332 | 1 | 1p36 |
| myosin, heavy chain 7B, cardiac muscle, beta | AK000947 | Hs.414122 | MYH7B | 57644 | 20 | 20q11.22 |
| small nuclear ribonucleoprotein 40kDa (U5) | AL157420 | Hs.33962 | SNRNP40 | 9410 | 1 | 1p35.2 |
| mitochondrial ribosomal protein S11 | BC000200 | Hs.111286 | MRPS11 | 64963 | 15 | 15q25 |
| platelet-derived growth factor beta polypeptide (simian sarcoma viral (v-sis) oncogene homolog) | AU150748 | Hs.1976 | PDGFB | 5155 | 22 | 22q12.3-q13.1 |
| proteasome (prosome, macropain) subunit, alpha type, 7 | AL078633 | Hs.233952 | PSMA7 | 5688 | 20 | 20q13.33 |
| minichromosome maintenance complex component 5 | AA807529 | Hs.517582 | MCM5 | 4174 | 22 | 22q13.1 |
| DiGeorge syndrome critical region gene 14 | AL137713 | Hs.517407 | DGCR14 | 8220 | 22 | 22q11.2 |
| Rh blood group, CcEe antigens | X63095 | Hs.449968 | RHCE | 6006 | 1 | 1p36.11 |
| enolase 1, (alpha) | U88968 | Hs.517145 | ENO1 | 2023 | 1 | 1p36.2 |
| nuclear factor of activated T-cells, cytoplasmic, calcineurin-dependent 2 interacting protein | AI478300 | Hs.513470 | NFATC2IP | 84901 | 16 | 16p11.2 |
| zinc finger, CCHC domain containing 11 | R25849 | Hs.655407 | ZCCHC11 | 23318 | 1 | 1p32.3 |
| rhomboid domain containing 3 | AA018187 | Hs.106730 | RHBDD3 | 25807 | 22 | 22q12.2 |
| SERPINE1 mRNA binding protein 1 | NM_015640 | Hs.530412 | SERBP1 | 26135 | 1 | 1p31 |
| hematological and neurological expressed 1 | NM_016185 | Hs.532803 | HN1 | 51155 | 17 | 17q25.1 |
| tripartite motif containing 44 | BF431488 | Hs.192103 | TRIM44 | 54765 | 11 | 11p13 |
| chromosome 16 open reading frame 58 | NM_022744 | Hs.9003 | C16orf58 | 64755 | 16 | 16p11.2 |
| KIAA0319-like | NM_024874 | Hs.456507 | KIAA0319L | 79932 | 1 | 1p34.2 |
| carbohydrate kinase domain containing | NM_018210 | Hs.408324 | CARKD | 55739 | 13 | 13q34 |
| translocase of outer mitochondrial membrane 22 homolog (yeast) | NM_020243 | Hs.595072 | TOMM22 | 56993 | 22 | 22q12-q13 |
| NOP10 ribonucleoprotein homolog (yeast) | NM_018648 | Hs.14317 | NOP10 | 55505 | 15 | 15q14-q15 |
| mortality factor 4 like 1 | NM_006791 | Hs.374503 | MORF4L1 | 10933 | 15 | 15q24 |
| sulfide quinone reductase-like (yeast) | NM_021199 | Hs.511251 | SQRDL | 58472 | 15 | 15q15 |
| nucleoporin 85kDa | NM_024844 | Hs.362817 | NUP85 | 79902 | 17 | 17q25.1 |
| elongation of very long chain fatty acids (FEN1/Elo2, SUR4/Elo3, yeast)-like 1 | NM_016031 | Hs.25597 | ELOVL1 | 64834 | 1 | 1p34.2 |
| stannin | AF070673 | Hs.618526 | SNN | 8303 | 16 | 16p13 |
| arginine and glutamate rich 1 | NM_018011 | Hs.508644 | ARGLU1 | 55082 | 13 | 13q33.3 |
| dCTP pyrophosphatase 1 | NM_024096 | Hs.632191 | DCTPP1 | 79077 | 16 | 16p11.2 |
| transmembrane protein 48 | NM_018087 | Hs.476525 | TMEM48 | 55706 | 1 | 1p32.3 |
| high-mobility group 20A | NM_018200 | Hs.69594 | HMG20A | 10363 | 15 | 15q24 |
| UTP11-like, U3 small nucleolar ribonucleoprotein, (yeast) | NM_016037 | Hs.472038 | UTP11L | 51118 | 1 | 1p34.3 |
| RAB22A, member RAS oncogene family | NM_020673 | Hs.529044 | RAB22A | 57403 | 20 | 20q13.32 |
| S100P binding protein | NM_022753 | Hs.440880 | S100PBP | 64766 | 1 | 1p35.1 |
| tetratricopeptide repeat domain 4 | NM_004623 | Hs.729029 | TTC4 | 7268 | 1 | 1p32.3 |
| asparagine-linked glycosylation 12, alpha-1,6-mannosyltransferase homolog (S. cerevisiae) | NM_024105 | Hs.526711 | ALG12 | 79087 | 22 | 22q13.33 |
| ubiquitin-conjugating enzyme E2W (putative) | NM_018299 | Hs.728774 | UBE2W | 55284 | 8 | 8q21.11 |
| uridine-cytidine kinase 1-like 1 | NM_017859 | Hs.504998 | UCKL1 | 54963 | 20 | 20q13.33 |
| chromosome 8 open reading frame 4 | NM_020130 | Hs.591849 | C8orf4 | 56892 | 8 | 8p11.2 |
| leucine rich repeat containing 40 | NM_017768 | Hs.147836 | LRRC40 | 55631 | 1 | 1p31.1 |
| tRNA isopentenyltransferase 1 | NM_017646 | Hs.356554 | TRIT1 | 54802 | 1 | 1p34.2 |
| DiGeorge syndrome critical region gene 8 | NM_022775 | Hs.643452 | DGCR8 | 54487 | 22 | 22q11.2 |
| Rap guanine nucleotide exchange factor (GEF)-like 1 | NM_016339 | Hs.632254 | RAPGEFL1 | 51195 | 17 | 17q21.1 |
| stromal cell-derived factor 2-like 1 | NM_022044 | Hs.303116 | SDF2L1 | 23753 | 22 | 22q11.21 |
| exosome component 4 | NM_019037 | Hs.632041 | EXOSC4 | 54512 | 8 | 8q24.3 |
| chromosome 1 open reading frame 109 | NM_017850 | Hs.272673 | C1orf109 | 54955 | 1 | 1p34.3 |
| zinc finger, matrin-type 5 | NM_019103 | Hs.713647 | ZMAT5 | 55954 | 22 | 22cen-q12.3 |
| WD repeat domain 3 | NM_006784 | Hs.310809 | WDR3 | 10885 | 1 | 1p12 |
| zinc finger protein 768 | NM_024671 | Hs.85658 | ZNF768 | 79724 | 16 | 16p11.2 |
| BRF2, subunit of RNA polymerase III transcription initiation factor, BRF1-like | NM_018310 | Hs.274136 | BRF2 | 55290 | 8 | 8p11.23 |
| zinc finger protein 668 | NM_024706 | Hs.102928 | ZNF668 | 79759 | 16 | 16p11.2 |
| meteorin, glial cell differentiation regulator | NM_024042 | Hs.533772 | METRN | 79006 | 16 | 16p13.3 |
| amidohydrolase domain containing 2 | NM_015944 | Hs.728841 | AMDHD2 | 51005 | 16 | 16p13.3 |
| armadillo repeat containing 7 | NM_024585 | Hs.413047 | ARMC7 | 79637 | 17 | 17q25.1 |
| DCN1, defective in cullin neddylation 1, domain containing 2 (S. cerevisiae) | NM_018185 | Hs.728957 | DCUN1D2 | 55208 | 13 | 13q34 |
| chromosome 8 open reading frame 41 | NM_025115 | Hs.583805 | C8orf41 | 80185 | 8 | 8p12 |
| ciliary rootlet coiled-coil, rootletin pseudogene 3 | AF119868 | Hs.597881 | CROCCP3 | 114819 | 1 | 1p36.13 |
| peroxisomal biogenesis factor 26 | AI817074 | Hs.517400 | PEX26 | 55670 | 22 | 22q11.21 |
| enhancer of mRNA decapping 3 homolog (S. cerevisiae) | NM_025083 | Hs.682454 | EDC3 | 80153 | 15 | 15q24.1 |
| mitochondrial ribosomal protein L46 | NM_022163 | Hs.534261 | MRPL46 | 26589 | 15 | 15q24-q25 |
| chromosome 1 open reading frame 159 | NM_017891 | Hs.235095 | C1orf159 | 54991 | 1 | 1p36.33 |
| apoptosis, caspase activation inhibitor | NM_020371 | Hs.555966 | AVEN | 57099 | 15 | 15q13.1 |
| chromosome 1 open reading frame 50 | NM_024097 | Hs.148845 | C1orf50 | 79078 | 1 | 1p34.2 |
| chromosome 1 open reading frame 163 | NM_023077 | Hs.349905 | C1orf163 | 65260 | 1 | 1p32.3 |
| eukaryotic translation initiation factor 2C, 3 | NM_024852 | Hs.657659 | EIF2C3 | 192669 | 1 | 1p34.3 |
| RNA polymerase II associated protein 2 | NM_024813 | Hs.444421 | RPAP2 | 79871 | 1 | 1p22.1 |
| defects in morphology 1 homolog (S. cerevisiae) | NM_022774 | Hs.59584 | DEM1 | 64789 | 1 | 1p34.2 |
| coiled-coil domain containing 21 | NM_022778 | Hs.63795 | CCDC21 | 64793 | 1 | 1p36.11 |
| RAB20, member RAS oncogene family | NM_017817 | Hs.729392 | RAB20 | 55647 | 13 | 13q34 |
| PDZK1 interacting protein 1 | NM_005764 | Hs.431099 | PDZK1IP1 | 10158 | 1 | 1p33 |
| asparagine-linked glycosylation 6, alpha-1,3-glucosyltransferase homolog (S. cerevisiae) | NM_013339 | Hs.258501 | ALG6 | 29929 | 1 | 1p31.3 |
| RAB11 family interacting protein 1 (class I) | NM_025151 | Hs.191179 | RAB11FIP1 | 80223 | 8 | 8p11.22 |
| frizzled homolog 3 (Drosophila) | NM_017412 | Hs.40735 | FZD3 | 7976 | 8 | 8p21 |
| mitochondrial ribosomal protein S28 | NM_014018 | Hs.521124 | MRPS28 | 28957 | 8 | 8q21.1-q21.2 |
| solute carrier organic anion transporter family, member 4A1 | NM_016354 | Hs.235782 | SLCO4A1 | 28231 | 20 | 20q13.33 |
| chromosome 1 open reading frame 135 | NM_024037 | Hs.725400 | C1orf135 | 79000 | 1 | 1p36.11 |
| transmembrane protein 104 | NM_017728 | Hs.370262 | TMEM104 | 54868 | 17 | 17q25.1 |
| bone morphogenetic protein 8a | NM_024732 | Hs.472497 | BMP8A | 353500 | 1 | 1p34.3 |
| zinc finger, MYM-type 1 | NM_024772 | Hs.471243 | ZMYM1 | 79830 | 1 | 1p34.3 |
| chromosome 1 open reading frame 103 | NM_018372 | Hs.25245 | C1orf103 | 55791 | 1 | 1p13.3 |
| chromosome 20 open reading frame 195 | NM_024059 | Hs.197755 | C20orf195 | 79025 | 20 | 20q13.33 |
| PQ loop repeat containing 2 | NM_017765 | Hs.647620 | PQLC2 | 54896 | 1 | 1p36.13 |
| mitochondrial ribosomal protein L20 | NM_017971 | Hs.182698 | MRPL20 | 55052 | 1 | 1p36.3-p36.2 |
| leucine proline-enriched proteoglycan (leprecan) 1 | NM_022356 | Hs.720014 | LEPRE1 | 64175 | 1 | 1p34.1 |
| solute carrier family 2 (facilitated glucose transporter), member 10 | NM_030777 | Hs.305971 | SLC2A10 | 81031 | 20 | 20q13.1 |
| DENN/MADD domain containing 2D | NM_024901 | Hs.557850 | DENND2D | 79961 | 1 | 1p13.3 |
| potassium large conductance calcium-activated channel, subfamily M beta member 3 | NM_014407 | Hs.591285 | KCNMB3 | 27094 | 3 | 3q26.3-q27 |
| GDNF family receptor alpha 4 | NM_022139 | Hs.302025 | GFRA4 | 64096 | 20 | 20p13-p12 |
| transmembrane 7 superfamily member 4 | NM_030788 | Hs.652230 | TM7SF4 | 81501 | 8 | 8q23 |
| cell division cycle associated 8 | BC001651 | Hs.524571 | CDCA8 | 55143 | 1 | 1p34.3 |
| ER lipid raft associated 2 | T90773 | Hs.705490 | ERLIN2 | 11160 | 8 | 8p11.2 |
| E2F transcription factor 5, p130-binding | U15642 | Hs.445758 | E2F5 | 1875 | 8 | 8q21.2 |
| YTH domain family, member 1 | AL096828 | Hs.11747 | YTHDF1 | 54915 | 20 | 20q13.33 |
| splicing factor proline/glutamine-rich | AV705803 | Hs.355934 | SFPQ | 6421 | 1 | 1p34.3 |
| post-GPI attachment to proteins 3 | BF033007 | Hs.462971 | PGAP3 | 93210 | 17 | 17q12 |
| coiled-coil domain containing 101 | BE544663 | Hs.655476 | CCDC101 | 112869 | 16 | 16p11.2 |
| CASK interacting protein 2 | AI970096 | Hs.274408 | CASKIN2 | 57513 | 17 | 17q25.1 |
| zinc finger protein 335 | NM_022095 | Hs.174193 | ZNF335 | 63925 | 20 | 20q13.12 |
| RNA pseudouridylate synthase domain containing 2 | AI571208 | Hs.173311 | RPUSD2 | 27079 | 15 | 15q13.3 |
| Pvt1 oncogene (non-protein coding) | AW451806 | Hs.133107 | PVT1 | 5820 | 8 | 8q24 |
| CCR4-NOT transcription complex, subunit 2 | BG105204 | Hs.133350 | CNOT2 | 4848 | 12 | 12q15 |
| RRN3 RNA polymerase I transcription factor homolog (S. cerevisiae) | AL110238 | Hs.460078 | RRN3 | 54700 | 16 | 16p12 |
| glucocorticoid modulatory element binding protein 2 | AL133646 | Hs.473286 | GMEB2 | 26205 | 20 | 20q13.33 |
| akirin 1 | AI205764 | Hs.293563 | AKIRIN1 | 79647 | 1 | 1p34.3 |
| ribosomal L24 domain containing 1 | AF165521 | Hs.274772 | RSL24D1 | 51187 | 15 | 15q21 |
| polymerase (RNA) III (DNA directed) polypeptide E (80kD) | AK023160 | Hs.460298 | POLR3E | 55718 | 16 | 16p12.2 |
| Ras-related GTP binding C | AK023373 | Hs.532461 | RRAGC | 64121 | 1 | 1p34 |
| Wolf-Hirschhorn syndrome candidate 1-like 1 | AI697751 | Hs.608111 | WHSC1L1 | 54904 | 8 | 8p11.2 |
| SLC2A4 regulator | BE898559 | Hs.435126 | SLC2A4RG | 56731 | 20 | 20q13.33 |
| alkaline ceramidase 3 | N51263 | Hs.23862 | ACER3 | 55331 | 11 | 11q13.5 |
| yrdC domain containing (E. coli) | BE464161 | Hs.301564 | YRDC | 79693 | 1 | 1p34.3 |
| CWC25 spliceosome-associated protein homolog (S. cerevisiae) | H79861 | Hs.406223 | CWC25 | 54883 | 17 | 17q12 |
| tryptophanyl tRNA synthetase 2, mitochondrial | BF515963 | Hs.523506 | WARS2 | 10352 | 1 | 1p12 |
| tRNA selenocysteine 1 associated protein 1 | BC000680 | Hs.533626 | TRNAU1AP | 54952 | 1 | 1p35.3 |
| hydroxy-delta-5-steroid dehydrogenase, 3 beta- and steroid delta-isomerase 7 | BC004929 | Hs.460618 | HSD3B7 | 80270 | 16 | 16p11.2 |
| RAB8B, member RAS oncogene family | AB038995 | Hs.389733 | RAB8B | 51762 | 15 | 15q22.2 |
| DNA cross-link repair 1B | AI703304 | Hs.591412 | DCLRE1B | 64858 | 1 | 1p13.2 |
| BCL2-associated athanogene 4 | AF111116 | Hs.194726 | BAG4 | 9530 | 8 | 8p11.23 |
| DEP domain containing 1 | AK000490 | Hs.445098 | DEPDC1 | 55635 | 1 | 1p31.2 |
| cold shock domain containing E1, RNA-binding | AI423180 | Hs.69855 | CSDE1 | 7812 | 1 | 1p22 |
| mitochondrial ribosomal protein L37 | AF325707 | Hs.584908 | MRPL37 | 51253 | 1 | 1p32.1 |
| cyclin L2 | AF251294 | Hs.515704 | CCNL2 | 81669 | 1 | 1p36.33 |
| thioredoxin domain containing 12 (endoplasmic reticulum) | AF131758 | Hs.476033 | TXNDC12 | 51060 | 1 | 1p32.3 |
| chromosome 22 open reading frame 13 | BC004144 | Hs.9850 | C22orf13 | 83606 | 22 | 22q11.2 |
| transmembrane protein 85 | AF151018 | Hs.250905 | TMEM85 | 51234 | 15 | 15q14 |
| ADP-ribosylhydrolase like 2 | AF212236 | Hs.18021 | ADPRHL2 | 54936 | 1 | 1p34.3 |
| zinc finger, CCHC domain containing 17 | AF151077 | Hs.524094 | ZCCHC17 | 51538 | 1 | 1p35.2 |
| NADH dehydrogenase (ubiquinone) 1 beta subcomplex, 10, 22kDa | AF044954 | Hs.513266 | NDUFB10 | 4716 | 16 | 16p13.3 |
| leucine zipper, down-regulated in cancer 1-like | AL136553 | Hs.706701 | LDOC1L | 84247 | 22 | 22q13.31 |
| TatD DNase domain containing 1 | AF212250 | Hs.170568 | TATDN1 | 83940 | 8 | 8q24.13 |
| MAD2 mitotic arrest deficient-like 2 (yeast) | AF080398 | Hs.19400 | MAD2L2 | 10459 | 1 | 1p36 |
| mitochondrial ribosomal protein S15 | AF265439 | Hs.352839 | MRPS15 | 64960 | 1 | 1p34.3 |
| GLIS family zinc finger 2 | AA705182 | Hs.592087 | GLIS2 | 84662 | 16 | 16p13.3 |
| G protein-coupled receptor 160 | BC000181 | Hs.231320 | GPR160 | 26996 | 3 | 3q26.2-q27 |
| oxysterol binding protein 2 | BE501253 | Hs.517546 | OSBP2 | 23762 | 22 | 22q12.2 |
| tumor necrosis factor (ligand) superfamily, member 13b | AW151360 | Hs.525157 | TNFSF13B | 10673 | 13 | 13q32-q34 |
| mex-3 homolog B (C. elegans) | AL136778 | Hs.104744 | MEX3B | 84206 | 15 | 15q25.2 |
| zinc finger, RAN-binding domain containing 2 | AF065391 | Hs.194718 | ZRANB2 | 9406 | 1 | 1p31 |
| apolipoprotein L, 4 | AY014914 | Hs.726733 | APOL4 | 80832 | 22 | 22q11.2-q13.2 |
| Mov10, Moloney leukemia virus 10, homolog (mouse) | BC002548 | Hs.514941 | MOV10 | 4343 | 1 | 1p13.2 |
| oxidation resistance 1 | AF309387 | Hs.148778 | OXR1 | 55074 | 8 | 8q23 |
| DNA methyltransferase 1 associated protein 1 | AL136657 | Hs.8008 | DMAP1 | 55929 | 1 | 1p34 |
| X-prolyl aminopeptidase (aminopeptidase P) 3, putative | BC001208 | Hs.529163 | XPNPEP3 | 63929 | 22 | 22q13.2 |
| UBA domain containing 2 | BC004528 | Hs.508545 | UBAC2 | 337867 | 13 | 13q32.3 |
| Smith-Magenis syndrome chromosome region, candidate 7-like | AL136768 | Hs.714252 | SMCR7L | 54471 | 22 | 22q13 |
| TM2 domain containing 2 | AF353991 | Hs.7471 | TM2D2 | 83877 | 8 | 8p11.22 |
| chromosome 17 open reading frame 37 | BC006006 | Hs.333526 | C17orf37 | 84299 | 17 | 17q12 |
| hypothetical protein MGC12982 | BC006113 | Hs.127762 | MGC12982 | 84793 | 1 | 1p33 |
| regulator of chromosome condensation 2 | AB040903 | Hs.380857 | RCC2 | 55920 | 1 | 1p36.13 |
| KIAA1522 | AB040955 | Hs.591502 | KIAA1522 | 57648 | 1 | 1p35.1 |
| chromosome 12 open reading frame 23 | AK001731 | Hs.257664 | C12orf23 | 90488 | 12 | 12q23.3 |
| 1-acylglycerol-3-phosphate O-acyltransferase 6 (lysophosphatidic acid acyltransferase, zeta) | BF513102 | Hs.355753 | AGPAT6 | 137964 | 8 | 8p11.21 |
| N-acetylglucosamine-1-phosphate transferase, gamma subunit | AF302786 | Hs.241575 | GNPTG | 84572 | 16 | 16p13.3 |
| coiled-coil-helix-coiled-coil-helix domain containing 10 | AI814909 | Hs.66915 | CHCHD10 | 400916 | 22 | 22q11.23 |
| serine/threonine kinase 40 | BF063301 | Hs.603131 | STK40 | 83931 | 1 | 1p34.3 |
| solute carrier family 35, member C2 | AL518563 | Hs.593344 | SLC35C2 | 51006 | 20 | 20q13.12 |
| PCI domain containing 2 | AU144029 | Hs.508769 | PCID2 | 55795 | 13 | 13q34 |
| eukaryotic translation initiation factor 2 alpha kinase 4 | AB037759 | Hs.656673 | EIF2AK4 | 440275 | 15 | 15q15.1 |
| protein phosphatase 1, regulatory (inhibitor) subunit 16A | AI742931 | Hs.521937 | PPP1R16A | 84988 | 8 | 8q24.3 |
| INO80 homolog (S. cerevisiae) | AI659419 | Hs.292949 | INO80 | 54617 | 15 | 15q15.1 |
| chromosome 1 open reading frame 212 | R75637 | Hs.27160 | C1orf212 | 113444 | 1 | 1p34.3 |
| mediator complex subunit 1 | AI423072 | Hs.643754 | MED1 | 5469 | 17 | 17q12 |
| mesoderm induction early response 1 homolog (Xenopus laevis) | AI668786 | Hs.605432 | MIER1 | 57708 | 1 | 1p31.3 |
| chromosome 1 open reading frame 122 | BE408081 | Hs.532749 | C1orf122 | 127687 | 1 | 1p34.3 |
| chromosome 8 open reading frame 40 | AV711345 | Hs.655320 | C8orf40 | 114926 | 8 | 8p11.21 |
| transmembrane protein 54 | AL545105 | Hs.534521 | TMEM54 | 113452 | 1 | 1p35-p34 |
| RAS p21 protein activator 3 | AI684746 | Hs.593075 | RASA3 | 22821 | 13 | 13q34 |
| RAP2A, member of RAS oncogene family | AI963476 | Hs.508480 | RAP2A | 5911 | 13 | 13q34 |
| LSM10, U7 small nuclear RNA associated | AL542359 | Hs.654622 | LSM10 | 84967 | 1 | 1p34.3 |
| sorting nexin 29 | AL044019 | Hs.585745 | SNX29 | 92017 | 16 | 16p13.13-p13.12 |
| alkB, alkylation repair homolog 2 (E. coli) | AI865555 | Hs.374458 | ALKBH2 | 121642 | 12 | 12q24.11 |
| myeloid/lymphoid or mixed-lineage leukemia (trithorax homolog, Drosophila); translocated to, 6 | BE677453 | Hs.91531 | MLLT6 | 4302 | 17 | 17q21 |
| chromosome 7 open reading frame 40 | AI937446 | Hs.25892 | C7orf40 | 285958 | 7 | 7p13 |
| THUMP domain containing 3 | AI921788 | Hs.443081 | THUMPD3 | 25917 | 3 | 3p25.3 |
| F-box protein 22 | AW294765 | Hs.591115 | FBXO22 | 26263 | 15 | 15q24.2 |
| regulatory solute carrier protein, family 1, member 1 | AL565415 | Hs.239459 | RSC1A1 | 6248 | 1 | 1p36.1 |
| hook homolog 1 (Drosophila) | AA618420 | Hs.378836 | HOOK1 | 51361 | 1 | 1p32.1 |
| transmembrane protein 125 | AV709406 | Hs.104476 | TMEM125 | 128218 | 1 | 1p34.2 |
| HEN1 methyltransferase homolog 1 (Arabidopsis) | BE502436 | Hs.7962 | HENMT1 | 113802 | 1 | 1p13.3 |
| basic transcription factor 3-like 4 | AL568049 | Hs.429839 | BTF3L4 | 91408 | 1 | 1p32.3 |
| chromosome 17 open reading frame 28 | AW139549 | Hs.11067 | C17orf28 | 283987 | 17 | 17q25.1 |
| necdin-like 2 | AA627644 | Hs.728271 | NDNL2 | 56160 | 15 | 15q13.1 |
| OMA1 homolog, zinc metallopeptidase (S. cerevisiae) | AI927931 | Hs.425769 | OMA1 | 115209 | 1 | 1p32.2-p32.1 |
| diacylglycerol O-acyltransferase 2 | AW469523 | Hs.129798 | DGAT2 | 84649 | 11 | 11q13.5 |
| FLYWCH family member 2 | AL542248 | Hs.534525 | FLYWCH2 | 114984 | 16 | 16p13.3 |
| leucine zipper and CTNNBIP1 domain containing | BG180887 | Hs.327252 | LZIC | 84328 | 1 | 1p36.22 |
| family with sequence similarity 83, member H | AI949095 | Hs.67776 | FAM83H | 286077 | 8 | 8q24.3 |
| phosphatidic acid phosphatase type 2 domain containing 1B | BF111651 | Hs.567619 | PPAPDC1B | 84513 | 8 | 8p11.23 |
| zinc finger protein 828 | BC004820 | Hs.7542 | ZNF828 | 283489 | 13 | 13q34 |
| RCAN family member 3 | N25986 | Hs.656799 | RCAN3 | 11123 | 1 | 1p35.3-p33 |
| zinc and ring finger 3 | AK022809 | Hs.655242 | ZNRF3 | 84133 | 22 | 22q12.1 |
| transcription elongation factor A (SII), 3 | AI675780 | Hs.446354 | TCEA3 | 6920 | 1 | 1p36.12 |
| activity-dependent neuroprotector homeobox | BG149849 | Hs.570355 | ADNP | 23394 | 20 | 20q13.13 |
| syntrophin, beta 1 (dystrophin-associated protein A1, 59kDa, basic component 1) | AK025100 | Hs.46701 | SNTB1 | 6641 | 8 | 8q23-q24 |
| transmembrane protein 68 | AI890761 | Hs.420076 | TMEM68 | 137695 | 8 | 8q12.1 |
| non-SMC element 2, MMS21 homolog (S. cerevisiae) | AL562908 | Hs.388297 | NSMCE2 | 286053 | 8 | 8q24.13 |
| CTF18, chromosome transmission fidelity factor 18 homolog (S. cerevisiae) | AK024476 | Hs.153850 | CHTF18 | 63922 | 16 | 16p13.3 |
| ankyrin repeat domain 10 | BE670056 | Hs.525163 | ANKRD10 | 55608 | 13 | 13q34 |
| zinc finger protein 251 | W93231 | Hs.534516 | ZNF251 | 90987 | 8 | 8q24.3 |
| sprouty-related, EVH1 domain containing 1 | BE967019 | Hs.525781 | SPRED1 | 161742 | 15 | 15q14 |
| casein kinase 1, epsilon | T51255 | Hs.474833 | CSNK1E | 1454 | 22 | 22q13.1 |
| aspartate beta-hydroxylase domain containing 2 | BE550881 | Hs.567547 | ASPHD2 | 57168 | 22 | 22q12.1 |
| zinc finger protein 697 | AW003092 | Hs.381105 | ZNF697 | 90874 | 1 | 1p12 |
| dual specificity phosphatase 18 | BC004110 | Hs.517544 | DUSP18 | 150290 | 22 | 22q12.2 |
| metal-regulatory transcription factor 1 | N46867 | Hs.471991 | MTF1 | 4520 | 1 | 1p33 |
| nudE nuclear distribution gene E homolog 1 (A. nidulans) | AI857685 | Hs.655378 | NDE1 | 54820 | 16 | 16p13.11 |
| INO80 complex subunit E | AA743390 | Hs.434864 | INO80E | 283899 | 16 | 16p11.2 |
| major facilitator superfamily domain containing 3 | AA523543 | Hs.7678 | MFSD3 | 113655 | 8 | 8q24.3 |
| IKAROS family zinc finger 1 (Ikaros) | AI741188 | Hs.435949 | IKZF1 | 10320 | 7 | 7p13-p11.1 |
| prolyl-tRNA synthetase 2, mitochondrial (putative) | AW409848 | Hs.380169 | PARS2 | 25973 | 1 | 1p32.2 |
| chromosome 16 open reading frame 52 | H06491 | Hs.498890 | C16orf52 | 730094 | 16 | 16p12.2 |
| zinc finger, MYM-type 6 | AI743551 | Hs.533986 | ZMYM6 | 9204 | 1 | 1p34.2 |
| zinc finger protein 326 | AI057121 | Hs.306221 | ZNF326 | 284695 | 1 | 1p22.2 |
| proprotein convertase subtilisin/kexin type 9 | W92036 | Hs.18844 | PCSK9 | 255738 | 1 | 1p32.3 |
| WD repeat domain 90 | AL043021 | Hs.511903 | WDR90 | 197335 | 16 | 16p13.3 |
| eukaryotic translation initiation factor 2C, 4 | AI669957 | Hs.471492 | EIF2C4 | 192670 | 1 | 1p34.3 |
| chromosome 22 open reading frame 36 | AW006520 | Hs.113314 | C22orf36 | 388886 | 22 | 22q11.23 |
| ubiquinol-cytochrome c reductase, complex III subunit X | BE208777 | Hs.284292 | UQCR10 | 29796 | 22 | 22cen-q12.3 |
| alpha-kinase 3 | AA843297 | Hs.459183 | ALPK3 | 57538 | 15 | 15q25.2 |
| E2F transcription factor 2 | AL561296 | Hs.194333 | E2F2 | 1870 | 1 | 1p36 |
| RNA binding motif protein 15 | AI092824 | Hs.435947 | RBM15 | 64783 | 1 | 1p13 |
| transmembrane protein 219 | AA496243 | Hs.460574 | TMEM219 | 124446 | 16 | 16p11.2 |
| aarF domain containing kinase 5 | BE856488 | Hs.283374 | ADCK5 | 203054 | 8 | 8q24.3 |
| reticulon 4 receptor | NM_023004 | Hs.30868 | RTN4R | 65078 | 22 | 22q11.21 |
| BTB (POZ) domain containing 11 | BF510581 | Hs.271272 | BTBD11 | 121551 | 12 | 12q23.3 |
| aurora kinase A interacting protein 1 | BE463815 | Hs.632515 | AURKAIP1 | 54998 | 1 | 1p36.33 |
| BAI1-associated protein 2-like 2 | AA532851 | Hs.474822 | BAIAP2L2 | 80115 | 22 | 22q13.1 |
| zinc finger and BTB domain containing 8 opposite strand | BG249305 | Hs.655921 | ZBTB8OS | 339487 | 1 | 1p35.1 |
| small nucleolar RNA host gene 12 (non-protein coding) | AI339426 | Hs.632377 | SNHG12 | 85028 | 1 | 1p35.3 |
| chromosome 7 open reading frame 31 | AA778688 | Hs.122055 | C7orf31 | 136895 | 7 | 7p15.3 |
| GNAS complex locus | AI693143 | Hs.125898 | GNAS | 2778 | 20 | 20q13.3 |
| 4-hydroxyphenylpyruvate dioxygenase-like | AI653050 | Hs.162717 | HPDL | 84842 | 1 | 1p34.1 |
| syntaxin 4 | BF939292 | Hs.83734 | STX4 | 6810 | 16 | 16p11.2 |
| nardilysin (N-arginine dibasic convertase) | AA448346 | Hs.584782 | NRD1 | 4898 | 1 | 1p32.2-p32.1 |
| vang-like 1 (van gogh, Drosophila) | R85437 | Hs.515130 | VANGL1 | 81839 | 1 | 1p13.1 |
| slowmo homolog 2 (Drosophila) | AA004210 | Hs.726261 | SLMO2 | 51012 | 20 | 20q13.32 |
| ubiquitin specific peptidase 24 | AA002182 | Hs.477009 | USP24 | 23358 | 1 | 1p32.3 |
| ataxin 7-like 2 | N93313 | Hs.118248 | ATXN7L2 | 127002 | 1 | 1p13.3 |
| mucolipin 2 | AV713773 | Hs.591446 | MCOLN2 | 255231 | 1 | 1p22 |
| PRP38 pre-mRNA processing factor 38 (yeast) domain containing B | N32872 | Hs.342307 | PRPF38B | 55119 | 1 | 1p13.3 |
| chromosome 7 open reading frame 30 | BF062037 | Hs.87385 | C7orf30 | 115416 | 7 | 7p15.3 |
| chromosome 22 open reading frame 27 | AI806805 | Hs.592202 | C22orf27 | 150291 | 22 | 22q12.2 |
| farnesyltransferase, CAAX box, alpha | AA833870 | Hs.370312 | FNTA | 2339 | 8 | 8p11 |
| ATPase, class VI, type 11A | AW068936 | Hs.29189 | ATP11A | 23250 | 13 | 13q34 |
| ubiquitin specific peptidase 7 (herpes virus-associated) | BF433061 | Hs.386939 | USP7 | 7874 | 16 | 16p13.3 |
| POM121 membrane glycoprotein-like 10, pseudogene | AL039389 | Hs.531306 | POM121L10P | 646074 | 22 | 22q11.23 |
| proteasome (prosome, macropain) subunit, beta type, 2 | AW043830 | Hs.471441 | PSMB2 | 5690 | 1 | 1p34.2 |
| zyg-11 homolog A (C. elegans) | AW243917 | Hs.658458 | ZYG11A | 440590 | 1 | 1p32.3 |
| adaptor-related protein complex 4, beta 1 subunit | NM_006594 | Hs.515048 | AP4B1 | 10717 | 1 | 1p13.2 |
| myosin light chain kinase 2 | AF325549 | Hs.86092 | MYLK2 | 85366 | 20 | 20q13.31 |
| ankyrin repeat domain 54 | Z97630 | Hs.135259 | ANKRD54 | 129138 | 22 | 22q13.1 |
| outer dense fiber of sperm tails 2-like | AB033055 | Hs.149360 | ODF2L | 57489 | 1 | 1p22.3 |
| myotubularin related protein 14 | AI888303 | Hs.475382 | MTMR14 | 64419 | 3 | 3p26 |
| coiled-coil domain containing 18 | AL139421 | Hs.728345 | CCDC18 | 343099 | 1 | 1p22.1 |
| F-box and leucine-rich repeat protein 20 | AK024690 | Hs.462946 | FBXL20 | 84961 | 17 | 17q12 |
| SRC kinase signaling inhibitor 1 | BF062187 | Hs.448872 | SRCIN1 | 80725 | 17 | 17q12 |
| zinc finger protein 684 | AW275016 | Hs.524767 | ZNF684 | 127396 | 1 | 1p34.2 |
| APAF1 interacting protein | AF131812 | Hs.447794 | APIP | 51074 | 11 | 11p13 |
| collagen, type XX, alpha 1 | BF344604 | Hs.271285 | COL20A1 | 57642 | 20 | 20q13.33 |
| zinc finger protein 691 | AK000938 | Hs.20879 | ZNF691 | 51058 | 1 | 1p34.2 |
| dedicator of cytokinesis 9 | AU146550 | Hs.596105 | DOCK9 | 23348 | 13 | 13q32.3 |
| ubiquitin protein ligase E3 component n-recognin 4 | AU145867 | Hs.148078 | UBR4 | 23352 | 1 | 1p36.13 |
| isoprenylcysteine carboxyl methyltransferase | AL117548 | Hs.515688 | ICMT | 23463 | 1 | 1p36.21 |
| selenoprotein O | BC001099 | Hs.365405 | SELO | 83642 | 22 | 22q13.33 |
| FAD-dependent oxidoreductase domain containing 2 | AL022313 | Hs.387601 | FOXRED2 | 80020 | 22 | 22q12.3 |
| basic, immunoglobulin-like variable motif containing | AF339793 | Hs.288809 | BIVM | 54841 | 13 | 13q33.1 |
| KIAA1875 | AL137446 | Hs.98723 | KIAA1875 | 340390 | 8 | 8q24.3 |
| non-protein coding RNA 176 | AL157500 | Hs.97840 | NCRNA00176 | 284739 | 20 | 20q13.33 |
| chromosome 1 open reading frame 201 | AK025957 | Hs.403187 | C1orf201 | 90529 | 1 | 1p36.11 |
| ADP-ribosylation factor GTPase activating protein 1 | AL137744 | Hs.25584 | ARFGAP1 | 55738 | 20 | 20q13.33 |
| sel-1 suppressor of lin-12-like 2 (C. elegans) | AL137678 | Hs.590879 | SEL1L2 | 80343 | 20 | 20p12.1 |
| basic helix-loop-helix family, member e23 | BF513872 | Hs.551230 | BHLHE23 | 128408 | 20 | 20q13.33 |
| src-related kinase lacking C-terminal regulatory tyrosine and N-terminal myristylation sites | AL121829 | Hs.411061 | SRMS | 6725 | 20 |  |
| R3H domain containing-like | AL117382 | Hs.580807 | R3HDML | 140902 | 20 | 20q13.12 |
| ORM1-like 3 (S. cerevisiae) | BF337528 | Hs.514151 | ORMDL3 | 94103 | 17 | 17q12 |
| excision repair cross-complementing rodent repair deficiency, complementation group 4 | AI694544 | Hs.567265 | ERCC4 | 2072 | 16 | 16p13.12 |
| bone morphogenetic protein 8b | AA610122 | Hs.664022 | BMP8B | 656 | 1 | 1p35-p32 |
| abhydrolase domain containing 13 | AA404347 | Hs.183528 | ABHD13 | 84945 | 13 | 13q33.3 |
| fucosyltransferase 10 (alpha (1,3) fucosyltransferase) | AI147738 | Hs.458713 | FUT10 | 84750 | 8 | 8p12 |
| mitochondrial methionyl-tRNA formyltransferase | AL563572 | Hs.531615 | MTFMT | 123263 | 15 | 15q22.31 |
| glucosidase, alpha; neutral C | AA203132 | Hs.143261 | GANC | 2595 | 15 | 15q15.2 |
| phosphoinositide-3-kinase, catalytic, alpha polypeptide | AA767763 | Hs.553498 | PIK3CA | 5290 | 3 | 3q26.3 |
| glutamate-cysteine ligase, modifier subunit | AI753488 | Hs.315562 | GCLM | 2730 | 1 | 1p22.1 |
| WD repeat domain 8 | AW572779 | Hs.31714 | WDR8 | 49856 | 1 | 1p36.3 |
| acyl-CoA thioesterase 8 | AI885067 | Hs.444776 | ACOT8 | 10005 | 20 | 20q13.12 |
| coiled-coil domain containing 78 | AW264810 | Hs.381943 | CCDC78 | 124093 | 16 | 16p13.3 |
| tripartite motif containing 62 | AI479391 | Hs.656006 | TRIM62 | 55223 | 1 | 1p35.1 |
| chromosome 22 open reading frame 43 | AA857437 | Hs.517466 | C22orf43 | 51233 | 22 | 22q11.2 |
| chromosome 1 open reading frame 174 | BF970340 | Hs.103939 | C1orf174 | 339448 | 1 | 1p36.32 |
| ADP-ribosylhydrolase like 1 | AI243209 | Hs.98669 | ADPRHL1 | 113622 | 13 | 13q34 |
| transcription elongation factor A (SII), 2 | AI393116 | Hs.505004 | TCEA2 | 6919 | 20 | 20q13.33 |
| family with sequence similarity 70, member B | AA974579 | Hs.280805 | FAM70B | 348013 | 13 | 13q34 |
| nuclear transcription factor Y, gamma | AV700263 | Hs.713051 | NFYC | 4802 | 1 | 1p32 |
| cytochrome b5 reductase-like | BF438410 | Hs.591426 | CYB5RL | 606495 | 1 | 1p32.3 |
| citrate lyase beta like | BG398847 | Hs.655642 | CLYBL | 171425 | 13 | 13q32 |
| zinc finger protein 720 | AA744964 | Hs.528826 | ZNF720 | 124411 | 16 | 16p11.2 |
| UTP23, small subunit (SSU) processome component, homolog (yeast) | BE542779 | Hs.86970 | UTP23 | 84294 | 8 | 8q24.11 |
| leucine-rich repeats and immunoglobulin-like domains 2 | BF967997 | Hs.448972 | LRIG2 | 9860 | 1 | 1p13.1 |
| ATP binding domain 4 | BE884544 | Hs.107196 | ATPBD4 | 89978 | 15 | 15q14 |
| tropomyosin 1 (alpha) | AI521618 | Hs.133892 | TPM1 | 7168 | 15 | 15q22.1 |
| tyrosyl-tRNA synthetase | AW452122 | Hs.213264 | YARS | 8565 | 1 | 1p35.1 |
| KIAA1429 | AA455079 | Hs.202238 | KIAA1429 | 25962 | 8 | 8q22.1 |
| serine/arginine-rich splicing factor 4 | R05895 | Hs.469970 | SRSF4 | 6429 | 1 | 1p35.3 |
| hypothetical locus LOC441204 | AA406397 | Hs.587432 | LOC441204 | 441204 | 7 | 7p15.2 |
| G1 to S phase transition 1 | AA580082 | Hs.528780 | GSPT1 | 2935 | 16 | 16p13.1 |
| C2 calcium-dependent domain containing 4A | BE218239 | Hs.202656 | C2CD4A | 145741 | 15 | 15q22.2 |
| zinc finger protein 517 | H46217 | Hs.521942 | ZNF517 | 340385 | 8 | 8q24.3 |
| tRNA splicing endonuclease 54 homolog (S. cerevisiae) | AA504269 | Hs.378501 | TSEN54 | 283989 | 17 | 17q25.1 |
| forkhead box D3 | AI867445 | Hs.546573 | FOXD3 | 27022 | 1 | 1p31.3 |
| tripartite motif containing 45 | AI793200 | Hs.301526 | TRIM45 | 80263 | 1 | 1p13.1 |
| claspin | AI304876 | Hs.175613 | CLSPN | 63967 | 1 | 1p34.2 |
| myocyte enhancer factor 2A | AA491228 | Hs.268675 | MEF2A | 4205 | 15 | 15q26 |
| iron-responsive element binding protein 2 | AW470799 | Hs.436031 | IREB2 | 3658 | 15 | 15q25.1 |
| coiled-coil and C2 domain containing 1B | AW961746 | Hs.591451 | CC2D1B | 200014 | 1 | 1p32.3 |
| GLI family zinc finger 4 | BF002450 | Hs.400533 | GLI4 | 2738 | 8 | 8q24.3 |
| testis expressed 9 | AA020920 | Hs.511476 | TEX9 | 374618 | 15 | 15q21.3 |
| F-box protein 7 | AI678692 | Hs.5912 | FBXO7 | 25793 | 22 | 22q12-q13 |
| TTC28 antisense RNA 1 (non-protein coding) | AI888657 | Hs.602319 | TTC28-AS1 | 284900 | 22 | 22q12.1 |
| myosin IG | BE646398 | Hs.37617 | MYO1G | 64005 | 7 | 7p13-p11.2 |
| glycerophosphodiester phosphodiesterase domain containing 5 | AL041124 | Hs.503297 | GDPD5 | 81544 | 11 | 11q13.4-q13.5 |
| stratifin | X57348 | Hs.523718 | SFN | 2810 | 1 | 1p36.11 |
| ring finger protein 19B | W27419 | Hs.591504 | RNF19B | 127544 | 1 | 1p35.1 |
| ATPase, H+ transporting, lysosomal 16kDa, V0 subunit c | M62762 | Hs.389107 | ATP6V0C | 527 | 16 | 16p13.3 |
| calcineurin binding protein 1 | AB002328 | Hs.517478 | CABIN1 | 23523 | 22 | 22q11.23 |
| potassium channel tetramerisation domain containing 13 | AW026481 | Hs.534590 | KCTD13 | 253980 | 16 | 16p11.2 |
| proline rich 5 (renal) | AA533284 | Hs.102336 | PRR5 | 55615 | 22 | 22q13 |
| acetyl-CoA carboxylase beta | AI057637 | Hs.234898 | ACACB | 32 | 12 | 12q24.11 |
| zinc finger protein 764 | AA746290 | Hs.132227 | ZNF764 | 92595 | 16 | 16p11.2 |
| polymerase (RNA) II (DNA directed) polypeptide J4, pseudogene | AA601208 | Hs.657028 | POLR2J4 | 84820 | 7 | 7p13 |
