# Supplementary material for: Concurrent Gene Signatures for Han Chinese Breast Cancers
Source: PLoS One. 2013 Oct 3;8(10):e76421. doi: 10.1371/journal.pone.0076421 (PMC3789693; doi:10.1371/journal.pone.0076421)
Supplement: Table S3 — Molecular subtyping of the 408 Taiwanese breast cancers with the Stanford/UNC intrinsic genes: (A) molecular subtyping of the 81 Taiwanese breast cancers from the current study, (B) molecular subtyping of the 327 Taiwanese breast cancers from Kao et al., and (C) molecular subtyping of the combined dataset of 408 Taiwanese breast cancers. (DOCX) [file pone.0076421.s013.docx]

**Supplemental Table 3. Molecular subtyping of the 408 Taiwanese breast cancers with the Stanford/UNC intrinsic genes.**

(A) Molecular subtyping of the 81 Taiwanese breast cancers from the current study with the Stanford/UNC intrinsic genes.

| **Phenotype** | **Molecular subtype*** | | | | | | |
| --- | --- | --- | --- | --- | --- | --- | --- |
|  | **Basal** | **HER2** | **LumA** | **LumB** | **Norm** | **Uncla** | **Total** |
| **ER+HER+** | 0 | 5 | 2 | 7 | 1 | 1 | 16 |
| **ER+HER2-** | 0 | 0 | 20 | 8 | 5 | 4 | 37 |
| **ER-HER2+** | 4 | 8 | 0 | 0 | 5 | 1 | 18 |
| **ER-HER2-** | 6 | 1 | 0 | 0 | 2 | 1 | 10 |
| **Total** | 10 | 14 | 22 | 15 | 13 | 7 | 81 |

(B) Molecular subtyping of the 327 Taiwanese breast cancers from Kao et al. with the Stanford/UNC intrinsic genes.

| **Phenotype** | **Molecular subtype*** | | | | | | |
| --- | --- | --- | --- | --- | --- | --- | --- |
|  | **Basal** | **HER2** | **LumA** | **LumB** | **Norm** | **Uncla** | **Total** |
| **ER+HER+** | 0 | 10 | 3 | 9 | 0 | 0 | 22 |
| **ER+HER2-** | 0 | 2 | 98 | 50 | 12 | 8 | 170 |
| **ER-HER2+** | 17 | 26 | 2 | 2 | 9 | 1 | 57 |
| **ER-HER2-** | 43 | 6 | 6 | 5 | 15 | 3 | 78 |
| **Total** | 60 | 44 | 109 | 66 | 36 | 12 | 327 |

(C) Molecular subtyping of the combined dataset of 408 Taiwanese breast cancers with the Stanford/UNC intrinsic genes.

| **Phenotype** | **Molecular subtype*** | | | | | | |
| --- | --- | --- | --- | --- | --- | --- | --- |
|  | **Basal** | **HER2** | **LumA** | **LumB** | **Norm** | **Uncla** | **Total** |
| **ER+HER+** | 0 | 15 | 5 | 16 | 1 | 1 | 38 |
| **ER+HER2-** | 0 | 2 | 118 | 58 | 17 | 12 | 207 |
| **ER-HER2+** | 21 | 34 | 2 | 2 | 14 | 2 | 75 |
| **ER-HER2-** | 49 | 7 | 6 | 5 | 17 | 4 | 88 |
| **Total** | 70 | 58 | 131 | 81 | 49 | 19 | 408 |

(*Basal: basal-like, HER2: HER2-enriched, LumA: luminal A, LumB: luminal B, Norm: normal breast-like, Uncla: unclassified molecular subtype)
