# Supplementary material for: Concurrent Gene Signatures for Han Chinese Breast Cancers
Source: PLoS One. 2013 Oct 3;8(10):e76421. doi: 10.1371/journal.pone.0076421 (PMC3789693; doi:10.1371/journal.pone.0076421)
Supplement: Table S4 — Predictive of ER and HER2 signatures in training and independent data: (A) predictive accuracy of ER signature, and (B) predictive accuracy of HER2 signature. (DOCX) [file pone.0076421.s014.docx]

**Supplemental Tables S4. Predictive of ER and HER2 signatures in training and independent data.**

(A) Predictive accuracy of ER signature in training and independent data.

|  | Training | Independent dataset | Training | Independent dataset |
| --- | --- | --- | --- | --- |
|  | dataset |  | dataset |  |
|  | Current study | GSE5460 | GSE5460 | Current study |
| ER+/ER- | 53/28 | 74/51 | 74/51 | 53/28 |
| Number of signature genes | 49 | 49 | 252 | 252 |
|  |  |  |  |  |
| **Classifiers** |  |  |  |  |
| Compound covariate predictor | 81% | 69% | 92% | 63% |
|  |  |  |  |  |
| Diagonal linear discriminative analysis | 81% | 67% | 90% | 64% |
|  |  |  |  |  |
| 3-nearest neighbors | **84%*** | **83%*** | **95%*** | **81%*** |
|  |  |  |  |  |
| Nearest centroid | 83% | 71% | 94% | 62% |
|  |  |  |  |  |
| Supportive vector machines | 80% | 80% | 93% | 78% |

(B) Predictive accuracy of HER2 signature in training and independent data.

|  | Training | Independent dataset | Training | Independent dataset |
| --- | --- | --- | --- | --- |
|  | dataset |  | dataset |  |
|  | Current study | GSE5460 | GSE5460 | Current study |
| HER2+/HER2- | 34/47 | 30/95 | 30/95 | 34/47 |
| Number of signature genes | 16 | 16 | 43 | 43 |
|  |  |  |  |  |
| **Classifiers** |  |  |  |  |
| Compound covariate predictor | 77% | 50% | 93% | 72% |
|  |  |  |  |  |
| Diagonal linear discriminative analysis | 75% | 34% | 92% | 72% |
|  |  |  |  |  |
| 3-nearest neighbors | 69% | **66%*** | **94%*** | **74%*** |
|  |  |  |  |  |
| Nearest centroid | **78%*** | 61% | 93% | 73% |
|  |  |  |  |  |
| Supportive vector machines | 68% | 35% | 90% | 63% |

(*best predictive results)
